# Intrinsic and Extrinsic Thermodynamics for Stochastic Population Processes with Multi-Level Large-Deviation Structure

**DOI:** 10.3390/e22101137

**Published:** 2020-10-07

**Authors:** Eric Smith

**Affiliations:** 1 Department of Biology, Georgia Institute of Technology, 310 Ferst Drive NW, Atlanta, GA 30332, USA; desmith@santafe.edu; 2 Earth-Life Science Institute, Tokyo Institute of Technology, 2-12-1-IE-1 Ookayama, Meguro-ku, Tokyo 152-8550, Japan; 3 Santa Fe Institute, 1399 Hyde Park Road, Santa Fe, NM 87501, USA; 4 Ronin Institute, 127 Haddon Place, Montclair, NJ 07043, USA

**Keywords:** non-equilibrium thermodynamics, large-deviation theory, stochastic processes, chemical reaction networks, emergence of macroworlds

## Abstract

A set of core features is set forth as the essence of a thermodynamic description, which derive from large-deviation properties in systems with hierarchies of timescales, but which are not dependent upon conservation laws or microscopic reversibility in the substrate hosting the process. The most fundamental elements are the concept of a macrostate in relation to the large-deviation entropy, and the decomposition of contributions to irreversibility among interacting subsystems, which is the origin of the dependence on a concept of heat in both classical and stochastic thermodynamics. A natural decomposition that is known to exist, into a relative entropy and a housekeeping entropy rate, is taken here to define respectively the *intensive* thermodynamics of a system and an *extensive* thermodynamic vector embedding the system in its context. Both intensive and extensive components are functions of Hartley information of the momentary system stationary state, which is information about the joint effect of system processes on its contribution to irreversibility. Results are derived for stochastic chemical reaction networks, including a Legendre duality for the housekeeping entropy rate to thermodynamically characterize fully-irreversible processes on an equal footing with those at the opposite limit of detailed-balance. The work is meant to encourage development of inherent thermodynamic descriptions for rule-based systems and the living state, which are not conceived as reductive explanations to heat flows.

## 1. Introduction

The statistical derivations underlying most thermodynamic phenomena are understood to be widely applicable, and are mostly developed in general terms. Yet, where thermodynamics is offered as an ontology to understand new patterns and causes in nature—the thermodynamics of computation [1,2,3,4] or stochastic thermodynamics [5], or where these methods are taken to define a foundation for the statistical physics of reproduction or adaptation [6,7]—these problems are framed in terms of two properties particular to the domain of mechanics: the conservation of energy and microscopic reversibility. Other applications of the same mathematics, with designations such as intensity of choice [8,9] tipping points, or early warning signs [10], are recognized as analogies to thermodynamics, on the understanding that they only become the “thermodynamics of” something when they derive its causes from energy conservation connected to the entropies of heat.

Particularly active today, stochastic thermodynamics is the modern realization of a program to create a non-equilibrium thermodynamics that began with Onsager [11,12] and took much of its modern form under Prigogine and coworkers [13,14]. Since the beginning, its core method has been to derive rules or constraints for non-stationary thermal dynamics from dissipation of free energies defined by Gibbs equilibria, from any combination of thermal baths or asymptotic reservoirs.

The parallel and contemporaneous development of thermodynamics of computation can be viewed as a quasistatic analysis with discrete changes in the boundary conditions on a thermal bath corresponding to logical events in algorithms. Early stochastic thermodynamics combined elements of both traditions, with the quantities termed “non-equilibrium entropies” corresponding to the information entropies over computer states, distinct from quasistatic entropies associated with heat in a locally-equilibrium environment, with boundary conditions altered by the explicitly-modeled stochastic state transitions. More recently, stochastic thermodynamics incorporated time-reversal methods originally developed for measures in dynamical systems [15,16,17,18] leading to a variety of fluctuation theorems [19,20,21] and non-equilibrium work relations [22,23,24], still, however, relating path probability ratios either to dissipation of heat or to differences in equilibrium free energies.

This accepted detachment of mathematics from phenomenology, with thermodynamic phenomena interpreted in their historical terms and mathematics kept interpretation-free, contrasts with the way statistical mechanics was allowed to expand the ontological categories of physics at the end of the 19th century. Although the large-deviation rate function underlies the much earlier solution of the gambler’s ruin problem of Pascal, Fermat, and Bernoulli [25], the kinetic theory of heat [26,27,28] was not put forth as an analogy to gambling, used merely to describe patterns in the “actual” fluid caloric. Thermodynamics, instead, took the experienced phenomena involving heat, and where there had formerly been only names and metaphors to refer to them, it brought into existence concepts capturing their essential nature, not dissolving their reality as phenomena [29], but endowing it with a semantics.

(Note, however, that as late as 1957, Jaynes [30,31] needed to assert that the information entropy of Shannon referred to the same quantity as the physical entropy of Gibbs, and was not merely the identical mathematical function. Even the adoption of “entropy production” to refer to changes in the state-variable entropy by irreversible transformations—along the course of which the state-variable entropy is not even defined—is a retreat to a substance syntax. Lieb and Yngvason [32] observe that “caloric” accounts for physics at a macroscopic level just as well as “heat” does. This author would prefer the less euphonious but categorically better expression “loss of large-deviation accessibility” to replace “entropy production”. In limits where the large-deviation scale factor becomes infinite, a notion of *adiabatic accessibility* as an all-or-nothing distinction has been used as a formal foundation for a classical thermodynamics of equilibrium [32] and non-equilibrium [33,34] systems. Here the attracting manifold of the exponential family that defines large-deviation scale factors will be the central object of interest, precisely so that the scale factor does not need to be made infinite, so “loss of large-deviation accessibility” is meant in probability and not in absolute terms.)

The distinction is between formalization in the service of reduction to remain within an existing ontology, and formalization as the foundation for discovery of new conceptual primitives. Through generalizations and extensions that could not have been imagined in the late 19th century [35,36,37,38] (revisited in Section 7), it went on to do the same for our fundamental theory of objects and interactions, the nature of the vacuum and the hierarchy of matter, and the presence of stable macro-worlds at all.

This paper is written with the view that the essence of a thermodynamic description is not found in its connection to conservation laws, microscopic reversibility, or the equilibrium state relations they entail, despite the central role those play in the fields mentioned [2,5,6,7]. At the same time, it grants an argument that has been maintained across a half-century of enormous growth in both statistical methods and applications [39,40]: that thermodynamics should not be conflated with its statistical methods. The focus will therefore be on the patterns and relations that make a phenomenon essentially thermodynamic, in which statistical mechanics made it possible to articulate as concepts. The paper proposes a sequence of these and exhibits constructions of them unconnected to energy conservation or microscopic reversibility.

Essential concepts are of three kinds: (1) the nature and origin of macrostates; (2) the roles of entropy in relation to irreversibility and fluctuation; and (3) the natural apportionment of irreversibility between a system and its environment, which defines an intrinsic thermodynamics for the system and an extrinsic thermodynamics that embeds the system in its context, in analogy to the way differential geometry defines an intrinsic curvature for a manifold distinct from extrinsic curvatures that may embed the manifold in another manifold of higher dimension. (This analogy is not a reference to natural information geometries [41,42] that can also be constructed, though perhaps those geometries would provide an additional layer of semantics to the constructions here.)

All of these concepts originate in the large-deviation properties of stochastic processes with multi-level timescale structure. They will be demonstrated here using a simple class of stochastic population processes, further specified as chemical reaction network models as more complex relations need to be presented.

To summarize briefly and intuitively, both microstate and macrostate are concepts applied relative to the dynamical level under consideration in a stochastic process. Within a given level, they are different classes of entities: macrostates are a special class of distributions over microstates, specificiable by a fixed collection of statistical central tendencies decoupled from the exact scale that separates micro and macro. The separation of scale from structure is the defining scaling relation for large-deviations [43]. The more formal construction is explained in Section 3.2.

The roles of entropy are more difficult to summarize in short-hand, as a number of distinct patterns are present in the relation of thermal descriptions at the mesoscale and its associated macroscale. It will be necessary in the following to distinguish the roles of entropy as a functional on general distributions, introduced in Section 2.3, versus entropy as a state function on macrostates, and on the Lyapunov role of entropy in the 2nd law [28,39] versus its large-deviation role in fluctuations [44], through which the state-function entropy is most generally definable [43]. The latter two roles and their difference are treated starting in Section 4.2. Both Shannon’s information entropy [45] and the older Hartley function [46] will appear here as they do in stochastic thermodynamics generally [5]. The different meanings these functions carry, which sound contradictory when described and can be difficult to compare in derivations with different aims, become clear as each appears in the course of a single calculation. Most important, they have unambiguous roles in relation to either large deviations or system decomposition without need of a reference to heat.

### 1.1. On the History of the Development of Ideas Used Here, and a Transition from Mechanical to Large-Deviation Perspectives

This section provides a brief summary of ideas and literature where results presented below were first derived, and an explanation of what the current paper contributes in addition. In the interest of brevity, some technical references are made to methods that have not been didactically introduced above, but are demonstrated in later sections. It may be easier to read the constructive sections of the paper and then revisit this summary to follow the motivation for connections claimed here.

#### 1.1.1. On the Additive Decomposition of Total Entropy Change

The large body of work accumulated since the late 1990s in what could be called the thermodynamics of the mesoscale contains most technical results on entropy decomposition used below, derived in the language of fluctuation theorems and generally framed in energy equivalents. Fluctuation theorems and non-equilibrium work relations connect historically salient questions about limits to work that grow naturally out of mechanics, with a distinct set of questions about partitioning of sources of irreversibility that are more central from a large-deviation perspective.

Several authors have argued [21,32,47,48,49] that the founding efforts to formalize a 2nd law recognize not one, but two categories or origins of irreversibility. One is the formulation of Clausius, related to irreversibility of transients, the other is due to Kelvin and Planck and related to constitutional irreversibility of transitions in driven systems.

Recognizing that constitutional irreversibility interferes with extension of the usual Gibbs free energy change to characterize transients, Oono and Paniconi [50] argued for an additive separation of the two components of dissipation, introducing housekeeping heat (constitutive) phenomenologically in terms of quasistatic transformations through non-equilibrium steady states, with excess heat (transient) left to extend Gibbs free energy to non-equilibrium relaxation. The non-negativity of housekeeping heat, independent of the presence or rate of transients, was established with a fluctuation theorem by Hatano and Sasa [51], with subsequent treatment by Speck and Seifert [52] and others [21,53]. (Understanding of the essence of a non-equilibrium or generalized thermodynamics has developed on one hand within a deliberately phenomenological track as in [40,50] and many parts of [54], and in parallel with the support of underlying statistical mechanics as in [21,51,52,53] and most literature on stochastic thermodynamics.)

The division of total entropy change into two individually non-negative terms, and the perspective it gives on the generalization from equilibrium to non-equilibrium thermodynamics, were subsequently studied by numerous authors [21,47,48,49,54,55,56,57,58]. Chemical reaction systems of the kind studied here in Section 4 were considered in [54,57], and multi-level systems defined by separation of timescales were considered in [55]. The centrality of the large-deviation function for the stationary state was emphasized in all of [49,54,55,56,57]. While these treatments are carried out within a context of energy conservation and develop interpretations in terms of work and heat, it was noted that the fluctuation theorems themselves do not depend on conservation laws. The work overall represents a shift from a primarily mechanical framing to one emphasizing fluctuation accessibility.

Within classical thermodynamics, the lack of a definite sign for changes in Shannon entropy is the key feature of the theory [59] enabling thermal or chemical work or refrigeration. The Kelvin–Planck formulation of irreversibility does not arise, as driven systems are excluded from the scope of the theory, and the Clausius formulation does not arise, as the theory is organized around the sub-manifold of adiabatically interconvertible macrostates [39]. (For a modern treatment of equilibrium see [32], followed by discussion of the limits of equivalent methods for non-equilibrium in [33,34].)

When an explicit irreversible dynamics is introduced, however, the lack of definite sign change for both Shannon entropy and rejected environmental heat appears not as a feature but as a “deficiency that these quantities themselves do not obey a fluctuation theorem” [21]. Retrospectively, we see that for classical thermodynamics the deficiency was remedied by Gibbs [60] in the construction of the free energy as a relative entropy with a measure defined in terms of the constitutive parameters [61]. Adiabatic transformations exchange Shannon entropy for entropy associated with support in the Gibbs measure, now regarded as a property of the system, while the Gibbs free energy as a whole evolves unidirectionally in a spontaneous process under detailed balance. With respect to the construction of relative entropies, the Hartley function of non-equilibrium steady states of [50,51] then becomes the correct generalization of the Gibbs measure away from detailed balance.

It is emphasized in [48] that the log-steady-state distribution is a “non-local” function of the constitutive parameters, possibly never realized by the dynamics, and in [48,55] that thermodynamics derived from the rate equations within a system become conditionally independent of the origins of these constitutive parameters outside the system or at faster timescales, given the steady-state measure. The current paper encapsulates these observations in the view of entropy relative to the stationary state as the intrinsic thermodynamics closing the description of a mesoscale system, and of the housekeeping heat as embedding the system extrinsically in its environment. The intrinsic/extrinsic characterization substitutes for the non-adiabatic/adiabatic dichotomy emphasized in [21,50] that grew out of the organization of equilibrium thermodynamics in terms of adiabatic accessibility.

The same shift away from the classical thermodynamic emphasis on potentials generalizing the mechanical potential energy underlies our use of the steady-state Hartley measure as a connection rather than as a term in a time-dependent potential. In [47], the time derivative of the steady-state measure needed to be separated from the relative entropy in order to arrive at a fluctuation theorem. Within an energy framework, the reference measure is called a “boundary entropy” (their Equation (15)), and its time derivative a “driving entropy production” (their Equation (18)). The steady-state measure, used here as a connection, is not a potential term changing in time, but rather a tangent vector defining the null direction for changes in Shannon entropy of a system.

#### 1.1.2. On Subsuming all Entropy Concepts within a Uniform Large-Deviation Paradigm

The definition of entropy in terms of large deviations, with the Lyapunov property for relaxations derived as a consequence, follows the approach of [43]. The Hamilton–Jacobi and eikonal methods used to approximate large-deviation functions follow the established treatments in [40,57] and [62], respectively.

The emphasis in [43] on the large-deviation scaling relation and invariant manifold (related to the invariant manifold of renormalization-group flow [36,38]), rather than on the asymptotic limit of infinite scale separation, together with the use of eikonal methods, permits us a somewhat more flexible and more complete treatment of scale separation than that of [55], due to the following algebraic observation: In the analysis of multiscale systems, the large-deviation scaling parameter (e.g., volume or particle number) becomes the inverse of some weak coupling that defines the convergence of approximation methods (as in the derivation of the central-limit thoerem). As emphasized by Cardy [63] and Coleman [64] Ch. 7, expansions in moments of fluctuations are perturbation series in these weak couplings, but naïvely constructed they are only asymptotic expansions. The instanton trajectories associated with first passages underlie an expansion in essential singularities (terms of the form $e^{-1/g}$ in the perturbative coupling *g*), which must first be extracted to regularize perturbation expansions to render them convergent power series. The resulting analytic distinction formally separates the within-scale effects in perturbative expansions from the essential singularities carrying cross-level scale separations, for finite as well as asymptotic scale separations.

Thus, to compare approaches: in [55], fast and slow variables of indefinitely separated timescale were declared by hand, and the fast variables were then averaged over distributions conditioned on the values of slow variables. In the eikonal expansion developed here, the fixed points in the large-deviation analysis, and the connection of other states to them by relaxation rays, define the conditional structure of a chain rule for entropies in Section 3.2.2 and Section 4.2.2. Perturbative corrections can in principle be computed about instanton backgrounds that are merely rapid and need not be instantaneous, though in practice such calculations tend to be technically difficult [65]. We note, however, that a guiding criterion in the approach of [55] also applies here: the hierarchical conditional structure and the defining relations for a thermodynamic description are self-similar when applied to any scale. Thermal properties are instantiated distinctly at different scales, but they are conceptualized without reference to any particular scale.

Finally, the derivation of fluctuating state-function entropies from a single multiscale large-deviation function clarifies the use of the Hartley function as a random variable on trajectories, addressed in [58]. From an approach originating in accounting consistency between mesoscale and macroscale thermodynamics [5], the log-probability either of the distribution under study, or of a reference distribution, must be evaluated as a random variable along trajectories, so that the average will recover a Shannon entropy or relative entropy of the distribution of trajectory endpoints.

Though it has become a standard usage to refer to both the random variable and its average merely as “entropies”, four distinct meanings are in play in the meso-to-macro relation: (1) A relative entropy function of arbitrary multiscale distributions that increases deterministically; state-function entropies either as (2) large-deviation or (3) Lyapunov functions that fluctuate (with distinct dynamics for the two cases under finite-rate transitions, as shown in Section 4.2), and (4) Hartley information of a reference measure that may never be attained by the system’s dynamics [48]. The fourth form, interpreted in Section 7 as a measure derived from the generating parameters of the system with information about their integration, illustrates concretely the value of an informational interpretation of entropies as random variables, distinct from the dynamical interpretation [58].

### 1.2. Main Results and Order of the Derivation

Section 2 explains what is meant by multi-level systems with respect to a robust separation of timescales, and introduces a family of models constructed recursively from nested population processes. Macrostates are related to microstates within levels, and if timescale separations arise that create new levels, they come from properties of a subset of long-lived, metastable macrostates. Such states within a level, and transitions between them that are much shorter than their characteristic lifetimes, map by coarse-graining to the elementary states and events at the next level.

The environment of a system that arises in some level of a hierarchical model is understood to include both the thermalized substrate at the level below, and other subsystems with explicit dynamics within the same level. Section 2.4 introduces the problem of system/environment partitioning of changes in entropy, and states the first main claim: that the natural partition is the one into a relative entropy [66] within the system and a housekeeping entropy rate [51] to embed the system in the environment. (The technical requirements are either evident or known from fluctuation theorems [52,53]; variant proofs are given in later sections. Their significance for system decomposition is the result of interest here.) It results in two independently non-negative entropy changes (plus a third entirely within the environment that is usually ignored), which define the intensive thermodynamics of the focal system and the extensive, or embedding thermodynamics of the system into the system⊗environment whole.

Section 3 expresses the relation of macrostates to microstates in terms of the large-deviations concept of *separation of scale from structure* [43]. Large-deviations scaling, defined as the convergence of distributions for aggregate statistics toward exponential families, creates a formal concept of a macroworld having definite structure, yet separated by an indefinite or even infinite range of scale from the specifications of micro-worlds.

Large-deviations for population processes are handled with the Hamilton–Jacobi theory [40] following from the time dependence of the cumulant-generating function (CGF), and its Legendre duality [41] to a large-deviation function called the *effective action* [67]. Macrostates are identified with the distributions that can be assigned large-deviation probabilities from a system’s stationary distribution. Freedom to study any CGF makes this definition, although concrete, flexible enough to require choosing what Gell-Mann and Lloyd [68,69] term “a judge”. The resulting definition, however, does not depend on whether the system has any underlying mechanics or conservation laws, explicit or implicit.

To make contact with multi-level dynamics and problems of interest in stochastic thermodynamics [5,70], while retaining a definite notation, Section 4 assumes generators of the form used for chemical reaction networks (CRNs) [71,72,73,74]. While numerous treatments of CRNs simply write down the stoichiometry and proceed directly to solve the chemical master equation [54,57,70], we appeal specifically to the ontology of species and complexes put forward by Horn and Jackson [71] and Feinberg [72], as the basis for this treatment. It both characterizes hypergraphs as generators for this distinctive class of systems, and when combined with the Doi operator algebra in Section 4.1, expresses the projection between the transition matrix in the state space, and the complex network in the generator, that will be the basis for cycle decompositions in Section 5. The finite-to-infinite mapping that relates macrostates to microstates has counterparts for CRNs in maps from the generator matrix to the transition matrix, and from mass-action macroscopic currents to probability currents between microstates.

This section formally distinguishes the Lyapunov [39] and large-deviation [44] roles of entropy, and shows how the definition of macrostates from Section 3 first introduces scale-dependence in the state-function entropy that was not present in the Lyapunov function for arbitrary multi-scale models of Section 2. In the Hamilton–Jacobi representation, Lyapunov and large-deviation entropy changes occur within different manifolds and have different interpretations. These differences are subordinate to the more fundamental difference between the entropy state function and entropy as a functional on arbitrary distributions over microstates. A properly formulated 2nd law, which is never violated, is computed from both deterministic and fluctuation macrostate-entropies. The roles of Hartley informations [46] for stationary states in stochastic thermodynamics [5,52,75] enter naturally as macrostate relative entropies.

Section 5 derives the proofs of monotonicity of the intrinsic and extrinsic entropy changes from Section 2, using a cycle decomposition of currents in the stationary distribution. The decomposition is related to that used by Schnakenberg [76] to compute dissipation in the stationary state, but more cycles are required in order to compute dynamical quantities. Whether only cycles or more complex hyperflows [77] are required in a basis for macroscopic currents distinguishes complexity classes for CRNs. Because cycles are always a sufficient basis in the microstate space, the breakdown of a structural equivalence between micro- and macro-states for complex CRNs occurs in just the terms responsible for the complex relations between state-function and global entropies derived in Section 4.

Section 6 illustrates two uses of the intensive/extensive decomposition of entropy changes. Some of the formalism in the earlier sections may seem more grounded with an example in mind, and readers who prefer this approach are encouraged to browse this section to see a fairly conservative example, still within domains commonly treated with stochastic thermodynamics [54,57,70], where the large-deviation emphasis provides more flexibility in handling irreversibility.

The section studies a simple model of polymerization and hydrolysis under competing spontaneous and driven reactions in an environment that can be in various states of disequilibrium. First a simple linear model of the kind treated by Schnakenberg [76] is considered, in a limit where one elementary reaction becomes strictly irreversible. Although the housekeeping entropy rate becomes uninformative because it is referenced to a diverging chemical potential, this is a harmless divergence analogous to a scale divergence of an extensive potential. A Legendre dual to the entropy rate remains regular, reflecting the existence of a thermodynamics-of-events about which energy conservation, although present on the path to the limit, asymptotically is not a source of any relevant constraints.

A second example introduces autocatalysis into the coupling to the disequilibrium environment, so that the system can become bistable. The components of entropy rate usually attributed to the “environment” omit information about the interaction of reactions responsible for the bistability, and attribute too much of the loss of large-deviation accessibility to the environment. Housekeeping entropy rate gives the correct accounting, recognizing that part of the system’s irreversibility depends on the measure for system relative entropy created by the interaction.

Section 7 offers an alternative characterization of thermodynamic descriptions when conservation and reversibility are not central. In 20th century physics the problem of the nature and source of macroworlds has taken on a clear formulation, and provides an alternative conceptual center for thermodynamics to relations between work and heat. System decomposition and the conditional independence structures within the loss of large-deviation accessibility replace adiabatic transformation and heat flow as the central abstractions to describe irreversibility, and define the entropy interpretation of the Hartley information. Such a shift in view will be needed if path ensembles are to be put on an equal footing with state ensembles to create a fully non-equilibrium thermodynamics. The alternative formulation is meant to support the development of a native thermodynamics of stochastic rule-based systems and a richer phenomenology of living states.

## 2. Multi-Level Systems

To provide a concrete class of examples for the large-deviation relations that can arise in multi-scale systems, we consider systems with natural levels, such that within each level a given system may be represented by a discrete population process. The population process in turn admits leading-exponential approximations of its large-deviation behavior in the form of a continuous dynamical system. These dynamical systems are variously termed momentum-space WKB approximations [78], Hamilton–Jacobi representations [40], or eikonal expansions [62]. They exist for many other classes of stochastic process besides the one assumed here [79,80], and may be obtained either directly from the Gärtner-Ellis theorem [44] for cumulant-generating functions (the approach taken here), by direct WKB asymptotics, or through saddle-point methods in 2-field functional integrals [67,81].

A connection between levels is made by supposing that the large-deviation behavior possesses one or more fixed points of the dynamical system. These are coarse-grained to become the elementary states in a similar discrete population process one level up in the scaling hierarchy. Nonlinearities in the dynamical system that produce multiple isolated, metastable fixed points are of particular interest, as transitions between these occur on timescales that are exponentially stretched, in the large-deviation scale factor, relative to the relaxation times. The resulting robust criterion for separation of timescales will be the basis for the thermodynamic limits that distinguish levels and justify the coarse-graining.

### 2.1. Micro to Macro, within and between Levels

**Figure 1:**
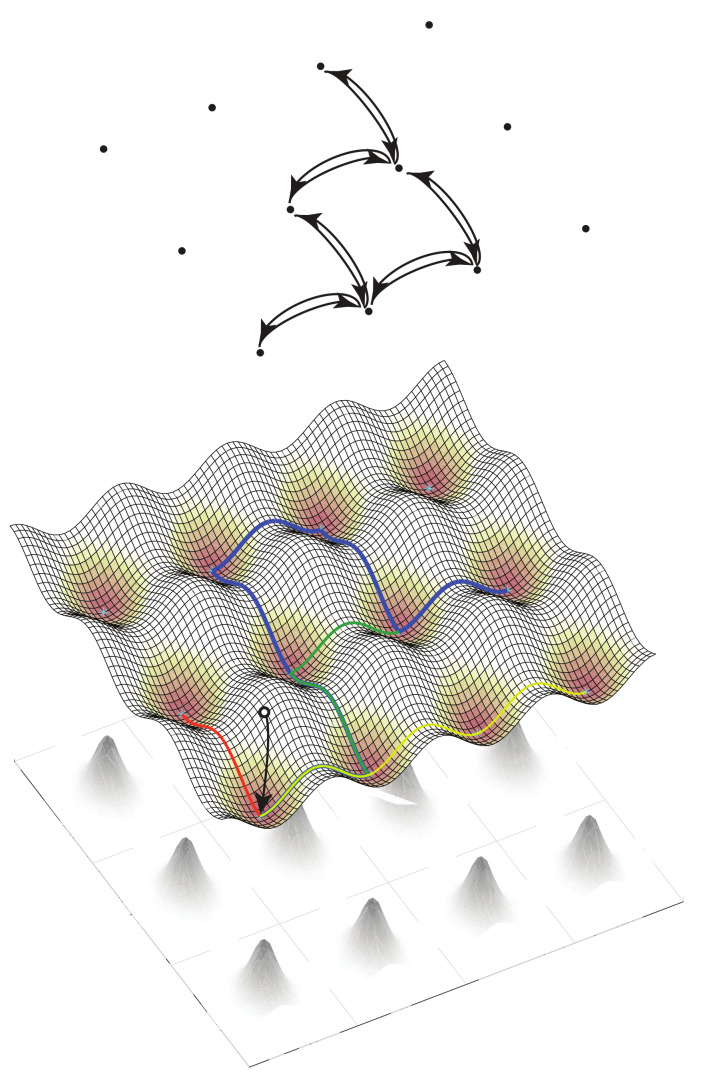
A multiscale population process with a lattice of fixed points. Middle layer is the grid of microstates and transitions in the mesoscale. Surface $-\log\underline{\rho }$ for a stationary distribution is indicated by the color gradient. Fixed points (asterisks) are at well bottoms. Modes of $\underline{\rho }$ concentrated near fixed points are indicated in greyscale beneath the surface. A classical deterministic trajectory (black, with arrow) starts at the open circle and relaxes to the fixed point for that basin. First-passage trajectories (colors) then mediate well switching by rare large deviations. Fixed points of trajectory equations in the mesoscale are coarse-grained to become elementary microstates (black dots) in the macroscale (top layer), and first passages become elementary transition events (arrows).

Models of this kind may be embedded recursively in scale through any number of levels. Here we will focus on three adjacent levels, diagrammed in Figure 1, and on the separation of timescales within a level, and the coarse-grainings that define the maps between levels. The middle level, termed the *mesoscale*, will be represented explicitly as a stochastic process, and all results that come from large-deviations scaling will be derived within this level. The *microscale* one level below, and the *macroscale* one level above, are described only as needed to define the coarse-graining maps of variables between adjacent levels. Important properties such as bidirectionality of escapes from metastable fixed point in the stationary distribution, which the large-deviation analysis in the mesoscale supplies as properties of elementary transitions in the macroscale, will be assumed self-consistently as inputs from the microscale to the mesoscale.

Within the description at a single level, the discrete population states will be termed *microstates*, as is standard in (both classical and stochastic) thermodynamics. Microstates are different in kind from *macrostates*, which correspond to a sub-class of distributions (defined below), and from fixed points of the dynamical system. There is no unique recipe for coarse-graining descriptions to reduce dimensionality in a multi-scale system, because the diversity of stochastic processes is vast. Here, to obtain a manageable terminology and class of models, we limit to cases in which the dynamical-system fixed points at one level can be put in correspondence with the microstates at the next higher level. Table 1 shows the terms that arise within, and correspondences between, levels.

### 2.2. Models Based on Population Processes

The following elements furnish a description of a system:

The multiscale distribution:

The central object of study is any probability distribution ρ, defined down to the smallest scale in the model, and the natural coarse-grainings of ρ produced by the dynamics. To simplify notation, we will write ρ for the general distribution, across all levels, and let the indexing of ρ indicate which level is being used in a given computation.

Fast relaxation to fixed points at the microscale:

The counterpart, in our analysis of the mesoscale, to Prigogine’s [13,14] assumption of local equilibrium in a bath, is fast relaxation of the distribution on the microscale, to a distribution with modes around the fixed points. (For an example allowing relaxation to local stationary non-equilibrium, in the same spirit as the approach here, see the averaging over fast degrees of freedom in [55].) In the mesoscale these fixed points become the elementary population states indexed n, and the coarse-grained probability distribution is denoted $\rho_{n}$. If a population consists of individuals of *types* indexed $p\in \left\{1,\ldots ,P\right\}$, then $n\equiv \left[n_{p}\right]$ is a vector in which the non-negative integer coefficient $n_{p}$ counts the number of individuals of type *p*.

Elementary transitions in the mesoscale:

First-passages occurring in the (implicit) large-deviations theory, between fixed points $n^{\prime }$ and n at the microscale, appear in the mesoscale as elementary transitions $n^{\prime }\to n$. In Figure 1, the elementary states are grid points and elementary transitions occur along lines in the grid in the middle layer. We assume as input to the mesoscale the usual condition of weak reversibility [70], meaning that if an elementary transition $n^{\prime }\to n$ occurs with nonzero rate, then the transition $n\to n^{\prime }$ also occurs with nonzero rate. Weak reversibility is a property we will derive for first passages within the mesoscale, motivating its adoption at the lower level.

Thermalization in the microscale:

The elementary transitions $n^{\prime }↔n$ are separated by typical intervals exponentially longer in some scale factor than the typical time in which a single transition completes. (In the large-deviation theory, they are *instantons*.) That timescale separation defines thermalization at the microscale, and makes microscale fluctuations conditionally independent of each other, given the index n of the basin in which they occur. Thermalization also decouples components $n_{p}$ in the mesoscale at different *p* except through the allowed elementary state transitions.

A System/Environment partition within the mesoscale:

Generally, in addition to considering the thermalized microscale a part of the “environment” in which mesoscale stochastic events take place, we will choose some partition of the type-indices *p* to distinguish one subset, called the *system* (*s*), from one or more other subsets that also form part of the *environment* (*e*). Unlike the thermalized microscale, the environment-part in the mesoscale is slow and explicitly stochastic, like the system. The vector n indexes a tensor product space s⊗e, so we write $n\equiv \left(n_{s},n_{e}\right)$.

The notation here for nested population processes lends itself directly to examples such as large chemical reaction networks, in which a subset of species and reactions are regarded as the system *s* and the remainder serve as an environment *e* of chemostats, or to Darwinian populations in which a subset *s* of focal species evolve under frequency-dependent selection with background populations *e* treated only in aggregate. The reader is asked to imagine as well a variety of heterogeneous multi-level systems that could be handled in similar fashion with suitable notations for various case-specific state spaces. Examples of current interest include the use of active media such as chemotactic bacteria to drive mesomechanical loads [82,83]. The load is *s*, undergoing biased Brownian motion, while the active medium is *e*, which must be modeled dynamically on the same timescale as the load. Active media with biological components are also most-naturally modeled with completely irreversible elementary events, and provide a large part of our motivation to demonstrate how to handle such limits formally in Section 6.1.3.

Marginal and conditional distributions in system and environment:

On s⊗e, $\rho_{n}$ is a joint distribution. A marginal distribution $\rho^{s}$ for the system is defined by $\rho_{n_{s}}^{s}\equiv \sum_{n|n_{s}}\rho_{n}$, where $n|n_{s}$ fixes the *s* component of n in the sum. From the joint and the marginal a conditional distribution $\rho^{e|s}$ at each $n_{s}$ is given by $\rho_{n=\left(n_{s},n_{e}\right)}\equiv \rho_{n_{s}}^{s}\rho_{n}^{e|s}$. The components of $\rho^{e|s}$ can fill the role often given to chemostats in open-system models of CRNs. Here we keep them as explicit distributions, potentially having dynamics that can respond to changes in *s*.

Notations involving pairs of indices:

Several different sums over the pairs of indices associated with state transitions appear in the following derivations. To make equations easier to read, the following notations are used throughout:

- $\langle n,n^{\prime }\rangle$ is an unordered pair of indices.
- $\sum_{n}\sum_{n^{\prime }\neq n}$ counts every pair in both orders.
  $\sum_{\langle n,n^{\prime }\rangle }$ counts every unordered pair once.
- Therefore for any function $f\;\left(n,n^{\prime }\right)$, $\sum_{n}\sum_{n^{\prime }\neq n}f\;\left(n,n^{\prime }\right)=\sum_{\langle n,n^{\prime }\rangle }\left[f\;\left(n,n^{\prime }\right)+f\;\left(n^{\prime },n\right)\right]$.
- $\sum_{n|n_{s}}$ is a sum on the $n_{e}$ component of $n=\left(n_{s},n_{e}\right)$.
- $\sum_{\langle n,n^{\prime }\rangle |n_{s}}$ counts all unordered pairs $\langle n,n^{\prime }\rangle$ with common *s*-component $n_{s}$.

### 2.3. Stochastic Description within the Mesoscale

The coarse-grained distribution $\rho_{n}$ at the mesoscale evolves in time under a master equation

(1) $$\begin{array}{cc} {\dot{\rho }}_{n}=\sum_{n^{\prime }}T_{nn^{\prime }}\rho_{n^{\prime }} & =\sum_{n^{\prime }\neq n}\left(w_{nn^{\prime }}\rho_{n^{\prime }}-w_{n^{\prime }n}\rho_{n}\right). \end{array}$$

Here and below, $\left(\dot{}\right)$ indicates the time derivative. The generator T is a stochastic matrix on the left, which we write $1^{T}T=0$, where $1^{T}$ is the row-vector on index n corresponding to the uniform (unnormalized) measure. $w_{nn^{\prime }}$ (following a standard notation [70]) is the component of T giving the transition rate from state $n^{\prime }$ to state n.

For all of what follows, it will be necessary to restrict to systems that possess a stationary, normalizable distribution denoted $\underline{\rho }$, satisfying $T\underline{\rho }=0$. The stationary distribution will take the place of conservation laws as the basis for the definition of macrostates. Moreover, if $\underline{\rho }$ is everywhere continuous, and the number of distinct events generating transitions in the mesoscale (in a sense made precise below) is finite, first passages between fixed points corresponding to modes of $\underline{\rho }$ will occur at rates satisfying a condition of detailed balance. The joint, marginal, and conditional stationary distributions are denoted ${\underline{\rho }}_{n=\left(n_{s},n_{e}\right)}\equiv {\underline{\rho }}_{n_{s}}^{s}{\underline{\rho }}_{n}^{e|s}$.

The marginal stochastic process on *s*:

The system-marginal distribution $\rho^{s}$ evolves under a master equation ${\dot{\rho }}^{s}=T^{s}\;\left(\rho^{e|s}\right)\rho^{s}$, for which the transition matrix $T^{s}$ has components that are functions of the instantaneous environmental distribution, given by

(2) $$\begin{array}{cc} w_{n_{s}n_{s}^{\prime }}^{s}\;\left(\rho^{e|s}\right) & \equiv \sum_{n|n_{s}}\sum_{n^{\prime }|n_{s}^{\prime }}w_{nn^{\prime }}\rho_{n^{\prime }}^{e|s}. \end{array}$$

The only assumption on the separation of timescales underlying Equation (2) is that the same treatment to eliminate fast variables has been used across the system *s* and environment *e* that remain in the mesoscale. In practice this is not restrictive other than to assume that timescale separations are sparse enough to leave well-defined scales between them to describe with fixed collections of stochastic variables; for a given level of description we eliminate all faster variables than those left explicit. As noted in the summary Section 1.1, the separation need not be asymptotic or absolute: as long as eikonal methods identify fixed points in the microscale that can serve as elementary states in the mesoscale, and as long as an instanton method (with or without perturbative corrections) exists to compute effective rate constants (see Section 3.2.2, Section 4.2.1 and Section 4.2.2 later in the paper), the mesoscale projection has a well-defined construction by elimination of faster degrees of freedom.

Time-independent overall stationary distribution as a reference:

To maintain a clean abstraction in which all dynamics is kept explicit within the written distribution ρ, the parameters defining the multiscale stochastic process are assumed time-homogeneous. This assumption makes possible the definition of a Lyapunov function for the entire multi-level system distribution, and is tantamount to formally closing the system description. (Equivalently, the stochastic process abstraction is the only one assumed; any mechanical control parameters or other degrees of freedom are to be internal degrees of freedom simply possessing restricted distributions.) Since fast degrees of freedom at the microscale are assumed to relax to unique fixed points except for rare transitions that are left as the explicit events in the mesoscale, time-homogeneity of the overall description implies time-homogeneity of the effective coefficients in the mesoscale transition matrix T. Residual mesoscale dynamics is then carried exclusively in the distributions $\rho^{s}$ and $\rho^{e|s}$.

Detailed balance propagating up from the microscale:

Finally we assume, propagating up from the microscale, a condition of detailed balance that we will prove as a property of first-passages in the mesoscale in Section 4.2.2, and then apply recursively:

(3) $$\begin{array}{cc} w_{nn^{\prime }}{\underline{\rho }}_{n^{\prime }} & =w_{n^{\prime }n}{\underline{\rho }}_{n}. \end{array}$$

Note that, as was a central theme in [55], condition (3) is not an assumption of microscopic reversibility in whatever faster stochastic process is operating below in the microscale. To understand why, using constructions that will be carried out explicitly within the mesoscale, see that even with rate constants satisfying Equation (3), the system-marginal transition rates (2) need not satisfy a condition of detailed balance. Indeed we will want to be able to work in limits for the environment’s conditional distributions $\rho^{e|s}$ in which some transitions can be made completely irreversible: that is $w_{n_{s}n_{s}^{\prime }}^{s}\neq 0$ but $w_{n_{s}^{\prime }n_{s}}^{s}=0$. Even from such irreversible dynamics among microstates, first-passage rates with detailed balance in the stationary distribution will result, and it is that property that is assumed in Equation (3).

From these assumptions on T and $\underline{\rho }$, it follows that the relative entropy of any distribution ρ from the stationary distribution $\underline{\rho }$ is non-decreasing,

(4) $$\begin{array}{cc} -\dot{D}\;\left(\rho \|\underline{\rho }\right) & =\sum_{n}\sum_{n^{\prime }\neq n}\log\left(\frac{{\left(\rho /\underline{\rho }\right)}_{n^{\prime }}}{{\left(\rho /\underline{\rho }\right)}_{n}}\right)w_{nn^{\prime }}\rho_{n^{\prime }}\\ \; & =\sum_{n}\sum_{n^{\prime }\neq n}\log\left(\frac{w_{nn^{\prime }}\rho_{n^{\prime }}}{w_{n^{\prime }n}\rho_{n}}\right)w_{nn^{\prime }}\rho_{n^{\prime }}\\ \; & =\sum_{\langle n,n^{\prime }\rangle }\log\left(\frac{w_{nn^{\prime }}\rho_{n^{\prime }}}{w_{n^{\prime }n}\rho_{n}}\right)\left(w_{nn^{\prime }}\rho_{n^{\prime }}-w_{n^{\prime }n}\rho_{n}\right)\geq 0. \end{array}$$

The result (4) appears as an integral fluctuation theorem in all stochastic treatments of the total entropy change [21,47,48,49,54,55,57]; as a single-time result it is elementary for systems with detailed balance, because each term in the third line of Equation (4) is individually non-negative. We use *relative entropy* to refer to minus the Kullback–Leibler divergence of ρ from $\underline{\rho }$ [66], to follow the usual sign convention for a non-decreasing entropy.

### 2.4. System-Environment Decompositions of the Entropy Change

The exchange of heat for work is central to classical thermodynamics because energy conservation is a constraint on joint configurations across sub-systems, either multiple thermal systems in contact or a mechanical subsystem having only deterministic variables, some of which set the boundary conditions on thermal subsystems that also host fluctuations. Only the state-function entropy, however, is a “function” of energy in any sense, so the only notion of a limiting partition of irreversible effects between subsystems derivable from energy conservation is the one defined by adiabatic transformations passing through sequences of macrostates.

In more general cases, with or without conservation laws, the boundary conditions on a system are imposed only through the elements of the marginal transition matrix $T^{s}$. The problem remains, of understanding how one subsystem can limit entropy change in another through a boundary, but it is no longer organized with reference to adiabatic transformations.

We wish to understand what constitutes a thermodynamically natural decomposition of the mesoscale process into a system and an environment. A widely-adopted decomposition [5,70] for systems with energy conservation separates a Shannon entropy of $\rho_{n}^{s}$ from heat generation associated with terms $-\log w_{n_{s}n_{s}^{\prime }}^{s}$ by the local equilibrium assumption for the bath. (The decomposition is the same one used to define an energy cost of computation [2,3] by constructing logical states through the analogues to heat engines [1,61].) We begin by writing down this information/heat decomposition, and arguing that it is not the natural partition with respect to irreversibility. An equivalent argument, not framed as one regarding inherent system/environment identities, but rather as making fullest use of available fluctuation theorems, is made in [21].

Entropy relative to the stationary state rather than Shannon entropy:

The following will differ from the usual construction in replacing Shannon entropy with a suitable relative entropy, without changing the essence of the decomposition. As noted in [49], the natural entropy for a stochastic process will generally be a relative entropy for which a measure must be specified. Two arguments may be given for this claim: It would be clear, for a system with a continuous state space in which $\rho_{n}$ would become a density, that the logarithm of a dimensional quantity is undefined. Hence some reference measure is always implicitly assumed. A uniform measure is not a coordinate-invariant concept, and a measure that is uniform in one coordinate system makes those coordinates part of the system specification. (The same argument is made in [84] with respect to Bayesian statistics, and as a critique of arguments in “objective Bayesianism” that one can evade the need to choose.) Since discrete processes are often used as approximations to continuum limits, the same concerns apply. The more general lesson is that a logarithmic entropy unit is always given meaning with respect to some measure. Only for systems such as symbol strings, for which a combinatorial measure on integers is the natural measure, is Shannon entropy the corresponding natural entropy. For other cases, such as CRNs, the natural entropy is relative entropy referenced to the Gibbs equilibrium [61], and its change gives the dissipation of chemical work. For the processes described here, the counterpart to Shannon entropy that solves these consistency requirements, but does not yet address the question of naturalness, is the relative entropy referenced to the steady-state marginal ${\underline{\rho }}^{s}$. Its time derivative is given by

(5) $$\begin{array}{cc} -\dot{D}\;\left(\rho^{s}\|{\underline{\rho }}^{s}\right) & =\sum_{\langle n_{s},n_{s}^{\prime }\rangle }\log\left(\frac{{\left(\rho /\underline{\rho }\right)}_{n_{s}^{\prime }}^{s}}{{\left(\rho /\underline{\rho }\right)}_{n_{s}}^{s}}\right)\left(w_{n_{s}n_{s}^{\prime }}^{s}\rho_{n_{s}^{\prime }}^{s}-w_{n_{s}^{\prime }n_{s}}^{s}\rho_{n_{s}}^{s}\right). \end{array}$$

The quantity (5) need not be either positive or negative in general.

A second term that separates out of the change in total relative entropy (4) comes from changes in environmental states through events that do not result in net change of the system state. (Note $w_{nn^{\prime }}$ may depend on the system index-component $n_{s}$ shared by both n and $n^{\prime }$, so these rates can depend on system state. Catalysis acts through such dependencies.) The relative entropy of the conditional distribution $\rho^{e|s}$ at a particular index $n_{s}$ from its stationary reference ${\underline{\rho }}^{e|s}$ has time derivative

(6) $$\begin{array}{cc} -{\dot{D}}_{n_{s}}^{e|s}\;\left(\rho^{e|s}\|{\underline{\rho }}^{e|s}\right) & =\sum_{\langle n_{s},n_{s}^{\prime }\rangle |n_{s}}\log\left(\frac{{\left(\rho /\underline{\rho }\right)}_{n^{\prime }}^{e|s}}{{\left(\rho /\underline{\rho }\right)}_{n}^{e|s}}\right)\left(w_{nn^{\prime }}\rho_{n^{\prime }}^{e|s}-w_{n^{\prime }n}\rho_{n}^{e|s}\right)\\ \; & =\sum_{\langle n_{s},n_{s}^{\prime }\rangle |n_{s}}\log\left(\frac{w_{nn^{\prime }}\rho_{n^{\prime }}^{e|s}}{w_{n^{\prime }n}\rho_{n}^{e|s}}\right)\left(w_{nn^{\prime }}\rho_{n^{\prime }}^{e|s}-w_{n^{\prime }n}\rho_{n}^{e|s}\right). \end{array}$$

Unlike the change of system relative entropy (5), Equation (6) is non-negative term-by-term, in the same way as Equation (4).

The remaining terms to complete the entropy change (4) come from joint transformations in system and environment indices $n_{s}$ and $n_{e}$, and in usual treatments have the interpretation of dissipated heats. (When more than one environment transition couples to the same system transition, there can be reasons to further partition these terms; an example is given in Section 6.1.) They are functions of the pair of indices $\left(n_{s}^{\prime },n_{s}\right)$. An average change in relative entropy of the environment, over all processes that couple to a given system state-change, is

(7) $$\begin{array}{cc} \sigma_{n_{s}n_{s}^{\prime }}\;\left(\rho^{e|s}\right) & \equiv \frac{1}{w_{n_{s}n_{s}^{\prime }}^{s}}\sum_{n|n_{s}}\sum_{n^{\prime }|n_{s}^{\prime }}\log\left(\frac{{\left(\rho /\underline{\rho }\right)}_{n^{\prime }}^{e|s}}{{\left(\rho /\underline{\rho }\right)}_{n}^{e|s}}\right)w_{nn^{\prime }}\rho_{n^{\prime }}^{e|s}. \end{array}$$

Note that if we had wished to use the un-referenced Shannon entropy $-\sum_{n_{s}}\rho_{n_{s}}^{s}\log\rho_{n_{s}}^{s}$ in place of the relative entropy (5)—for instance, in an application to digital computing—we could shift the measures ${\underline{\rho }}^{s}$ to the dissipation term to produce what is normally considered the “environmental” heat dissipation, given by

(8) $$\begin{array}{cc} \sigma_{n_{s}n_{s}^{\prime }}^{\mathrm{env}}w_{n_{s}n_{s}^{\prime }}^{s} & \equiv \left[\sigma_{n_{s}n_{s}^{\prime }}-\log\left(\frac{{\underline{\rho }}_{n_{s}^{\prime }}^{s}}{{\underline{\rho }}_{n_{s}}^{s}}\right)\right]w_{n_{s}n_{s}^{\prime }}^{s}\\ \; & =\sum_{n|n_{s}}\sum_{n^{\prime }|n_{s}^{\prime }}\log\left(\frac{w_{nn^{\prime }}\rho_{n^{\prime }}^{e|s}}{w_{n^{\prime }n}\rho_{n}^{e|s}}\right)w_{nn^{\prime }}\rho_{n^{\prime }}^{e|s}. \end{array}$$

The quantity (8) is regarded as a property of the environment (both slow variables and the thermal bath) because it is a function only of the transition rates $w_{n^{\prime }n}$ and of the marginal distributions $\rho^{e|s}$.)

#### 2.4.1. The Information/Heat Decomposition of Total Relative-Entropy Change

Equation (4) is decomposed in terms of the quantities in Equations (5)–(7) as

(9) $$\begin{array}{cc} -\dot{D}\;\left(\rho \|\underline{\rho }\right) & =-\dot{D}\;\left(\rho^{s}\|{\underline{\rho }}^{s}\right)+\sum_{n_{s}}\sum_{n_{s}^{\prime }\neq n_{s}}\sigma_{n_{s}n_{s}^{\prime }}\;w_{n_{s}n_{s}^{\prime }}^{s}\rho_{n_{s}^{\prime }}^{s}-\sum_{n_{s}}\rho_{n_{s}}^{s}{\dot{D}}_{n_{s}}^{e|s}\;\left(\rho^{e|s}\|{\underline{\rho }}^{e|s}\right). \end{array}$$

The total is non-negative and the third summand is independently non-negative, as already mentioned. The sum of the first two summands is also non-negative, a result that can be proved as a fluctuation theorem for what is normally called “total entropy change” [21]. (More detailed proofs for a decomposition of the same sum will be given below.)

Here we encounter the first property that makes a decomposition of a thermal system “natural”. The term in ${\dot{D}}_{n_{s}}^{e|s}\;\left(\rho^{e|s}\|{\underline{\rho }}^{e|s}\right)$ is not generally considered, and it does not need to be considered, because thermal relaxation at the microscale makes transitions in $n_{e}$ at fixed $n_{s}$ conditionally independent of transitions that change $n_{s}$. Total relative entropy changes as a sum of two independently non-decreasing contributions.

The first and second lines in Equation (9) are not likewise independently non-negative. Negative values of the second or first line, respectively, describe phenomena such as randomization-driven endothermic reactions, or heat-driven information generators. To the extent that they do not use thermalization in the microscale to make the system and environment conditionally independent, as the third summand in Equation (9) is independent, we say they do not provide a natural system/environment decomposition.

#### 2.4.2. Relative Entropy Referencing the System Steady State at Instantaneous Parameters

Remarkably, a natural division does exist, based on the *housekeeping heat* introduced in a phenomenological treatment by Oono and Paniconi [50], for which a fluctuation theorem was subsequently derived by Hatano and Sasa [51] followed by Speck and Seifert [52]. (In related proofs later in Section 5.2.2, we will more nearly follow the treatment by Harris and Schutz [53].)

The decomposition uses the solution ${\bar{\rho }}^{s}$ to $T^{s}{\bar{\rho }}^{s}=0$, which would be the stationary marginal distribution for the system *s* at the instantaneous value of $\rho^{e|s}$. As for the whole-system stationary distribution $\underline{\rho }$, we restrict to cases in which ${\bar{\rho }}^{s}$ exists and is normalizable. (There are multiple ways in which a normalizable marginal distribution ${\bar{\rho }}^{s}$ does not restrict normalizability elsewhere in the description. Environment distributions $\rho^{e|s}$ may be taken to large-system limits to model chemostats, for which a normalizable distribution on an unbounded state space may have asymptotically unbounded entropy and also have the same effect in *s* as a non-normalizable distribution in a covering space that counts cycles traversed within *s* [49]. A more serious restriction is faced if chemostats permit no normalizable distribution within the system, as might occur quite naturally in polymerization models admitting indefinite growth. In such cases, other methods of analysis must be used [85].)

Treating ${\bar{\rho }}^{s}$ as fixed and considering only the dynamics of $\rho^{s}$ with ${\bar{\rho }}^{s}$ as a reference, we may consider a time derivative $\dot{D}\;\left(\rho^{s}\|{\bar{\rho }}^{s}\right)$ in place of Equation (5). Note that the rates $w_{n_{s}n_{s}^{\prime }}^{s}$ in the transition matrix $T^{s}$ no longer need satisfy any simplified balance condition in relation to ${\bar{\rho }}^{s}$, such as detailed balance. Non-negativity of $-\dot{D}\;\left(\rho^{s}\|{\bar{\rho }}^{s}\right)$ was proved by Schnakenberg [76] by an argument that applies to discrete population processes with normalized stationary distributions of the kind assumed here. We will derive a slightly more detailed decomposition proving this result for the case of CRNs, in a later section.

The dissipation term that complements the change in $\dot{D}\;\left(\rho^{s}\|{\bar{\rho }}^{s}\right)$ is obtained by shifting the “environmental” entropy change (8) by $\log{\bar{\rho }}^{s}$ to obtain the housekeeping heat

(10) $$\begin{array}{cc} \sigma_{n_{s}n_{s}^{\prime }}^{\mathrm{HK}}w_{n_{s}n_{s}^{\prime }}^{s} & \equiv \left[\sigma_{n_{s}n_{s}^{\prime }}^{\mathrm{env}}+\log\left(\frac{{\bar{\rho }}_{n_{s}^{\prime }}^{s}}{{\bar{\rho }}_{n_{s}}^{s}}\right)\right]w_{n_{s}n_{s}^{\prime }}^{s}\\ \; & =\sum_{n|n_{s}}\sum_{n^{\prime }|n_{s}^{\prime }}\log\left(\frac{{\bar{\rho }}_{n_{s}^{\prime }}^{s}\rho_{n^{\prime }}^{e|s}/{\underline{\rho }}_{n^{\prime }}}{{\bar{\rho }}_{n_{s}}^{s}\rho_{n}^{e|s}/{\underline{\rho }}_{n}}\right)w_{nn^{\prime }}\rho_{n^{\prime }}^{e|s}. \end{array}$$

(These terms are used because this is how they are known. No energy interpretation is assumed here, so a better term would be “housekeeping entropy rate”). Non-negativity of Equation (10) is implied by a fluctuation theorem [52], and a time-local proof with interesting further structure for CRNs will be given below.

The total change in relative entropy (4) is then the sum

(11) $$\begin{array}{cc} -\dot{D}\;\left(\rho \|\underline{\rho }\right) & =-\dot{D}\;\left(\rho^{s}\|{\bar{\rho }}^{s}\right)+\sum_{n_{s}}\sum_{n_{s}^{\prime }\neq n_{s}}\sigma_{n_{s}n_{s}^{\prime }}^{\mathrm{HK}}\;w_{n_{s}n_{s}^{\prime }}^{s}\rho_{n_{s}^{\prime }}^{s}-\sum_{n_{s}}\rho_{n_{s}}^{s}{\dot{D}}_{n_{s}}^{e|s}\;\left(\rho^{e|s}\|{\underline{\rho }}^{e|s}\right). \end{array}$$

Each summand in Equation (11) is now independently non-negative. The first measures a gain of entropy within the system *s*, conditionally independent of changes in the environment given the marginal transition matrix $T^{s}$. The third measures a gain of entropy in the environment *e* independent of any changes in the system at all. The second, housekeeping entropy rate, measures a change of entropy in the environment that is conditionally independent of changes of entropy within the system, given $T^{s}$ as represented in ${\bar{\rho }}^{s}$. Any of the terms may be changed, holding the conditioning data ${\bar{\rho }}^{s}$ or $n_{s}$, or omitted, without changing the limits for the others, as observed in [48,55]. They respect the conditional independence created by thermalization in the microscale, and by that criterion constitute a natural decomposition of the system.

#### 2.4.3. Intrinsic and Extrinsic Thermodynamics

We take $T^{s}$ and $D\;\left(\rho^{s}\|{\bar{\rho }}^{s}\right)$ as a specification of the *intrinsic thermodynamics* of the system *s*, analogous to the role of intrinsic curvature of a manifold in differential geometry. The vector (indexed by pairs of system indices) of housekeeping entropy differentials, $\left\{\sigma_{n_{s}n_{s}^{\prime }}^{\mathrm{HK}}\right\}$, correspondingly defines the way *s* is thermodynamically embedded in the mesoscale system, analogous to the role of components of an embedding curvature for a sub-manifold within larger manifold. In differential geometry, the embedding curvature is termed extrinsic curvature, and by analogy we refer to $\left\{\sigma_{n_{s}n_{s}^{\prime }}^{\mathrm{HK}}\right\}$ as defining an *extrinsic thermodynamics* for *s* within the larger s⊗e mesoscale world. The rates we separate as intrinsic and extrinsic correspond respectively to the “non-adiabatic” and “adiabatic” entropy production designations in [21,50].

#### 2.4.4. System Hartley Information as a Temporal Connection

The natural decomposition (11) differs from the information/heat decomposition (9) in what is regarded as inherent to the system versus the environment, as mentioned briefly above in Section 1.1. The attribution of Shannon entropy as a “system” property follows from the fact that it involves only $\rho_{n_{s}}^{s}$, and its change counts only actual transitions $n_{s}^{\prime }\to n_{s}$ with rate $w_{n_{s}n_{s}^{\prime }}^{s}$. Likewise, the “environment” heat (8) is a function only of the actual distribution $\rho^{e|s}$ and the realized currents.

Those events that occur within *s* or *e*, however, fail to capture the additional features of $T^{s}$: that specific transitions are coupled as a system, and that they have the dependence on $\rho^{e|s}$ of Equation (2). The stationary distribution ${\bar{\rho }}^{s}$ is the function of $T^{s}$ reflecting its status as a system. In the degenerate case where irreversibility is removed and the system obeys detailed balance, the stationary distribution ${\bar{\rho }}^{s}$ reduces to the marginal Gibbs measure on the system degrees of freedom, but as part of the relative entropy it is treated as part of the system description, in contrast to the mechanical view where it is appears as an intensive boundary condition associated with the environment.

The differences between the three entropy-change terms (7,8,10) are differences of the Hartley informations [46], respectively for ${\underline{\rho }}^{s}$ or ${\bar{\rho }}^{s}$. In the natural decomposition (11), they are not acted upon by the time derivative, but rather define the tangent plane of zero change for logρ terms that are acted upon by the transition matrix, and resemble connection coefficients specifying parallel transport in differential geometry.

## 3. Hamilton–Jacobi Theory for Large Deviations

The central concepts in thermodynamics are that of the macrostate, and of the entropy as a state function from which properties of macrostates and constraints on their transformations are derived. In classical thermodynamics [39,86], macrostates are introduced in association with average values of conserved quantities (e.g., energy, particle numbers). Conservation laws provide the essential element of conditioning in probability. Energy conservation alone, in the context of detailed balance that results from microscopic reversibility under conditions of time-translation symmetry, enables specification of invariant marginal distributions conditionally independent of, and without a need to specify, the entire state space. The generality of energy conservation also makes it a robust property on which to condition.

Here, we wish to separate the construction that defines a macrostate from the properties that make one or another class of macrostates dynamically robust in a given system. The property of conditioning will be provided not by conservation, but by reference to stationary distributions and a the tilting procedure by which generating functions are defined from these. The defining construction can be quite general, but it must in all cases create a dimensional reduction by an indefinite (or in the limit infinite) factor, from the dimensionality of the microstate space that is definite but arbitrarily large, to the dimensionality of a space of macrostate variables that is fixed and independent of the dimensionality of microstates. Only dimensional reductions of this kind are compatible with the large-deviation definition of macroworlds as worlds in which structure can be characterized asymptotically separate from scale [43]. Robustness can then be characterized separately within the large-deviation analysis in terms of closure approximations or spectra of relaxation times for various classes of macrostates.

Dimensional reduction by an indefinite degree is achieved by associating macrostates with particular classes of distributions over microstates: namely, those distributions produced in exponential families to define generating functions. The coordinates in the tilting weights that define the family are independent of the dimension of the microstate space for a given family and become the intensive state variables (see [41]). They are related by Legendre transform to deviations that are the dual extensive state variables. Relative entropies such as $-D\;\left(\rho \|\underline{\rho }\right)$, defined as functionals on arbitrary distributions, and dual under Legendre transform to suitable cumulant-generating functions, become *state functions* when restricted to the distributions for macrostates. The extensive state variables are their arguments and the intensive state variables their gradients. Legendre duality leads to a system of Hamiltonian equations [40] for time evolution of macrostate variables, and from these the large-deviation scaling behavior, timescale structure, and moment closure or other properties of the chosen system of macrostates are derived.

In the treatment of Section 2, the relative entropy increased deterministically without reference to any particular level of system timescales, or the size-scale factors associated with various levels. As such, it fulfilled the Lyapunov role of (minus) the entropy, but not the large-deviation role that is the other defining characteristic of entropy [43,44]. The selection of a subclass of distributions as macrostates introduces level-dependence, scale-dependence, and the large-deviation role of entropy, and lets us construct the relation between the Lyapunov and large-deviation roles of entropy for macroworlds, which are generally distinct. As a quantity capable of fluctuations, the macrostate entropy can decrease along subsets of classical trajectories; these fluctuations are the objects of study in stochastic thermodynamics. The Hamiltonian dynamical system is a particularly clarifying representation for the way it separates relaxation and fluctuation trajectories for macrostates into distinct sub-manifolds. In distinguishing the unconditional versus conditional nature of the two kinds of histories, it shows how these macro-fluctuations are not “violations” of the 2nd law, but rather a partitioning of the elementary events through which the only properly formulated 2nd law is realized. (See a brief discussion making essentially this point in Section 1.2 of [5]. Seifert refers to the 2nd law as characterizing “mean entropy production”, in keeping with other interpretations of entropies such as the Hartley information in terms of heat. The characterization adopted here is more categorical: the Hartley function and its mean, Shannon information, are not quantities with the same interpretation; likewise, the entropy change (11) is the only entropy relative to the boundary conditions in T that is the object of a well-formulated 2nd law. The “entropy productions” resulting from the Prigogine local-equilibrium assumption are conditional entropies for macrostates defined through various large-deviation functions, shown explicitly below.)

### 3.1. Generating Functions, Liouville Equation, and the Hamilton–Jacobi Construction for Saddle Points

#### 3.1.1. Relation of the Liouville Operator to the Cumulant-Generating Function

The *P*-dimensional Laplace transform of a distribution $\rho_{n}$ on discrete population states gives the moment-generating function (MGF) for the species-number counts ${\left\{n_{p}\right\}}_{p\in 1,\ldots ,P}$. For time-dependent problems, it is convenient to work in the formal Doi operator algebra for generating functions, in which a vector of raising operators $a^{†}\equiv \left[a_{p}^{†}\right]$ are the arguments of the MGF, and a conjugate vector of lowering operators $a\equiv \left[a_{p}\right]$ are the formal counterparts to $\partial /\partial a^{†}$. MGFs are written as vectors $\left|\rho \right)\equiv \sum_{n}\rho_{n}{\left(a^{†}\right)}^{n}\left|0\right)\equiv \sum_{n}\rho_{n}\left|n\right)$ in a Hilbert space built upon a ground state $\left|0\right)$, and the commutation relations of the raising and lowering operators acting in the Hilbert space are $\left[a_{p},a_{q}^{†}\right]=\delta_{pq}$.

The basis vectors corresponding to specific population states are denoted $\left|n\right)$. They are eigenvectors of the number operators $a_{p}^{†}a_{p}$ with eigenvalues $n_{p}$:

(12) $$\begin{array}{cc} a_{p}^{†}a_{p}\left|n\right) & =n_{p}\left|n\right). \end{array}$$

Through by-now-standard constructions [67], the master equation $\dot{\rho }=T\rho$ is converted to a Liouville equation for time evolution of the MGF,

(13) $$\begin{array}{cc} \frac{d}{dt}\left|\rho \right) & =-L\;\left(a^{†},a\;\right)\left|\rho \right), \end{array}$$

in which the Liouville operator $L\;\left(a^{†},a\;\right)$ is derived from the elements of the matrix −T.

To define exponential families and a cumulant-generating function (CGF), it is convenient to work with the Laplace transform with an argument that is a vector $z\equiv \left[z_{p}\right]$ of complex coefficients. The corresponding CGF, $-\Gamma \;\left(\log z\right)$, for which the natural argument is logz, is constructed in the Doi Hilbert space as the inner product with a variant on the Glauber norm,

(14) $$\begin{array}{cc} e^{-\Gamma \left(\log z\right)} & =\left(0\right|e^{za}\left|\rho \right). \end{array}$$

*z* will be called the *tilt* of the exponential family, corresponding to its usage in importance sampling [87]. (We adopt the sign for Γ corresponding to the free energy in thermodynamics. Other standard notations, such as ψ for the CGF [41], are unavailable because they collide with notations used in the representation of CRNs below.)

An important quantity will be the vector of expectations of the number operators in the tilted distribution

(15) $$\begin{array}{cc} {n_{\rho }}_{p}\;\left(z\right) & \equiv \frac{\left(0\right|e^{za}a_{p}^{†}a_{p}\left|\rho \right)}{\left(0\right|e^{za}\left|\rho \right)}. \end{array}$$

Time evolution of the CGF follows from Equation (13), as

(16) $$\begin{array}{cc} \frac{\partial }{\partial t}e^{-\Gamma \left(\log z\right)} & =-\left(0\right|e^{za}L\;\left(a^{†},a\;\right)\left|\rho \right). \end{array}$$

Under the saddle-point or leading-exponential approximation that defines the large-deviation limit (developed further below), the expectation of L in Equation (16) is replaced by the same function at classical arguments

(17) $$\begin{array}{cc} -\frac{\partial }{\partial t}\Gamma \;\left(\log z\right) & =-L\;\left(z,n_{\rho }\;\left(z\right)/z\right). \end{array}$$

A systematic though indirect way to derive Equation (17) is by means of coherent-state expansions and 2-field functional integral representations of the generating function, reviewed didactically with a simple example in [67]. The flavor of the construction is easy to understand, however. The operator $e^{za}$ acts, through the commutation relations $\left[a_{p},a_{q}^{†}\right]=\delta_{pq}$, as a shift operator taking $a^{†}\to a^{†}+z$ on all instances of $a^{†}$ in $L\;\left(a^{†},a\;\right)$. The left null state $\left(0\right|$ in the inner product is annihilated by all $a^{†}$, so in the ordering of L called *normal order* that places all $a^{†}$ operators to the left of all *a* operators, the only remaining argument is the number *z*. The substitution of *a* by $n_{\rho }\;\left(z\right)/z$ relies on the replacement of all instances of $a_{p}^{†}a_{p}$ by factors of $n_{p}$, followed by the mean-field approximation—another name for the saddle-point approximation—in which higher-order correlations are approximated by equivalent powers of the mean. The factor *a* in these number expansions differs from the number operator $a^{†}a$ by additional factors of $a^{†}$ replaced by *z* as explained above. The saddle-point evaluation of *a* with the coherent-state parameter that is its eigenvalue is more direct in the functional-integral construction [67], where coherent-state and number fields are related through a canonical transformation [88] in the field variables of integration.

From coherent-state to number-potential coordinates:

With normal ordering of operators in L (all lowering operators to the right of any raising operator), *z* is the exact value assigned to $a^{†}$ in the expectation (16), and only the value $n_{\rho }\;\left(z\right)/z$ for *a* depends on saddle-point approximations. In special cases, where $\left|\rho \right)$ are eigenstates of *a* known as coherent states, the assignment to *a* is also exact. Therefore, the arguments of L in Equation (17) are called *coherent-state coordinates*.

However, logz, which we henceforth denote by $\theta \equiv \left[\theta_{p}\right]$, is the affine coordinate system in which the CGF is locally convex, and it will be preferable to work in coordinates $\left(\theta ,n_{\rho }\right)$, which we term *number-potential* coordinates, because for applications in chemistry, θ has the dimensions of a chemical potential. (θ also provides the affine coordinate system in the exponential family, which defines contravariant coordinates in information geometry [41].) We abbreviate exponentials and other functions acting component-wise on vectors as $z\equiv e^{\theta }$, and simply assign $n_{\rho }\;\left(\theta \right)\equiv n_{\rho }\;\left(z\right)$. Likewise, L is defined in either coordinate system by mapping its arguments: $L^{\left(N-P\right)}\;\left(\theta ,n\right)\equiv L^{\left(\mathrm{CS}\right)}\;\left(z,n\;\left(z\right)/z\right)$.

#### 3.1.2. Legendre Transform of the CGF

To establish notation and methods, consider first distributions $\rho_{n}$ that are convex with an interior maximum in n. Then, the gradient of the CGF

(18) $$\begin{array}{c} -\frac{\partial \Gamma }{\partial \theta }=n_{\rho }\;\left(\theta \right) \end{array}$$

gives the mean (15) in the tilted distribution.

The *stochastic effective action* is the Legendre transform of −Γ, defined as

(19) $$\begin{array}{cc} S_{\mathrm{eff}}\;\left(n\right) & =\max_{\theta }\left\{\theta n+\Gamma \;\left(\theta \right)\right\}. \end{array}$$

For distributions over discrete states, to leading exponential order, $S_{\mathrm{eff}}$ is a continuously-indexed approximation to minus the log-probability: $S_{\mathrm{eff}}\;\left(n\right)∼-{\left.\log\rho_{n}\right|}_{n\approx n}$. Its gradient recovers the tilt coordinate θ,

(20) $$\begin{array}{c} \frac{\partial S_{\mathrm{eff}}}{\partial n}=\theta_{\rho }\;\left(n\right), \end{array}$$

and the CGF is obtained by inverse Legendre transform.

(21) $$\begin{array}{cc} -\Gamma \;\left(\theta \right) & =\max_{n}\left\{\theta n-S_{\mathrm{eff}}\;\left(n\right)\right\}. \end{array}$$

The time evolution of $S_{\mathrm{eff}}$ can be obtained by taking a total time derivative of Equation (19) along any trajectory, and using Equation (18) to cancel the term in $\dot{\theta }$. The partial derivative that remains, evaluated using Equation (17), gives

(22) $$\begin{array}{c} {\left.\frac{\partial S_{\mathrm{eff}}}{\partial t}\right|}_{n}=L\;\left(\theta_{\rho }\;\left(n\right),n\right). \end{array}$$

Equation (22) is of Hamilton–Jacobi form, with −L filling the role of the Hamiltonian. (The reason for this sign correspondence, which affects nothing in the derivation, will become clear below.)

Multiple modes, Legendre–Fenchel transform, and locally-defined extrema

Systems with interesting multi-level structure do not have $S_{\mathrm{eff}}$ or ρ globally convex, but rather only locally convex. For these, the Legendre–Fenchel transform takes the place of the Legendre transform in Equation (19), and if constructed with a single coordinate θ, −Γ may have discontinuous derivative.

For these one begins, rather than with the CGF, with $S_{\mathrm{eff}}$, evaluated as a line integral of Equation (20) in basins around the stationary points $n_{\rho }\;\left(0\right)$ at θ=0. Each such basin defines an invertible pair of functions $\theta_{\rho }\;\left(n|n_{\rho }\;\left(0\right)\right)$ and $n_{\rho }\;\left(\theta |n_{\rho }\;\left(0\right)\right)$. We will not be concerned with the large-deviation construction for general distributions in this paper, which is better carried out using a path integral. We return in a later section to the special case of multiple metastable fixed points in the stationary distribution, and the modes associated with the stationary distribution $\underline{\rho }$, and provide a more complete treatment.

#### 3.1.3. Hamiltonian Equations of Motion and the Action

Partial derivatives with respect to *t* and *n* commute, so the relation (20), used to evaluate ∂/∂n of Equation (22), gives the relation

(23) $$\begin{array}{cc} \frac{\partial \theta_{\rho }\;\left(n\right)}{\partial t} & =\frac{\partial L}{\partial n}. \end{array}$$

The dual construction for the time dependence of *n* from Equation (18) gives

(24) $$\begin{array}{cc} \frac{\partial n_{\rho }\;\left(\theta \right)}{\partial t} & =-\frac{\partial L}{\partial \theta }. \end{array}$$

The evolution Equations (23) and (24) describe stationary trajectories of an extended-time Lagrange–Hamilton action functional which may be written in either coherent-state or number-potential coordinates, as

(25) $$\begin{array}{cc} S & =\int dt\left\{-\left(d_{t}z\right)\left(n/z\right)+L^{\left(\mathrm{CS}\right)}\;\left(z,n/z\right)\right\}\\ \; & =\int dt\left\{-\left(d_{t}\theta \right)n+L^{\left(N-P\right)}\;\left(\theta ,n\right)\right\}. \end{array}$$

The same action functionals are arrived at somewhat more indirectly via 2-field functional integral constructions such as the Doi–Peliti method. From the form of the first term in either line, it is clear that the two sets of coordinates relate to each other through a canonical transformation [88].

Circulation-free vector field of θ for the stationary distribution

In order for $S_{\mathrm{eff}}$ to be a continuum approximation to −logρ, if ρ exists and is smooth everywhere, the vector field θ obtained from stationary trajectories of the action (25) must have zero circulation in order to be a well-defined gradient through Equation (20). To check that this is the case, consider the increment of θ under a small interval dt under Equation (23):

(26) $$\begin{array}{cc} d\theta_{p} & \equiv dt\frac{\partial L}{\partial n_{p}}. \end{array}$$

The gradient in *n* of θ therefore increments in time as

(27) $$\begin{array}{cc} \frac{\partial \left(\theta_{p}+d\theta_{p}\right)}{\partial n_{q}} & =\frac{\partial \theta_{p}}{\partial n_{q}}+dt\frac{\partial L^{2}}{\partial n_{p}\partial n_{q}}. \end{array}$$

Contraction of Equation (27) with the antisymmetric symbol in *p* and *q* vanishes

(28) $$\begin{array}{cc} \frac{d}{dt}\epsilon_{pq}\frac{\partial \theta_{p}}{\partial n_{q}} & =0, \end{array}$$

so the circulation of θ is the same everywhere as at the fixed points.

From Equation (20), it is required to be the case that $\partial \theta_{p}/\partial n_{q}=\partial^{2}S_{\mathrm{eff}}/\partial n_{p}\partial n_{q}\equiv g_{pq}^{-1}$, the inverse of the Fisher metric [41], symmetric by construction and thus giving $\epsilon_{pq}g_{pq}^{-1}=0$. The only difference between the fixed point and any other point is that for distant points we are relying on Hamiltonian trajectories to evaluate θ, whereas at the fixed point, the Fisher metric may be calculated by means not relying on the large-deviation saddle-point approximation. Therefore, Equation (28) may be read as a check that symmetry of the Fisher metric is preserved by Hamiltonian trajectories directly from the symmetric partial derivative of L in Equation (27).

### 3.2. The Stationary Distribution and Macrostates

Up to this point only the Lyapunov role (4) of the relative entropy has been developed. While the increase of $-D\;\left(\rho \|\underline{\rho }\right)$ has the appearance of the classical 2nd law, we can understand from three observations that this relative entropy is not the desired generalization of the entropy state function of classical thermodynamics to express the phenomenology of multi-level systems:

1. The relative entropy $-D\;\left(\rho \|\underline{\rho }\right)$ is a functional on arbitrary distributions, like the Shannon entropy that is a special case. It identifies no concept of macrostate, and has no dependence on state variables.
2. In a multi-level system that may have arbitrarily fine-grained descriptions, there is no upper limit to $D\;\left(\rho \|\underline{\rho }\right)$, and no appearance of the system scale at any particular level, which characterizes state-function entropies.
3. Equation (4) describes a deterministic increase of relative entropy; the large-deviation role of entropy as a log-probability for macrostate fluctuations [44] does not appear.

The step that has not yet been taken in our construction is, of course, the identification of a macrostate concept. Here, we depart from the usual development based on conservation laws, and follow Gell-Mann and Lloyd [68,69] in claiming that the concept of macrostate is not inherent in features of a system’s dynamics, but requires one to explicitly choose a procedure for aggregation or coarse-graining—what they call a “judge”—as part of the commitment to which phenomenology is being described.

We will put forth the definition of macrostates as the tilted distributions arising in generating functions for the stationary distribution $\underline{\rho }$. In the case of generating functions for number, these are the distributions appearing in Equation (14), which we will denote by $\rho^{\left(\bar{n}\right)}$. They are the least-improbable distributions with a given non-stationary mean to arise through aggregate microscopic fluctuations, and therefore dominate the construction of the large-deviation probability.

(The justification for this interpretation, as one entailed by the leading-exponential definition of the large-deviation approximation, is found in the conditional dependence structure of the Hamilton–Jacobi representation. If the large-deviation function for $\underline{\rho }$ exists and is continuous, its gradient is the vector field formed from the conjugate momentum variable θ to *n* at each point. The value $-\log\underline{\rho }$ is reached cumulatively as ∫θ·dn along the stationary Hamiltonian contours, meaning that the leading probability to reach each point is conditionally dependent only on earlier points on the contour. Concentration of conditional dependence within the stationary Hamiltonian trajectories identifies the bundles of microtrajectories that pass through the distributions indexed by the macrostates as carrying the leading probability for all further escapes, which is what we mean by these distributions being “least-improbable” under fluctuations. The classic reference for eikonal treatment of escapes for boundary-value problems and first passages is [62]. Illustrations of ways the ray method can fail to estimate a large-deviation function for non-equilibrium distributions are found in [89,90]. Further discussion of stationary Hamiltonian rays as reductions to equivalent one-dimensional problems, which may be easily understood in terms of balance of up-going and down-going probability flows, may be found in [91].)

The extensive state variable associated with this definition of macrostate is the tilted mean from Equation (18), which we will denote $\bar{n}=n_{\underline{\rho }}\;\left(\theta \right)$. If $\underline{\rho }$ and $\rho^{\left(\bar{n}\right)}$ are sharply peaked—the limit in which the large-deviation approximation is informative—the relative entropy of the macrostate is dominated at the saddle point of $\rho^{\left(\bar{n}\right)}$, where $\log\rho^{\left(\bar{n}\right)}∼0$, and thus

(29) $$\begin{array}{cc} D\;\left(\rho^{\left(\bar{n}\right)}\|\underline{\rho }\right) & ∼{\left.-\log{\underline{\rho }}_{n}\right|}_{n∼\bar{n}}={\underline{S}}_{\mathrm{eff}}\;\left(\bar{n}\right). \end{array}$$

The general relative entropy functional, applied to the macrostate, becomes the *entropy state function* ${\underline{S}}_{\mathrm{eff}}$, which takes as its argument the extensive state variable $\bar{n}$. Moreover, because probability under $\underline{\rho }$ is concentrated on configurations with the scale that characterizes the system, tilted means $\bar{n}$ that are not suppressed by very large exponential probabilities will have comparable scale. If $\bar{n}$ is also the scale factor in the large-deviations function (a property that may or may not hold, depending on the system studied), then ${\underline{S}}_{\mathrm{eff}}∼\bar{n}$ in scale, and the entropy state function now has the characteristic scale of the mesoscale level of the process description.

The three classes of distributions that enter a thermodynamic description are summarized in Table 2.

#### 3.2.1. Coherent States, Dimensional Reduction, and the *f*-Divergence

A special case, which is illustrative for its simplicity and which arises for an important sub-class of stochastic CRNs, is the case when $\underline{\rho }$ is a *coherent state*, an eigenvector of the lowering operator *a*. Coherent states are the generating functions of product-form Poisson distributions, or cross-sections through such products if the transitions in the population process satisfy conservation laws. They are known [92] to be general solutions for CRN steady states satisfying a condition termed *complex balance*, and the fixed points associated with such stationary distributions are also known to be unique and interior (no zero-expectations for any $n_{p}$) [72].

Let $\underline{n}$ be the eigenvalue of the stationary coherent state: $a\left|\rho^{\left(\underline{n}\right)}\right)=\underline{n}\left|\rho^{\left(\underline{n}\right)}\right)$. Then the mean in the tilted distribution lies in a simple exponential family, $\bar{n}\equiv n_{\underline{\rho }}\;\left(\theta \right)=e^{\theta }\underline{n}$ (component-wise), and the tilted macrostate $\rho^{\left(\bar{n}\right)}$ is also a coherent state: $a\left|\rho^{\left(\bar{n}\right)}\right)=\bar{n}\left|\rho^{\left(\bar{n}\right)}\right)$.

The logarithm of a product-form Poisson distribution in Stirling’s approximation is given by

(30) $$\begin{array}{cc} -\log\rho_{n}^{\left(\bar{n}\right)} & \approx n\cdot \log\left(\frac{n}{\bar{n}}\right)-\left(n-\bar{n}\right)\cdot 1\equiv D_{f}\;\left(n\|\bar{n}\right). \end{array}$$

$D_{f}\;\left(n\|\bar{n}\right)$, known as the *f*-divergence, is a generalization of the Kullback–Leibler divergence to measures such as *n* which need not have a conserved sum. The Lyapunov function from Equation (4) reduces in the same Stirling approximation to

(31) $$\begin{array}{cc} D\;\left(\rho^{\left(\bar{n}\right)}\|\underline{\rho }\right)\; & \approx D_{f}\;\left(\bar{n}\|\underline{n}\right), \end{array}$$

giving ${\underline{S}}_{\mathrm{eff}}\;\left(\bar{n}\right)$ in Equation (29). On coherent states, the Kullback–Leibler divergence on distributions, which may be of arbitrarily large dimension, reduces to the *f*-divergence on their extensive state variables which have dimension *P*.

The coherent states play a much more general role than their role as exact solutions for the restricted case of complex-balanced CRNs. In the Doi–Peliti 2-field functional integral formalism [93,94,95,96] for generating functionals over discrete-state stochastic processes, the coherent states form an over-complete basis in the Peliti representation of unity. The saddle-point approximation on trajectories, which yields the classical actions (25) and the resulting Hamilton–Jacobi equations, approximates expectations in the exact distribution by those in the nearest coherent-state basis element. Observables in macrostates are thus mapped to observables in coherent states; although, in cases when the coherent state is not an exact solution, the saddle-point condition may be sensitive to which observable is being evaluated.

#### 3.2.2. Multiple Fixed Points and Instantons

Systems with multiple metastable fixed points correspond to non-convex $\underline{\rho }$ and thus multiple modes. For these, monotone decrease of $\dot{D}\;\left(\rho \|\underline{\rho }\right)$ in Equation (4) does not entail monotonicity of the *f*-divergence in Equation (31). In such systems, first passages between basins of attraction are solutions to the Hamiltonian Equations (23) and (24) with momentum coordinate θ≠0. Along these ${\underline{S}}_{\mathrm{eff}}\;\left(\bar{n}\right)$ increases, and that increase is what is sometimes termed the “violation of the 2nd law”.

For unimodal $\underline{\rho }$, the θ≠0 large-deviation trajectories have a separate use and interpretation from the relaxation trajectories at θ=0 that give the classical 2nd law in Equation (49). For multi-modal $\underline{\rho }$, a special sub-class of θ≠0 trajectories, those known as *instantons* being responsible for first-passages between fixed-points [63,64], must be used to refine the interpretation of classical relaxation trajectories. That refinement relates the transient increases in the large-deviation function to the deterministic 2nd law (4) that continues to apply.

This section briefly introduces the Legendre duality that defines first-passage probabilities in metastable systems, arriving at the chain rule for entropy that separates the roles of classical and instanton trajectories. Let $\underline{n}$ be a fixed point of the Hamiltonian equations for $\underline{\rho }$, and denote by $n_{\underline{\rho }}\;\left(\theta |\underline{n}\right)$ the values of classical state variables $\bar{n}$ obtained along θ≠0 trajectories from $\underline{n}$. Call these *escape trajectories*. The set of all $\bar{n}$ is partitioned among basins of repulsion from fixed points. Saddle points and escape separatrices are limit points of escapes from two or more basins.

Within one such basin, we may construct ${\underline{S}}_{\mathrm{eff}}$ as a Legendre transform of a summand $\Gamma_{\underline{n}}$ in the overall CGF, as

(32) $$\begin{array}{cc} {\underline{S}}_{\mathrm{eff}}\;\left(\bar{n}\right) & =\max_{\theta }\left\{\theta \;\bar{n}+\Gamma_{\underline{n}}\;\left(\theta \right)\right\}. \end{array}$$

θ ranges only over the values that arise on escape trajectories from $\underline{n}$, which generally are bounded [91], and within that range

(33) $$\begin{array}{c} {\left.-\frac{\partial \Gamma_{\underline{n}}}{\partial \theta }\right|}_{\theta \left(\bar{n}\right)}=\bar{n}. \end{array}$$

Next, let the function $\underline{n}\;\left(\bar{n}\right)$ denote the fixed point to which a trajectory with θ≡0 relaxes, starting from $\bar{n}$. Note that the maps $\underline{n}\;\left(\bar{n}\right)$ and $n_{\underline{\rho }}\;\left(\theta |\underline{n}\right)$ need not be reflexive. That is, we may have $n_{\underline{\rho }}\;\left(\theta |\underline{n}\;\left(\bar{n}\right)\right)\neq \bar{n}$ for any θ, because escape and relaxation separatrices may differ. From the large-deviation identification of $S_{\mathrm{eff}}\;\left(n\right)∼-{\left.\log\rho_{n}\right|}_{n\approx n}$, recognize that the large-deviation function for the stationary distribution is given by ${\underline{S}}_{\mathrm{eff}}\;\left(\underline{n}\right)=D\;\left(1_{\underline{n}}\|\underline{\rho }\right)$, where $1_{\underline{n}}$ denotes the special macrostate for which the stationary value is a fixed-point value $\underline{n}$.

Then, the expression (29) for the Kullback–Leibler divergence appearing in Equation (4) may be approximated to leading exponential order as

(34) $$\begin{array}{cc} D\;\left(\rho^{\left(\bar{n}\right)}\|\underline{\rho }\right) & ∼{\underline{S}}_{\mathrm{eff}}\;\left(\bar{n}\right)-{\underline{S}}_{\mathrm{eff}}\;\left(\underline{n}\;\left(\bar{n}\right)\right)+D\;\left(1_{\underline{n}\left(\bar{n}\right)}\|\underline{\rho }\right). \end{array}$$

Equation (34) is the chain rule for relative entropy. ${\underline{S}}_{\mathrm{eff}}\;\left(\underline{n}\;\left(\bar{n}\right)\right)-{\underline{S}}_{\mathrm{eff}}\;\left(\bar{n}\right)$ is the conditional entropy of the macrostate $\bar{n}$ relative to the macrostate $1_{\underline{n}\left(\bar{n}\right)}$ to which it is connected by a θ=0 Hamiltonian trajectory. $-D\;\left(1_{\underline{n}\left(\bar{n}\right)}\|\underline{\rho }\right)$ is the unconditioned entropy of $1_{\underline{n}\left(\bar{n}\right)}$ relative to $\underline{\rho }$.

Relaxation along θ=0 trajectories describes a classical 2nd law only for the conditional part of the relative entropy. Deterministic relaxation of the unconditioned entropy is derived from the refinement of the (as it turns out, only apparent) classical trajectory with an instanton sum. The general method is described in depth in [63] and [64] Ch.7, using path integrals that require too much digression to fit within the scope of this paper. The structure of the instanton sum, and in particular the way it creates a new elementary stochastic process at the macroscale for which $D\;\left(1_{\underline{n}\left(\bar{n}\right)}\|\underline{\rho }\right)$ is the Lyapunov function, will be explained in Section 4.2.2 following [67], after the behavior of ${\underline{S}}_{\mathrm{eff}}$ along θ=0 and θ≠0 trajectories has been characterized.

## 4. Population Processes with CRN-Form Generators

The results up to this point apply to general processes with discrete state spaces, normalizable stationary distributions, and some kind of system⊗environment tensor-product structure on states. For the next steps, we restrict to stochastic population processes that can be written in a form equivalent to chemical reaction networks [71,72,97]. CRNs are an expressive enough class to include many non-mechanical systems such as evolving Darwinian populations [81], and to implement algorithmically complex processes [98]. Yet they possess generators with a compact and simple structure [73,74], in which similarities of microstate and macrostate phenomena are simply reflected in the formalism.

### 4.1. Hypergraph Generator of State-Space Transitions

Population states n give counts of individuals, grouped according to their types *p* which are termed *species*. Stochastic chemical reaction networks assume independent elementary events grouped into types termed *reactions*. Each reaction event removes a multiset (a collection of distinct individuals, which may contain more than one individual from the same species) of individuals termed a *complex* from the population, and places some (generally different) multiset of individuals back into it. The map from species to complexes is called the *stoichiometry* of the CRN. Reactions occur with probabilities proportional per unit time to *rates*. The simplest rate model, used here, multiplies a half-reaction rate constant by a combinatorial factor for proportional sampling without replacement from the population to form the complex, which in the mean-field limit leads to mass-action kinetics.

Complexes will be indexed with subscripts i,j,…, and an ordered pair such as ji labels the reaction removing *i* and creating *j*. With these conventions, the Liouville-operator representation of the generator from Equation (13) takes the form [73,74]

(35) $$\begin{array}{cc} -L & =\sum_{ji}k_{ji}\left[\psi_{j}\;\left(a^{†}\right)-\psi_{i}\;\left(a^{†}\right)\right]\psi_{i}\;\left(a\right)\\ \; & \equiv \psi^{T}\;\left(a^{†}\right)A\;\psi \;\left(a\right). \end{array}$$

$\left\{k_{ji}\right\}$ are half-reaction rate constants, organized in the second line into an adjacency matrix A on complexes. $\psi \equiv \left[\psi_{i}\right]$ is a column vector with components $\psi_{i}\;\left(a\right)\equiv \prod_{p}a_{p}^{y_{ip}}\equiv a^{Y_{i}}$ that, in the Doi algebra, produce the combinatorial factors and state shifts reflecting proportional sampling without replacement. Here, $Y\equiv \left[y_{ip}\right]$ is the matrix of stoichiometric coefficients, with entry $y_{ip}$ giving the number of individuals of species *p* that make up complex *i*. Further condensing notation, $Y_{i}\equiv \left[y_{ip}\right]$ are column vectors in index *p*, and $Y_{p}\equiv \left[y_{ip}\right]$ are row vectors on index *i*. $a^{Y_{i}}$ is understood as the component-wise product of powers $a_{p}^{y_{ip}}$ over the species index *p*. $\psi^{T}$ is a transpose (row) vector, and vector and matrix products are used in the second line of Equation (35).

The generator (35) defines a hypergraph [99] in which A is the adjacency matrix on an ordinary graph over complexes known as the *complex network* [97,100]. The stoichiometric vectors $\left\{Y_{i}\right\}$, defining complexes as multisets, make the edges in A directed hyper-edges relative to the population states that are the Markov states for the process. (Properly, the generator should be called a “directed multi-hypergraph” because the complexes are multisets rather than sets.) The concurrent removal or addition of complexes is the source of both expressive power and analytic difficulty provided by hypergraph-generators.

A master Equation (1) acting in the state space rather than on the generating function may be written in terms of the same operators, as

(36) $$\begin{array}{cc} {\dot{\rho }}_{n} & =\psi^{T}\;\left(e^{-\partial /\partial n}\right)A\mathrm{diag}\left[\psi \;\left(e^{\partial /\partial n}\right)\right]\Psi \;\left(n\right)\rho_{n}. \end{array}$$

Here, a formal shift operator $e^{\partial /\partial n}\equiv \left[e^{\partial /\partial n_{p}}\right]$ is used in place of an explicit sum over shifted indices, and $\psi_{i}\;\left(e^{\pm \partial /\partial n}\right)=e^{\pm Y_{i}^{T}\partial /\partial n}$ creates shifts by the stoichiometric vector $Y_{i}$. $\mathrm{diag}\left[\psi \right]$ refers to the matrix with diagonal entries given by the components $\psi_{i}$.

In the master equation, the combinatorial factors must be given explicitly. These are written as a vector $\Psi \equiv \left[\Psi_{i}\right]$ with components $\Psi_{i}\;\left(n\right)\equiv \prod_{p}\left[n_{p}!/\left(n_{p}-y_{ip}\right)!\right]\equiv n^{\underline{Y_{i}}}$ that are falling factorials from n, denoted with the underscore as $n^{\underline{Y_{i}}}$.

The matrix elements of Equation (1) may be read off from Equation (36) in terms of elements in the hypergraph, as

(37) $$\begin{array}{cccc} w_{n^{\prime }n}^{\left(ji\right)}\; & \equiv k_{ji}\Psi_{i}\;\left(n\right)\; & \mathrm{where}\;n^{\prime }\; & =Y_{j}-Y_{i}+n, \end{array}$$

between all pairs $\left(n^{\prime },n\right)$ separated by the stoichiometric difference vector $Y_{j}-Y_{i}$. If multiple reactions produce transitions between the same pairs of states, the aggregate rates become

(38) $$\begin{array}{cc} w_{n^{\prime }n} & \equiv \sum_{ji\;|\;Y_{j}-Y_{i}=n^{\prime }-n}w_{n^{\prime }n}^{\left(ji\right)}. \end{array}$$

In this way, a finite adjacency matrix A on complexes may generate an infinite-rank transition matrix T, which is the adjacency matrix for an ordinary graph over states. We will see that for CRNs, the hypergraph furnishes a representation for macrostates similar to the representation given by the simple graph for microstates.

From Equation (38), marginal transition rates $w_{n_{s}n_{s}^{\prime }}^{s}$ for the system may be defined using the average (2) over $\rho^{e|s}$. Note that the dependence of the activity products $\Psi_{i}$ on species *p* within the system remains that of a falling factorial, even if the average over activities of species in the environment is complicated. Denote by $\Psi^{s}\;\left(n_{s}\right)$ and $\psi^{s}\;\left(e^{\pm \partial /\partial n_{s}}\right)$ the restrictions of the activity and shift functions to the species in the system *s*, and by $k_{ji}^{s}\;\left(\rho^{e|s}\right)$ the rate constants after computing the sum in Equation (2) over the index for species in the environment.

#### 4.1.1. Descaling of Transition Matrices for Microstates

Proofs of monotonic change, whether of total relative entropy (1) or for the components of entropy change partitioned out in Section 2.4, take a particularly simple form for CRNs generated by finitely many reactions. They make use of finite cycle decompositions of the current through any microstate or complex, which are derived from descaled adjacency matrices.

The descaling that produces a transition matrix that annihilates the uniform measure on both the left and the right is

(39) $$\begin{array}{cc} \hat{T} & \equiv \psi^{T}\;\left(e^{-\partial /\partial n}\right)A\mathrm{diag}\left[\psi \;\left(e^{\partial /\partial n}\right)\right]\Psi \;\left(n\right)\mathrm{diag}\left[{\underline{\rho }}_{n}\right] \end{array}$$

for the whole mesoscale, or

(40) $$\begin{array}{cc} {\hat{T}}^{s} & \equiv \psi^{sT}\;\left(e^{-\partial /\partial n_{s}}\right)A^{s}\mathrm{diag}\left[\psi^{s}\;\left(e^{\partial /\partial n_{s}}\right)\right]\Psi^{s}\;\left(n_{s}\right)\mathrm{diag}\left[{\bar{\rho }}_{n_{s}}^{s}\right] \end{array}$$

for the subsystem *s*, by definition of the stationary states. $A^{s}$ in Equation (40) is the adjacency matrix on complexes that gives rate constants $w_{n_{s}n_{s}^{\prime }}^{s}$ from Equation (2). These descalings are familiar as the ones leading to the dual time-reversed generators in the fluctuation theorem for housekeeping heat [52].

We return to use the descaled microstate transition matrices (39) and (40) in monotonicity proofs for general CRNs in Section 5; but, before doing that, we use the descaling in the state space to motivate an analogous and simpler descaling for macrostates at the level of the hypergraph. That descaling illustrates the cycle decomposition on finitely many states, though it only yields the *f*-Divergence of Equation (31) as a Lyapunov function for the complex-balanced CRNs.

#### 4.1.2. Descaling of Transition Matrices for Macrostates

The coherent states, which are the moment-generating functions of Poisson distributions and their duals in the Doi Hilbert space, are defined as

(41) $$\begin{array}{cccc} \left(\phi^{†}\right| & \equiv e^{-\phi^{†}\phi }\left(0\right|e^{\phi^{†}a} & \left|\phi \right) & \equiv e^{a^{†}\phi }\left|0\right), \end{array}$$

where $\phi \equiv \left[\phi_{p}\right]$ is a vector of (generally complex) numbers and $\phi^{†}$ is its Hermitian conjugate. They are eigenstates respectively of the raising and lowering operators with eigenvalues $\left(\phi^{†}\right|a_{p}^{†}=\left(\phi^{†}\right|\phi_{p}^{*}$ and $a_{p}\left|\phi \right)=\phi_{p}\left|\phi \right)$.

On the constant trajectories corresponding to a fixed point of the Hamiltonian Equations (23) and (24), $\phi^{†}\equiv 1$ and $\phi \equiv \underline{\phi }=\underline{n}$, the fixed-point number. We may descale the coherent-state parameters ϕ and $\phi^{†}$, which correspond to classical state variables, by defining

(42) $$\begin{array}{cccc} \phi_{p} & \equiv {\underline{\phi }}_{p}\varphi_{p} & \phi_{p}^{*}{\underline{\phi }}_{p} & \equiv \varphi_{p}^{*}. \end{array}$$

In vector notation, $\phi \equiv \mathrm{diag}\left[\underline{\phi }\right]\varphi$, $\phi^{†}\mathrm{diag}\left[\underline{\phi }\right]\equiv \varphi^{†}$. The scaling (42) was introduced by Baish [101] to study dualities of correlation functions in Doi–Peliti functional integrals.

The compensating descaling of the adjacency matrix

(43) $$\begin{array}{cc} \hat{A}\; & \equiv A\mathrm{diag}\left[\psi \;\left(\underline{\phi }\right)\right] \end{array}$$

may be compared with Equation (39) for T. A similar descaling may be done for $A^{s}$ using ${\bar{\rho }}^{s}$. Henceforth, we omit the duplicate notation, and carry out proofs with respect to whatever is the stationary distribution for a given adjacency matrix and hypergraph.

The coherent-state action (25) with Liouville operator (35) has a symmetric form in descaled variables that is particularly useful for understanding the relaxation and escape trajectories in the Hamilton–Jacobi system:

(44) $$\begin{array}{cc} S & =\int dt\left\{\phi^{†}\mathrm{diag}\left[\underline{\phi }\right]d_{t}\varphi -\psi^{T}\;\left(\phi^{*}\right)\hat{A}\;\psi \;\left(\varphi \right)\right\}. \end{array}$$

#### 4.1.3. Equations of Motion and the L=0 Manifold

The Hamiltonian equations derived by variation of the action (44) can be written

(45) $$\begin{array}{cccc} \mathrm{diag}\left[\underline{\phi }\right]\dot{\varphi }\; & =\frac{\partial \psi^{T}}{\partial \phi^{†}}\hat{A}\psi \;\left(\varphi \right)\; & \; & \underset{\phi^{†}=1}{\to }Y\hat{A}\psi \;\left(\varphi \right)\\ {\dot{\phi }}^{†}\mathrm{diag}\left[\underline{\phi }\right]\; & =-\psi^{T}\;\left(\phi^{*}\right)\hat{A}\frac{\partial \psi }{\partial \varphi }\; & \; & \underset{\varphi =1}{\to }-\psi^{T}\;\left(\phi^{*}\right)\hat{A}Y^{T}. \end{array}$$

The general solution is shown first in each line and then particular limiting forms are shown.

The sub-manifold $\phi^{†}\equiv 1$ contains all relaxation trajectories for any CRN. It is dynamically stable because A is a stochastic matrix on the left, and thus $\psi^{T}\;\left(1\right)\hat{A}=0$ in the second line of Equation (45). The image of YA is called the *stoichiometric subspace*, and its dimension is denoted $s\equiv \dim\left[\mathrm{im}\left(YA\right)\right]$.

An important simplifying property of some CRNs is known as *complex balance* of the stationary distributions. It is the condition that the fixed point $\psi \;\left(\underline{n}\right)\in \ker A$ and not only ∈kerYA. Since $\underline{n}=\underline{\phi }$ corresponds to φ=1 and thus $\psi \;\left(\varphi \right)=1$, $1\in \ker\hat{A}$ and $\dot{\varphi }=0$ in the first line of Equation (45) at any $\phi^{†}$. Complex-balanced CRNs (with suitable conditions on A to ensure ergodicity on the complex network [97]) always possess unique interior fixed points [72,102] and simple product-form stationary distributions at these fixed points [92]. Complex balance can be ensured at all parameters if a topological character of the CRN known as *deficiency* equals zero, but may also be true for nonzero-deficiency networks for suitably tuned parameters. In this work, nothing requires us to distinguish these reasons for complex balance.

For non-complex-balanced stationary solutions, although escapes may have φ=1 as initial conditions, that value is not dynamically maintained. Recalling the definition (12) of the number operator, the field $n_{p}=\phi_{p}^{†}\phi_{p}$, so non-constant ϕ is required for instantons to escape from stable fixed points and terminate in saddle fixed points, in both of which limits $\phi^{†}\to 1$.

All relaxations and also the escape trajectories from fixed points (the instantons) share the property that L≡0 for a CRN with time-independent parameters. This sub-manifold separates into two branches, with $\phi^{†}\equiv 1$ for relaxations and $\phi^{†}\neq 1$ for escapes. (We can see that this must be true because −L is a Hamiltonian conserved under the equations of motion, and because instantons trace the values of *n* and θ in the stationary distribution $\underline{\rho }$, which must then have L=0 to satisfy the Hamilton–Jacobi Equation (22).)

#### 4.1.4. The Schlögl Cubic Model to Illustrate

The features of CRNs with multiple metastable fixed points are exhibited in a cubic 1-species model introduced by Schlögl [103], which has been extensively studied [73,74,90] as an example of dynamical bistability.

The reaction schema in simplified form is

(46) $$\begin{array}{ccc} ⌀\; & \overset{k_{d}}{\underset{{\bar{k}}_{d}}{⇌}}A\; & 2A\overset{k_{c}}{\underset{{\bar{k}}_{c}}{⇌}}3A. \end{array}$$

We choose rate constants so that the mass-action equation for the number of *A* particles, given by *n*, is

(47) $$\dot{n}=\left(n_{3}-n\right)\left(n_{2}-n\right)\left(n_{1}-n\right).$$

The three fixed points are $\underline{n}\in \left\{n_{1},n_{2},n_{3}\right\}$, of which $n_{1}$ and $n_{3}$ are stable, and $n_{2}$ is a saddle.

**Figure 2:**
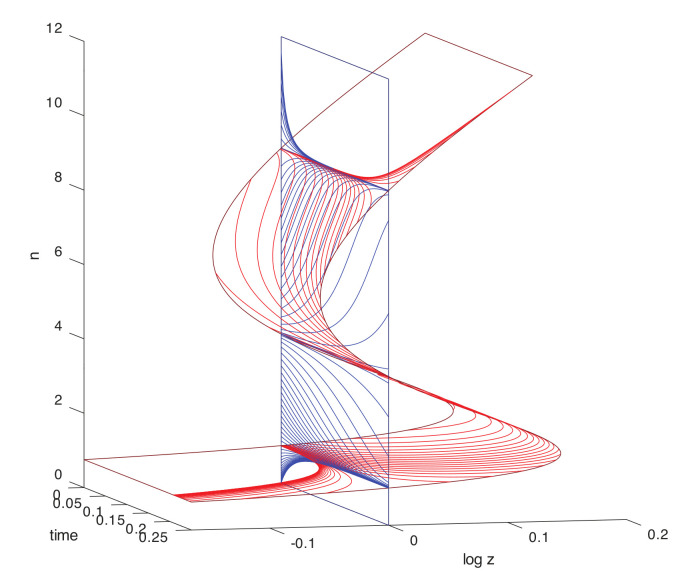
The two branches of the L=0 manifold, corresponding to the rate Equation (47). $\phi^{†}\equiv 1$ (blue); $\phi^{†}\neq 1$ (red). Trajectories at common *n* have the same value on the vertical axis. Time is shown moving toward the front.

The relaxation and escape branches of the L=0 manifold are shown in Figure 2. Because the Schlögl model is 1-dimensional, the condition L=0 fixes $\theta \;\left(n\right)$ along escape trajectories. Because the stochastic model is a birth-death process, it is also exactly solvable [104]. We will return to the example in Section 6.2 to study properties of the intensive and extensive thermodynamic potentials for it.

### 4.2. Large-Deviation and Lyapunov Roles of the Effective Action

The role of entropy in the understanding of Boltzmann and Gibbs was that of a Lyapunov function [39,86], accounting for unidirectionality from microscopically reversible mechanics. The much later understanding of entropy in relation to fluctuations [43,44]—precisely the opposite of deterministic evolution—is that of a large-deviation function.

The chain rule in Equation (34) of Section 3.2.2 relates the conditional relative entropy associated with quasi-deterministic relaxation to an additional unconditioned entropy of metastable states that are stable fixed points of the Hamiltonian dynamical system. Here, we complete the description of the relation between the Lyapunov and large-deviation roles of the macrostate entropy (29), and show how the sum over instantons rather than a single Hamiltonian trajectory results in deterministic increase of the unconditioned relative entropy $D\;\left(1_{\underline{n}}\|\underline{\rho }\right)$. In the Hamilton–Jacobi representation, this means constructing relations between the $\phi^{†}=1$ and the $\phi^{†}\neq 1$ branches of the L=0 manifold. For special cases, such as CRNs with detailed balance or one-dimensional systems, the mapping is one of simple time reversal of the *n* fields in trajectories. More generally, even for complex-balanced CRNs where the Lyapunov and large-deviation functions are the same, the relations between relaxation and escape trajectories become more variable.

#### 4.2.1. Convexity Proof of the Lyapunov Property of Macrostate Entropy in Hamilton–Jacobi Variables

Begin with the dynamics of the relative entropy ${\underline{S}}_{\mathrm{eff}}\;\left(\bar{n}\right)$ from Equation (29), as the state variable $\bar{n}$ evolves along a relaxation solution to the Hamiltonian equations of motion. The properties of ${\underline{S}}_{\mathrm{eff}}\;\left(\bar{n}\right)$ follow from its construction as a large-deviation function along escape trajectories. From Equation (20), the time derivative of ${\underline{S}}_{\mathrm{eff}}$ along an escape trajectory is given by

(48) $$\begin{array}{cc} \frac{{\underline{S}}_{\mathrm{eff}}\;\left(n\right)}{\partial n}{\dot{n}}_{\mathrm{esc}} & =\theta_{\underline{\rho }}\;\left(n\right)\cdot {\dot{n}}_{\mathrm{esc}}\equiv {\left.{\dot{\underline{S}}}_{\mathrm{eff}}\right|}_{\mathrm{esc}}. \end{array}$$

Hamiltonian trajectories are least-improbable paths of fluctuation, with escapes conditionally dependent along trajectories (see Section 3.2). The conditional probability to extend a path, having reached any position along the path, is always positive, giving ${\left.{\dot{\underline{S}}}_{\mathrm{eff}}\right|}_{\mathrm{esc}}\geq 0$ in Equation (48).

Next compute ${\dot{\underline{S}}}_{\mathrm{eff}}$ along a relaxation trajectory, for simplicity considering A (an equivalent construction exists for $A^{s}$ in terms of ${\bar{\rho }}^{s}$). The continuum limit of the relative entropy from Equation (4) replaces $\sum_{n}\to \int dn$, and continuously-indexed $-\log\rho_{n}^{\left(\bar{n}\right)}$ and $-\log{\underline{\rho }}_{n}$ are defined through the large-deviation functions.

Writing the CRN Liouville operator (35) in coherent-state arguments, the time dependence is evaluated as

(49) $$\begin{array}{cc} \dot{D}\;\left(\rho^{\left(\bar{n}\right)}\|\underline{\rho }\right)\; & \to \int d^{s}\;n\;\log\left(\frac{\rho_{n}^{\left(\bar{n}\right)}}{{\underline{\rho }}_{n}}\right){\dot{\rho }}_{n}^{\left(\bar{n}\right)}\\ \; & ∼\int d^{s}\;n\;\log\left(\frac{\rho_{n}^{\left(\bar{n}\right)}}{{\underline{\rho }}_{n}}\right)\left[\psi^{T}\;\left(z\right)A\psi \;\left(\frac{n}{z}\right)\right]\rho_{n}^{\left(\bar{n}\right)}\\ \; & =\sum_{ji}k_{ji}\int d^{s}\;n\;\log\left(\frac{\rho_{n}^{\left(\bar{n}\right)}}{{\underline{\rho }}_{n}}\right)e^{\theta \left(Y_{j}-Y_{i}\right)}\psi_{i}\;\left(n\right)\rho_{n}^{\left(\bar{n}\right)}\\ \; & \approx \sum_{ji}k_{ji}\int d^{s}\;n\;{\left.\frac{\partial }{\partial n_{p}}\log\left(\frac{\rho_{n}^{\left(\bar{n}\right)}}{{\underline{\rho }}_{n}}\right)\right|}_{\bar{n}}{\left(n-\bar{n}\right)}_{p}\\ \; & \times {\left(n-\bar{n}\right)}_{q}{\left.\frac{-\partial^{2}\log\rho_{n}^{\left(\bar{n}\right)}}{\partial n_{q}\partial n_{r}}\right|}_{\bar{n}}{\left(Y_{j}-Y_{i}\right)}_{r}\psi_{i}\;\left(n\right)\rho_{n}^{\left(\bar{n}\right)}\\ \; & =\sum_{ji}k_{ji}{\left.\frac{-\partial \log{\underline{\rho }}_{n}}{\partial n_{p}}\right|}_{\bar{n}}{\left(Y_{j}-Y_{i}\right)}_{p}\psi_{i}\;\left(\bar{n}\right)\\ \; & ={\left.\frac{-\partial \log{\underline{\rho }}_{n}}{\partial n}\right|}_{\bar{n}}\cdot Y\hat{A}\psi_{i}\;\left(\bar{n}\right)\\ \; & =\theta_{\underline{\rho }}\;\left(\bar{n}\right)\cdot {\dot{n}}_{\rho^{\left(\bar{n}\right)}}\;\left(0\right)\\ \; & ={\left.{\dot{\underline{S}}}_{\mathrm{eff}}\;\left(\bar{n}\right)\right|}_{\mathrm{rel}}. \end{array}$$

In Equation (49), *z* abbreviates $z_{\rho^{\left(\bar{n}\right)}}\;\left(n\right)\equiv \exp\theta_{\rho^{\left(\bar{n}\right)}}\;\left(n\right)$ from Equation (22), for the $S_{\mathrm{eff}}$ that is the continuum approximation of $-\log\rho^{\left(\bar{n}\right)}$. The third through fifth lines expand A explicitly in terms of rate constants $k_{ji}$ following Equation (35), to collocate all terms in θ≡logz in the Liouville operator. The fourth line expands $\log\left(\rho^{\left(\bar{n}\right)}/\underline{\rho }\right)$ and θ to linear order in $n-\bar{n}$ in neighborhoods of the saddle point $\bar{n}$ of $\rho^{\left(\bar{n}\right)}$. The matrix $\left[-\partial^{2}\log\rho /\partial n_{q}\partial n_{r}\right]$ is the inverse of the Fisher metric that is the variance $\left[\langle {\left(n-\bar{n}\right)}_{p}{\left(n-\bar{n}\right)}_{q}\rangle \right]$ [41], so the product of the two is just the identity $\left[\delta_{pr}\right]$.

In the penultimate line of Equation (49), $\theta_{\underline{\rho }}\;\left(\bar{n}\right)$ is the value of $\partial {\underline{S}}_{\mathrm{eff}}/\partial n$ for the escape trajectory passing through $\bar{n}$, and now ${\dot{n}}_{\rho^{\left(\bar{n}\right)}}\;\left(0\right)$ is the velocity along the relaxation trajectory rather than the Hamilton–Jacobi escape solution at $\bar{n}$. Therefore, the net effect of the large-deviation approximation on relative entropy has been to replace escape with relaxation velocity vectors at a fixed value of θ Legendre dual to $\bar{n}$.

**Lemma** **1.** $\theta_{\underline{\rho }}\;\left(\bar{n}\right)\cdot {\dot{n}}_{\rho^{\left(\bar{n}\right)}}\;\left(0\right)\leq 0$.

**Proof.**  The proof follows from four observations:

1. **ℒ = 0:** As noted, for both escapes and relaxations, $L\;\left(\theta ,n\right)=0$.
2. **Convexity:** Both the potential value $\theta_{\underline{\rho }}$ for the escape trajectory, and the velocity $\dot{n}$ of the relaxation trajectory, are evaluated at the same location $\bar{n}=n_{\rho^{\left(\bar{n}\right)}}\;\left(0\right)$. The Liouville function $-L=\sum_{ji}k_{ji}\left(e^{\theta \left(Y_{j}-Y_{i}\right)}-1\right)\psi_{i}\;\left(n\right)$, with all $k_{ji}>0$, is convex on the *s*-dimensional sub-manifold of fixed *n*. L is bounded above at fixed *n*, and in order for cycles to be possible, shift vectors $\left(Y_{j}-Y_{i}\right)$ giving positive exponentials must exist for all directions of θ in the stoichiometric subspace. Therefore, L→−∞ at large $\left|\theta \right|$ in every direction, and the region L>0 at fixed *n* is bounded. The boundary $L\;\left(\theta ,n\right)=0$ at fixed *n* is likewise convex with respect to θ as affine coordinates, and L>0 is its interior.
3. **Chord:** The vector $\left(\theta -0\right)$ is thus a chord spanning the L=0 sub-manifold of co-dimension 1 within the *s*-dimensional manifold of fixed *n*.
4. **Outward-directedness:** The equation of motion $\dot{n}=-\partial L/\partial \theta$ gives $\dot{n}\;\left(\theta ,n\right)$ as the outward normal function to the surface $L\;\left(\theta ,n\right)=0$. The outward normal at θ=0 is the classical relaxation trajectory. Every chord $\left(\theta -0\right)$ of the surface lies in its interior, implying that $\theta_{\underline{\rho }}\;\left(n\right)\cdot \dot{n}\;\left(0,n\right)<0$ for any *n*, and thus $\theta_{\underline{\rho }}\;\left(\bar{n}\right)\cdot {\dot{n}}_{\rho^{\left(\bar{n}\right)}}\;\left(0\right)\leq 0$.

 □

The conditional part of the relative entropy, $-D\;\left(\rho^{\left(\bar{n}\right)}\|\underline{\rho }\right)$, is thus shown to be monotone increasing along relaxation trajectories, which is the Lyapunov role for the entropy state function familiar from classical thermodynamics. That increase ends when $\bar{n}$ terminates in the trajectory fixed point $\underline{n}\;\left(\bar{n}\right)$.

#### 4.2.2. Instantons and the Loss of Large-Deviation Accessibility from First Passages

The deterministic analysis of Equation (49) is refined by the inclusion of instanton trajectories through the following sequence of observations, completing the discussion begun in Section 3.2.2. Relevant trajectories are shown in Figure 1.

1. The 2nd law as formulated in Equation (4) is approximated in the large-deviation limit not by a single Hamiltonian trajectory, but by the sum of all Hamiltonian trajectories, from an initial condition. Along a single trajectory, $-D\;\left(\rho^{\left(\bar{n}\right)}\|\underline{\rho }\right)$ could increase or decrease.
$-D\;\left(\rho^{\left(\bar{n}\right)}\|\underline{\rho }\right)$ increases everywhere in the sub-manifold θ=0 of the manifold L=0, by Equation (49). This is the classical increase of (relative) entropy of Boltzmann and Gibbs. $-D\;\left(\rho^{\left(\bar{n}\right)}\|\underline{\rho }\right)$ decreases everywhere in the submanifold θ≠0 of the manifold L=0, by Equation (48). This is the construction of the log-probability for large deviations. These escape paths, however, simply lead to the evaluations of ${\underline{S}}_{\mathrm{eff}}\;\left(n\right)∼{\left.-\log{\underline{\rho }}_{n}\right|}_{n\approx n}$, the stationary distribution.
3. If a CRN has a single fixed point $\underline{n}$, there is a unique θ=0 trajectory from any starting $\bar{n}$ to it, and $-D\;\left(\rho^{\left(\bar{n}\right)}\|\underline{\rho }\right)$ increases deterministically by Equation (49) along that single path. The black trajectory with arrow converging exactly in a domain fixed point is such a path in Figure 1.
4. If a CRN has multiple fixed points and instantons, all trajectories are exponentially close to the exact θ=0 trajectory before they enter a small neighborhood around the terminus $\underline{n}\;\left(\bar{n}\right)$ of the exact trajectory; that is, they give the appearance of being the black deterministic trajectory in Figure 1. The trajectory sum is correspondingly close to the deterministic relaxation that increases the conditional entropy ${\underline{S}}_{\mathrm{eff}}\;\left(\underline{n}\;\left(\bar{n}\right)\right)-{\underline{S}}_{\mathrm{eff}}\;\left(\bar{n}\right)$ in Equation (34).
5. On longer times, however, the infinite sum of formally distinct Hamiltonian trajectories disperses into a sum over series of instantons making a random walk among fixed points, with an integral for each passage over the possible times at which the escape occurs. (See [64] Ch.7.) Such a sum is shown as a tree of colored first-passage trajectories in Figure 1. The “cross-sectional” sum at a single observation time over instantons distinguished by their escaping times gives the same result as a longitudinal line integral along a single instanton between the start time and the observation. That integral of $-\left(\partial \log\rho_{n}^{\left(\bar{n}\right)}/\partial n\right)\dot{n}$ through a full passage (escape instanton + succeeding relaxation) gives ${\underline{S}}_{\mathrm{eff}}\;\left(\underline{n}\right)-{\underline{S}}_{\mathrm{eff}}\;\left({\underline{n}}^{\prime }\right)=\log\left(w_{{\underline{n}}^{\prime }\underline{n}}^{\mathrm{macro}}/w_{\underline{n}{\underline{n}}^{\prime }}^{\mathrm{macro}}\right)$. The escape from fixed point $\underline{n}$ to a saddle between $\underline{n}$ and a fixed point ${\underline{n}}^{\prime }$ in an adjacent basin, which we denote $\bar{n}={\underline{n}}^{\prime }\;‡\;\underline{n}$, is an integral over Equation (48), while the relaxation from the saddle $\bar{n}$ to the fixed point ${\underline{n}}^{\prime }$ is an integral over Equation (49). These are the classical “entropy fluctuations” of stochastic thermodynamics.
6. The contribution to the probability of a trajectory from each instanton comes only from the θ≠0 sub-manifold, and is given by $w_{{\underline{n}}^{\prime }\underline{n}}^{\mathrm{macro}}∼e^{-\left[{\underline{S}}_{\mathrm{eff}}\;\left(\bar{n}\right)-{\underline{S}}_{\mathrm{eff}}\;\left(\underline{n}\right)\right]}\equiv e^{-\Delta {\underline{S}}_{\mathrm{eff}}}$, just the leaving rate from the macrostate $1_{\underline{n}}$. The result, upon coarse-graining to the macroscale (see Table 1 and the top diagram in Figure 1) where first-passages become instantaneous elementary events, is a new stochastic process on discrete states corresponding to the mesoscale Hamiltonian fixed points $\left\{\underline{n},{\underline{n}}^{\prime },\ldots \right\}$. The coarse-grained counterpart to $D\;\left(1_{\underline{n}\left(\bar{n}\right)}\|\underline{\rho }\right)$ from Equation (34) is the Lyapunov function reduced by a transition matrix $T^{\mathrm{macro}}$ with matrix elements $w_{{\underline{n}}^{\prime }\underline{n}}^{\mathrm{macro}}$. The coarse-graining and the reduction of $D\;\left(1_{\underline{n}\left(\bar{n}\right)}\|\underline{\rho }\right)$ are described in detail for a 2-basin example in [67].
7. The properties of $T^{\mathrm{macro}}$ are exactly those we have assumed for T as inputs to the mesoscale, completing the self-consistency of our recursively-defined multi-level model universe.

## 5. Cycle Decompositions and Non-Decrease of Intrinsic and Extrinsic Relative Entropies

A diagram technique was devised by Terrell Hill and co-authors [105,106] (summarized in [107]), to both construct steady-state densities and decompose the associated heat dissipation into contributions from fluxes around a basis of cycles [108]. The expression for dissipation and the diagram method to obtain a basis of cycles were adopted in the network theory of master equations by Schnakenberg [76].

Both the mean and the fluctuations in the environmental or housekeeping entropy changes may be approximated in either the long-time or the ensemble limit from the counting statistics of directed cycle completions [108,109], together with methods to account for the relative occupancy of states within each cycle. The latter, single-time ensemble method will be used here to derive the positive-semidefiniteness of the relative entropy and housekeeping terms in Equation (11). The form of the CRN generator leads to a decomposition of entropy changes into sums of densities convected around a basis of cycles by the stationary-state currents. Positivity is proved by convexity arguments similar to those for the standard fluctuation theorems [21,52,53], and the cycle decomposition expresses the duality relations of those theorems in terms of shifts forward or backward around cycles.

Section 4 constructed the relation between the hypergraph adjacency matrix A and stoichiometry *Y*, and the microstate transition matrix T. Each reaction in the hypergraph acts regularly as a *rule* on indefinitely many microstates, making CRNs an example of rule-based systems [110]. This section extends that mapping to cycle decompositions. For finitely-generated CRNs, there is a finite basis of either cyclic flows through complexes or stoichiometrically coupled cycles called hyperflows [77], with the interpretation of mass-action currents. From these, a (generally infinite) basis of finite-length cycles can be constructed for flows in the microstate space, which projects onto the basis in the hypergraph. We first use the finite basis of macrostate currents to prove monotonicity results for *f*-divergences where those exist, showing how the projection of cycles can result in a projection of the Lyapunov property between levels.

The parallelism between microstates and macrostates only holds, however, when the bases for currents at both levels contain only cycles. More generally, when macrostate currents include non-cyclic hyperflows, the analogy between the two levels is broken, leading to loss of the *f*-divergence as a Lyapunov function on macrostate variables and generally to much greater difficulty of solving for the stationary distribution on microstates. The conditions for different classes of flows on the hypergraph are the basis for a complexity classification of CRNs, and both the form of the large-deviation function and the symmetry between its Lyapunov and large-deviation roles differ across classes.

### 5.1. Complexity Classes and Cycle Decompositions of Stationary Currents on the Hypergraph

Remarkably, the properties of classical fixed points in the Hamilton–Jacobi representation are sufficient to classify CRNs in a hierarchy with three nested levels of complexity, according to the mass-action currents at the fixed points. Let $\underline{n}$ again denote the fixed point.

1. The CRNs with **detailed balance** are those in which $k_{ji}\psi_{i}\;\left(\underline{n}\right)=k_{ij}\psi_{j}\;\left(\underline{n}\right)$, for all pairs of complexes $\langle j,i\rangle$ connected by a reaction. Under the descaling (43), this condition is that ${\hat{A}}^{T}=\hat{A}$.
2. The CRNs with **complex balance** only require $\sum_{i}k_{ji}\psi_{i}\;\left(\underline{n}\right)=\sum_{i}k_{ij}\psi_{j}\;\left(\underline{n}\right)$, for each complex *j*, or under descaling, $\hat{A}\psi \;\left(1\right)=0$.
3. The general case requires only $YA\psi \;\left(\underline{n}\right)$, the condition of current balance at each species *p*, or under descaling, only $Y\hat{A}\psi \;\left(1\right)=0$.

The classes are summarized in Table 3,

#### 5.1.1. Complex Balance and Relations of the Lyapunov and Large-Deviation Roles of $S_{\;\mathrm{eff}}$

It is known that complex-balanced CRNs (with technical conditions to ensure ergodicity on the complex network under A [97,111]) possess unique interior fixed points [72], and moreover that their exact steady states $\underline{\rho }$ are products of Poisson distributions or sections through such products [92] defined by conserved quantities under the stoichiometry. Their generating functions are the coherent states from Section 3.2.1. It is immediate that all classical states obtained by exponential tilts with parameter $z=e^{\theta }$ are likewise coherent states with state variables

(50) $$\begin{array}{cc} {\bar{n}}_{p}\; & \equiv e^{\theta_{p}}{\underline{n}}_{p}, \end{array}$$

and that the *f*-divergence of Equation (30) is the macrostate relative entropy function.

From the equations of motion (45), within either the $\phi^{†}\equiv 1$ or $\phi^{†}\neq 1$ sub-manifolds, the time derivatives of ${\underline{S}}_{\mathrm{eff}}$ along escape and relaxation trajectories, Equation (49) and Equation (48) respectively take the forms

(51) $$\begin{array}{cc} {\left.{\dot{\underline{S}}}_{\mathrm{eff}}\right|}_{\mathrm{rel}} & =\log\left(\frac{n}{\underline{n}}\right)\cdot {\dot{n}}_{\mathrm{rel}}=\log\left(\frac{n}{\underline{n}}\right)\cdot Y\hat{A}\psi \;\left(\frac{n}{\underline{n}}\right)\\ {\left.{\dot{\underline{S}}}_{\mathrm{eff}}\right|}_{\mathrm{esc}} & =\log\left(\frac{n}{\underline{n}}\right)\cdot {\dot{n}}_{\mathrm{esc}}=-\log\left(\frac{n}{\underline{n}}\right)\cdot Y{\hat{A}}^{T}\psi \;\left(\frac{n}{\underline{n}}\right). \end{array}$$

By Equation (50), $\psi_{i}$ appearing in Equation (51) evaluate to

(52) $$\begin{array}{cc} \psi_{i}\;\left(\frac{n}{\underline{n}}\right)\; & =e^{\theta Y_{i}}. \end{array}$$

Finite cycle decomposition of the steady-state current:

By definition of complex balance, $\hat{A}1=0$, in addition to the general condition that $1^{T}\hat{A}=0^{T}$. Any such matrix acting over finitely many complexes may be written as a sum of adjacency matrices for cyclic flows, which we index α, with positive coefficients ${\bar{ȷ}}_{\alpha }$ equal to the currents around those cycles in the stationary state. For the subclass with detailed balance, a decomposition in cycles of length 2 is always possible.

Letting $\sum_{ji|\alpha }$ denote the sum over directed links ji in order around the cycle α, the trajectory derivatives (51) may be decomposed as

(53) $$\begin{array}{cc} {\left.{\dot{\underline{S}}}_{\mathrm{eff}}\right|}_{\mathrm{rel}}=\theta \cdot {\dot{n}}_{\mathrm{rel}} & =-\sum_{\alpha }{\bar{ȷ}}_{\alpha }\sum_{ji|\alpha }e^{\theta Y_{i}}\cdot \log\left(\frac{e^{\theta Y_{i}}}{e^{\theta Y_{j}}}\right)\\ {\left.{\dot{\underline{S}}}_{\mathrm{eff}}\right|}_{\mathrm{esc}}=\theta \cdot {\dot{n}}_{\mathrm{esc}} & =\sum_{\alpha }{\bar{ȷ}}_{\alpha }\sum_{ji|\alpha }e^{\theta Y_{j}}\cdot \log\left(\frac{e^{\theta Y_{j}}}{e^{\theta Y_{i}}}\right). \end{array}$$

(· indicates the vector inner product over the species index *p*.)

Letting $\sum_{i|\alpha }$ denote the sum over all complexes in the cycle α, the complex activities (52) may be normalized to vectors $p_{\alpha }\equiv \left[p_{i|\alpha }\right]$ with unit measure, as

(54) $$p_{i|\alpha }\equiv \frac{e^{\theta Y_{i}}}{\sum_{j|\alpha }e^{\theta Y_{j}}}.$$

Then the trajectory derivatives (53), themselves the time derivatives of the *f*-divergence (34), may be written in terms of positive semidefinite KL divergences of the measures $p_{\alpha }$ from their own images advanced or retarded by one complex around each cycle:

(55) $$\begin{array}{cc} \theta \cdot {\left.\dot{n}\right|}_{\mathrm{rel}} & =-\sum_{\alpha }\left({\bar{ȷ}}_{\alpha }\sum_{i|\alpha }e^{\theta Y_{i}}\right)\sum_{ji|\alpha }p_{i|\alpha }\cdot \log\left(\frac{p_{i|\alpha }}{p_{j|\alpha }}\right)\\ \theta \cdot {\left.\dot{n}\right|}_{\mathrm{esc}} & =\sum_{\alpha }\left({\bar{ȷ}}_{\alpha }\sum_{i|\alpha }e^{\theta Y_{i}}\right)\sum_{ji|\alpha }p_{j|\alpha }\cdot \log\left(\frac{p_{j|\alpha }}{p_{i|\alpha }}\right). \end{array}$$

Non-negativity of the KL divergences in every term of Equation (55) recovers the monotonicities of ${\underline{S}}_{\mathrm{eff}}$ along relaxation and escape trajectories from Section 4.2.1. The total time derivatives are decomposed into terms over finitely many cycles, each independently having the same sign, a stronger decomposition than can be obtained from the convexity proofs for increase of relative entropy [76] for general CRNs.

Locally linear coordinates for classical entropy change in complex-balanced CRNs

Note that the cycle-KL divergences, multiplied by the density factors $\sum_{i|\alpha }e^{\theta Y_{i}}$, define a coordinate system that is locally invertible for the coordinates θ except at isolated points:

(56) $$\begin{array}{cc} x_{\alpha -} & \equiv \sum_{ji|\alpha }e^{\theta Y_{i}}\log\left(\frac{e^{\theta Y_{i}}}{e^{\theta Y_{j}}}\right)\\ x_{\alpha +} & \equiv \sum_{ji|\alpha }e^{\theta Y_{j}}\log\left(\frac{e^{\theta Y_{j}}}{e^{\theta Y_{i}}}\right). \end{array}$$

The time derivatives of ${\underline{S}}_{\mathrm{eff}}$ from Equation (53) may be written in these coordinates as a linear system,

(57) $$\begin{array}{cc} {\left.{\dot{\underline{S}}}_{\mathrm{eff}}\right|}_{\mathrm{rel}}={\dot{D}\;\left(e^{\theta Y}\|1\right)}_{\mathrm{rel}}=\theta \cdot {\dot{n}}_{\mathrm{rel}}\; & =-\sum_{\alpha }{\bar{ȷ}}_{\alpha }x_{\alpha -}\\ {\left.{\dot{\underline{S}}}_{\mathrm{eff}}\right|}_{\mathrm{esc}}={\dot{D}\;\left(e^{\theta Y}\|1\right)}_{\mathrm{esc}}=\theta \cdot {\dot{n}}_{\mathrm{esc}}\; & =\sum_{\alpha }{\bar{ȷ}}_{\alpha }x_{\alpha +}. \end{array}$$

**Figure 3:**
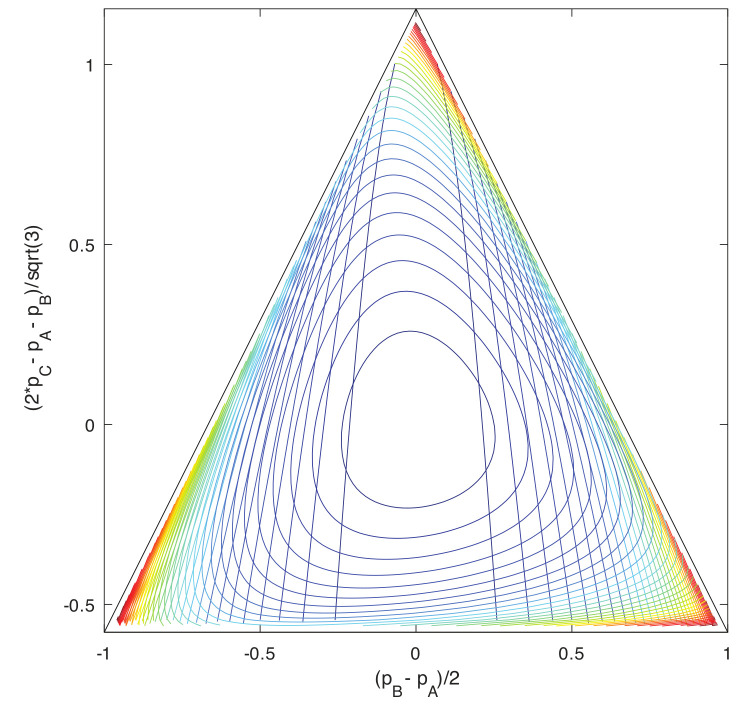
Simplex for a probability distribution $p=\left(p_{A},p_{B},p_{C}\right)$, with two coordinates $x_{AB}$ and $x_{ABC}$ of the form in Equation (56) on a 2-cycle and a 3-cycle.

Figure 3 shows examples of the coordinates (56) for a 2-cycle and a 3-cycle in the simplex of normalized activities $\psi_{i}/\sum_{j}\psi_{j}$ for three species $i\in \left\{A,B,C\right\}$.

#### 5.1.2. Vorticity in the Flowfield of Stationary Trajectories

Recall (Table 3) that detailed balance means ${\hat{A}}^{T}=\hat{A}$. In this case, Equation (51) implies equal and opposite change of ${\underline{S}}_{\mathrm{eff}}$ along relaxations and escapes, because the two trajectories are time-reverses of each other. Detailed balance thus generalizes the time-reversal symmetry of 1-dimensional systems to any number of dimensions, and is a correspondingly restrictive condition. This is the assumption in classical thermodynamics that identifies the Lyapunov and large-deviation roles of the entropy state function.

Already for complex-balanced CRNs, exact time reversal is generally broken. The gradients of the tangent vectors to relaxation and escape trajectories, with respect to the exponential-family coordinates θ in Equation (50), evaluate to

(58) $$\begin{array}{cc} \frac{\partial {\left({\dot{n}}_{\mathrm{rel}}\right)}_{p}}{\partial \theta_{q}} & =Y_{p}\hat{A}\mathrm{diag}\left[\psi \;\left(e^{\theta }\right)\right]Y_{q}^{T}\\ \frac{\partial {\left({\dot{n}}_{\mathrm{esc}}\right)}_{p}}{\partial \theta_{q}} & =-Y_{p}{\hat{A}}^{T}\mathrm{diag}\left[\psi \;\left(e^{\theta }\right)\right]Y_{q}^{T}. \end{array}$$

A measure of the time-asymmetry of the CRN is the vorticity, defined as the antisymmetric part of the matrices (58). This vorticity equals the gradient of the sum of tangent vectors to relaxations and escapes at the same point,

(59) $$\begin{array}{cc} \frac{\partial {\left({\dot{n}}_{\mathrm{esc}}+{\dot{n}}_{\mathrm{rel}}\right)}_{p}}{\partial \theta_{q}} & =Y_{p}\left(\hat{A}-{\hat{A}}^{T}\right)\mathrm{diag}\left[\psi \;\left(e^{\theta }\right)\right]Y_{q}^{T}. \end{array}$$

At the fixed point where θ=0, the vorticity is the antisymmetric part of the matrix $YAY^{T}$.

#### 5.1.3. Hyperflow Decomposition for Non-Complex-Balanced CRNs

For complex-balanced CRNs, $\hat{A}$ describes flows on the complex network, which is an ordinary directed graph. For non-complex-balanced flows, $Y\hat{A}1=0$, but A1≠0. $\hat{A}$ therefore cannot be written as a sum of adjacency matrices for currents on cycles in the complex graph. However, because net current still equals zero for every species, a basis for the currents at any fixed point still exists in *balanced hyperflows*. If the elements of *Y* are all integer values (as they are for chemistry or for biological population processes), the basis elements are *balanced integer hyperflows* [77]. Each such flow is an assignment of a set of non-negative integers to every current with nonzero $k_{ji}$, such that the net currents at each species vanish.

We may continue to refer to basis hyperflows with index α by extension of the complex-balanced case, and for each basis element there is a non-negative current ${\bar{ȷ}}_{\alpha }$ which is the coefficient of that flow in the stationary-state current solution. Integer hyperflows cannot be used to express ${\dot{\underline{S}}}_{\mathrm{eff}}$ as sums of KL divergences with uniform sign, in keeping with the variety of complications that arise for ${\dot{\underline{S}}}_{\mathrm{eff}}$ in non-complex-balanced CRNs.

### 5.2. Cycle Decomposition in the Microstate Space, and Non-Decrease of Relative Entropy Components

Next we apply a cycle decomposition similar to the one in the previous section, but in the microstate space rather than on the hypergraph, to prove non-negativity of the first two terms in Equation (11). The crucial distinction between the two levels is that balanced integer flows can always be mapped to cycles in the state space. For cycles in the hypergraph, a natural map is unique; for more general flows many mappings may be possible. Relative entropies thus give Lyapunov functions more generally for distributions over microstates than for macrostate variables. However, unlike the hypergraph where basis decompositions are finite, in the state space they generally require solution for infinitely many cycle currents and are difficult except in special cases.

#### 5.2.1. The System-Marginal Relative Entropy from ${\bar{\rho }}^{s}$

The system *s* and environment *e* must now be considered explicitly because housekeeping heat is an embedding of *s* in s⊗e. We therefore work in system indices $\left\{n_{s}\right\}$ and descale with the steady state ${\bar{\rho }}^{s}$ at the instantaneous $\rho^{e|s}$. In the following derivations $\rho^{s}$ and $\rho^{e|s}$ can be any normalizable distributions; macrostates are no longer singled out. The time-change of $D\;\left(\rho^{s}\|{\bar{\rho }}^{s}\right)$, fixing ${\bar{\rho }}^{s}$ as before, follows from Equation (1) and the form (40), as

(60) $$\begin{array}{cc} \dot{D}\;\left(\rho^{s}\|{\bar{\rho }}^{s}\right) & =\sum_{n_{s}}\log\left(\frac{\rho_{n_{s}}^{s}}{{\bar{\rho }}_{n_{s}}^{s}}\right){\dot{\rho }}_{n_{s}}^{s}\\ \; & =\sum_{n_{s}}\log\left(\frac{\rho_{n_{s}}^{s}}{{\bar{\rho }}_{n_{s}}^{s}}\right)\psi^{sT}\;\left(e^{-\partial /\partial n_{s}}\right)A^{s}\mathrm{diag}\left[\psi^{s}\;\left(e^{\partial /\partial n_{s}}\right)\right]\Psi^{s}\;\left(n_{s}\right)\rho_{n_{s}}^{s}\\ \; & =\sum_{n_{s}^{\prime }n_{s}}\log\left(\frac{\rho_{n_{s}^{\prime }}^{s}}{{\bar{\rho }}_{n_{s}^{\prime }}^{s}}\right)\cdot {\hat{T}}_{n_{s}^{\prime },n_{s}}^{s}\left(\frac{\rho_{n_{s}}^{s}}{{\bar{\rho }}_{n_{s}}^{s}}\right). \end{array}$$

From the definition (40), $1^{T}{\hat{T}}^{s}=0$ and ${\hat{T}}^{s}1=0$, as was the case for complex-balanced $A^{s}$. Therefore, ${\hat{T}}^{s}$ can be written as a sum of adjacency matrices for cycles, weighted by the currents around those cycles in the steady state. For a general CRN, it would not be assured that these cycles were all of finite length or that there were only finitely many of them passing through any given state $n_{s}$. However, for a CRN in which both the dimensions of A and *Y* and their matrix elements are finite, the integer hyperflow decomposition under $A^{s}$ and $Y^{s}$ is in turn finite, and the basis flows can be embedded in the microstate space to give a decomposition of ${\hat{T}}^{s}$.

Let α index any basis of integer hyperflows spanning kerYA. The cyclic flows spanning kerA embed without ambiguity in the state space, as images the cycles on the complex network. Each cycle α defines a sequence of state shifts $\left\{n\to n+Y_{j}-Y_{i}\right\}$ when the transitions $\left\{ji|\alpha \right\}$ in the cycle are activated in order.

Non-complex-balanced integer flows also create cycles through states, because by construction the sum of stoichiometric shifts in a balanced flow is zero. However, there may be a choice in the way a given flow projects into the state space, depending on the *realization*, defined as the order in which the reaction events in the flow are activated. Any order is acceptable, as long as each state in the cycle can be reached by a reaction that can execute. (This condition is called *reachability*. It can be violated if there are boundary states with too few individuals to permit the input complex to some reaction to be formed. States may be grouped into equivalence classes under reachability, as a means to study network features such as autocatalysis. Reachability is beyond the scope of this paper. For the large-population limits to which large-deviation scaling applies, under finitely generated reactions, all states will have arbitrarily many members and will be equivalent under reachability, so all embeddings of a finite integer hyperflow will always complete.) We then extend the indexing α from the images of the complex-balanced cycles to include all the integer flows.

Once a cycle realization has been chosen for each integer hyperflow, an embedding of these flows in the state space is defined as follows. Pick an arbitrary starting complex in the cycle, and for each $n_{s}$, embed an image of the cycle with the starting complex sampled at $n_{s}$. Then every state is passed through by every cycle with each complex in the cycle sampling from that state exactly once. Every link has a finite number of cycles passing through it, because the number of integer flows spanning kerYA and the length of each flow are both finite.

A set of currents $\left\{{\bar{ȷ}}_{\alpha ,n}\right\}$ that sum to the steady-state currents over each transition is then assigned to the cycles, indexed by the state n where cycle α samples at its starting complex. Solving for the values of the $\left\{{\bar{ȷ}}_{\alpha ,n}\right\}$ is of course equivalent to solving for $\bar{\rho }$, and is not generally a finite problem.

With these notations in place, Equation (60) becomes

(61) $$\begin{array}{cc} \dot{D}\;\left(\rho^{s}\|{\bar{\rho }}^{s}\right) & =-\sum_{n_{s}}\sum_{\alpha }{\bar{ȷ}}_{\alpha ,n_{s}}^{s}\sum_{n_{s}^{\prime }n_{s}|\alpha }\left(\frac{\rho_{n_{s}}^{s}}{{\bar{\rho }}_{n_{s}}^{s}}\right)\log\left(\frac{\rho_{n_{s}}^{s}/{\bar{\rho }}_{n_{s}}^{s}}{\rho_{n_{s}^{\prime }}^{s}/{\bar{\rho }}_{n_{s}^{\prime }}^{s}}\right). \end{array}$$

Equation (61) is a non-positive sum of KL divergences of probability ratios $\left(\rho_{n_{s}}^{s}/{\bar{\rho }}_{n_{s}}^{s}\right)$ referenced to their own values advanced around cycles, of the same form as Equation (53) for complex activities $e^{\theta Y_{i}}$ for macrostates on the hypergraph. The state space divergence involves an additional sum over the reference state $n_{s}$. As a result that the sum over α is finite, the contribution at each $n_{s}$ is finite and proportional by finite factors to ${\bar{\rho }}_{n_{s}^{\prime }}^{s}$ within a finite neighborhood of $n_{s}$. Therefore, the sum (61) is finite. This proves that the first line of Equation (11) is non-decreasing.

#### 5.2.2. Non-Negativity of the Housekeeping Entropy Rate

The cycle decomposition on the state space may also be used to prove non-negativity of the housekeeping entropy rate (10), by what amounts to a generating-function method similar to those in [51,52]. Here, rather than work with entropy changes over extended-time paths, we prove positivity moment by moment from the master equation.

Unlike the relative entropy within the system, the housekeeping entropy rate is not only a function of aggregated within-system transition rates $w_{n_{s}n_{s}^{\prime }}^{s}$, but must be disaggregated to all distinct system-environment interactions. Begin by expressing the sum of all transition currents both in terms of elementary events and in the previous cycle decomposition:

(62) $$\begin{array}{cc} J_{\rho }^{s} & \equiv \sum_{n_{s}}\rho_{n_{s}}^{s}\sum_{n_{s}^{\prime }\neq n_{s}}\sum_{n|n_{s}}\sum_{n^{\prime }|n_{s}^{\prime }}\sum_{ji\;|\;y_{j}-y_{i}=n^{\prime }-n}w_{n^{\prime }n}^{\left(ji\right)}\rho_{n}^{e|s}\\ \; & \equiv \sum_{n_{s}}\rho_{n_{s}}^{s}\sum_{n_{s}^{\prime }\neq n_{s}}w_{n_{s}^{\prime }n_{s}}^{s}\\ \; & =\sum_{n_{s}}\sum_{\alpha }{\bar{ȷ}}_{\alpha ,n_{s}}^{s}\sum_{n_{s}^{\prime }n_{s}|\alpha }\left(\frac{\rho_{n_{s}}^{s}}{{\bar{\rho }}_{n_{s}}^{s}}\right), \end{array}$$

where $w_{n^{\prime }n}^{\left(ji\right)}$ is labeled by the particular reaction connecting n and $n^{\prime }$. (For notational simplicity, we suppose that each ordered pair of complexes $\left(ji\right)$ is connected by at most one reaction. This assumption still allows a common difference vector $Y_{j}-Y_{i}$ to be mediated by several distinct pairs $\left(ji\right)$, and the generalization to more complex cases is straightforward.)

We follow the construction of correlation functions for a counting process from [53], for a quantity *r* with event-dependent values $r^{\left(ji\right)}$ defined as

(63) $$\begin{array}{cc} r_{n^{\prime }n}^{\left(ji\right)} & \equiv \log\left(\frac{w_{n^{\prime }n}^{\left(ji\right)}{\bar{\rho }}_{n_{s}}^{s}\rho_{n}^{e|s}}{w_{nn^{\prime }}^{\left(ji\right)}{\bar{\rho }}_{n_{s}^{\prime }}^{s}\rho_{n^{\prime }}^{e|s}}\right). \end{array}$$

A total housekeeping entropy rate denoted ${\dot{S}}^{\mathrm{HK}}$ is a sum over pairs of system states, of the quantity in Equation (10). Written as an expectation of *r*, it is

(64) $$\begin{array}{cc} {\dot{S}}^{\mathrm{HK}} & \equiv \sum_{n_{s}}\sum_{n_{s}^{\prime }\neq n_{s}}\sigma_{n_{s}n_{s}^{\prime }}^{\mathrm{HK}}w_{n_{s}n_{s}^{\prime }}^{s}\\ \; & =\sum_{n_{s}}\sum_{\alpha }{\bar{ȷ}}_{\alpha ,n_{s}}^{s}\sum_{n_{s}^{\prime }n_{s}|\alpha }\left(\frac{\rho_{n}^{s}}{{\bar{\rho }}_{n}^{s}}\right)\frac{1}{w_{n_{s}^{\prime }n_{s}}^{s}}\sum_{n|n_{s}}\sum_{n^{\prime }|n_{s}^{\prime }}\sum_{ji\;|\;y_{j}-y_{i}=n^{\prime }-n}w_{n^{\prime }n}^{\left(ji\right)}\rho_{n}^{e|s}r_{n^{\prime }n}^{\left(ji\right)}. \end{array}$$

The sign of Equation (64) can be deduced from convexity of the observable $e^{-r}$, as in the usual fluctuation theorems. *r* is nonzero only on transitions, the terms in the sum in Equation (62). Let dt be a short time interval. Then the expectation of any function of *r* on the interval will be $\left(1-dt\;J_{\rho }^{s}\right)$ times its value in an unchanging state, plus a term ∝dt from transitions. Extracting the contribution ∝dt for expectations of 1 and $e^{-r}$ over the interval, denoted ${\langle \rangle }_{\mathrm{dt}}$, gives

(65) $$\begin{array}{cc} {\langle 1\rangle }_{\mathrm{dt}}-\left(1-dt\;J_{\rho }^{s}\right) & =dt\sum_{n_{s}}\sum_{\alpha }{\bar{ȷ}}_{\alpha ,n_{s}}\sum_{n_{s}^{\prime }n_{s}|\alpha }\left(\frac{\rho_{n_{s}}^{s}}{{\bar{\rho }}_{n_{s}}^{s}}\right)\\ {\langle e^{-r}\rangle }_{\mathrm{dt}}-\left(1-dt\;J_{\rho }^{s}\right) & =dt\sum_{n_{s}}\left(\frac{\rho_{n_{s}}^{s}}{{\bar{\rho }}_{n_{s}}^{s}}\right)\sum_{n_{s}^{\prime }\neq n_{s}}\sum_{n|n_{s}}\sum_{n^{\prime }|n_{s}^{\prime }}\sum_{ji\;|\;y_{j}-y_{i}=n^{\prime }-n}w_{n^{\prime }n}^{\left(ji\right)}\rho_{n}^{e|s}{\bar{\rho }}_{n_{s}}^{s}e^{-r_{n^{\prime }n}^{\left(ji\right)}}\\ \; & =dt\sum_{n_{s}}\left(\frac{\rho_{n_{s}}^{s}}{{\bar{\rho }}_{n_{s}}^{s}}\right)\sum_{n_{s}^{\prime }\neq n_{s}}\sum_{n|n_{s}}\sum_{n^{\prime }|n_{s}^{\prime }}\sum_{ji\;|\;y_{j}-y_{i}=n^{\prime }-n}w_{nn^{\prime }}^{\left(ji\right)}\rho_{n^{\prime }}^{e|s}{\bar{\rho }}_{n_{s}^{\prime }}^{s}\\ \; & =dt\sum_{n_{s}}\left(\frac{\rho_{n_{s}}^{s}}{{\bar{\rho }}_{n_{s}}^{s}}\right)\sum_{n_{s}^{\prime }\neq n_{s}}w_{n_{s}n_{s}^{\prime }}^{s}{\bar{\rho }}_{n_{s}^{\prime }}^{s}\\ \; & =dt\sum_{n_{s}}\sum_{\alpha }{\bar{ȷ}}_{\alpha ,n_{s}}\sum_{n_{s}^{\prime }n_{s}|\alpha }\left(\frac{\rho_{n_{s}^{\prime }}^{s}}{{\bar{\rho }}_{n_{s}^{\prime }}^{s}}\right). \end{array}$$

Between the third and fourth lines in the evaluation of ${\langle e^{-r}\rangle }_{\mathrm{dt}}$, index labels n and $n^{\prime }$ are switched. As a result that the steady state currents decompose into cycles, whether $\left(\rho /\bar{\rho }\right)$ is summed over the first index or over the second index along a cycle, the sum is the same. Hence, the two expressions in Equation (65) are the same. By Jensen’s inequality, $d{\langle r\rangle }_{\mathrm{dt}}/dt={\dot{S}}^{\mathrm{HK}}\geq 0$, proving that Equation (64), which is the second line of Equation (11), is non-decreasing.

**Remark** **1.** *The Hatano–Sasa generating function is constructed to replace the transition matrix $\hat{T}$ with its adjoint [53]. In Equation (65), this relation is reflected in the way ${\langle e^{-r}\rangle }_{\mathrm{dt}}$ switches $\left(\rho /\bar{\rho }\right)$ to the tail position of links, whereas in ${\langle 1\rangle }_{\mathrm{dt}}$ it is in the head position. Exactly the same sum arises if positivity of $\dot{D}\;\left(\rho^{s}\|{\bar{\rho }}^{s}\right)$ is proved by a similar convexity argument, from the expectation of $\left(\rho_{n_{s}^{\prime }}^{s}/{\bar{\rho }}_{n_{s}^{\prime }}^{s}\right)/\left(\rho_{n_{s}}^{s}/{\bar{\rho }}_{n_{s}}^{s}\right)$ over an interval dt, which is the inverse exponential of the log-ratio of Hartley information appearing in Equation (61). Thus the two fluctuation theorems are for generating functions spanned by the same chord between two probability measures, though the counting observables in the two generating functions are distinct.*

## 6. Examples

### 6.1. Dualizing the Housekeeping Embedding Thermodynamics

In the first example, the system *s* is elementary: a 2-state model of polymerization and hydrolysis. Our interest is in how the embedding of such a model in a 1-parameter family of environments is captured in the intensive and extensive thermodynamic parameters, when these produce identical distributions ${\bar{\rho }}^{s}$, but with differing housekeeping entropy rate.

The motivation for the model is a question from astrobiology: in how far can two environments be considered analogues simply because they produce similar distributions for a material considered to be of biological interest. For instance, is Titan an analogue to early Earth if both are believed to support significant polymerization of small organic molecules [112,113,114], even if polymers on Titan are stable and near equilibrium at low water activity, whereas Earth produced them (putatively) through a competition between ligation driven by disequilibrium leaving groups such as phosphates [115,116] or thioesters [117,118] and disequilibrium hydrolysis?

The polymerization/hydrolysis mechanism, though elementary, can be a foundation for more complex heterogeneous polymerization models [85], in which polymers once formed may be sensitive to other consequences of water activity, such as propensity to fold or to aggregate. We ask, to what extent can the rate balance that governs polymerization, and water activity *per se*, be decoupled as measures of environmental similarity. The example will show that in the limit where one driving buffer goes to zero concentration and a subset of reactions become strictly irreversible, the two parameters can be made independent.

In this example, all influences on what we should call thermodynamic order come from kinetics in buffered non-equilibrium settings. Total entropy production is uninformative because the entropy associated with the polymer distribution sits atop a housekeeping heat that can be varied freely. Even knowing that housekeeping heat depends on a difference of chemical potentials gives no information when that difference is taken to infinity in an irreversible limit. Thus, none of the conservation laws linking the model to microscopically reversible mechanics provide information beyond what is in the transition matrix. Yet the thermodynamics of the process retains a regular representation: dualization of the housekeeping heat introduces an intensive state variable that is just the regular current through the polymerization/hydrolysis cycle, even in the strictly irreversible limit.

#### 6.1.1. One System, Families of Environments

Elementary reactions:

For simplicity, we let environmental distributions $\rho^{e|s}$ be Poisson with large capacity as a model of chemostats, and omit them from the explicit notation. Half-reaction rate constants are denoted ${\hat{k}}_{i}$ and ${\hat{\bar{k}}}_{i}$, where *i* is a label.

The first reaction type in the model is reversible dehydrating polymerization and hydrolysis, with schema

(66) $$\begin{array}{cc} A & \overset{{\hat{k}}_{1}}{\underset{{\hat{\bar{k}}}_{1}}{⇌}}A^{*}+H_{2}O. \end{array}$$

*A* are monomers capable of some oriented bonding (as seen for peptide or nucleotide bonds), and we will refer to one side of the oriented link as the bond *donor* and the other as the *acceptor*. Unmarked *A* are monomers unligated at the donor site, which may be free monomers in solution, or monomers ligated at other sites in a polymer, and which we take to be buffered to unit activity. $A^{*}$ are monomers ligated at the donor site within polymers. n denotes the number of $A^{*}$, which will be the counting index and is the number of anhydrous bonds available to be hydrolyzed. These may cleave free monomers from the end of a chain, or my cleave a chain internally; the same rate is assumed for either. For the calculation in this example, the distribution of polymer sizes formed is not of interest, and we take n to be the system state, which changes under a Poisson process. Appendix A briefly relates the Poisson model to ensembles of distributions for linear polymers. Water is also buffered, at activity $a_{H_{2}O}$, which is varied as a control parameter across a family of environments.

A second process, involving only environment species to define a reference scale for chemical potentials, is hydrolysis of a phosphoanhydride bond, with schema

(67) $$\begin{array}{cc} P^{*}+H_{2}O & \overset{{\hat{k}}_{2}}{\underset{{\hat{\bar{k}}}_{2}}{⇌}}P. \end{array}$$

$P^{*}$ is a bound form with activity $a_{P^{*}}$, and P a hydrolyzed form, with activity $a_{P}$. (Equation (67) is a stand-in for a variety of phosphoryl group-transfer processes, some linear as shown and some higher-order in the hydrolyzed species [116]. For higher-order reactions, appropriate powers of the activity would replace $a_{P}$ in the expressions below, without otherwise changing the results.)

Monomer ligation driven by phosphate hydrolysis is the stoichiometrically coupled reaction

(68) $$\begin{array}{cc} A+P^{*} & \overset{{\hat{k}}_{3}}{\underset{{\hat{\bar{k}}}_{3}}{⇌}}A^{*}+P. \end{array}$$

**Figure 4:**
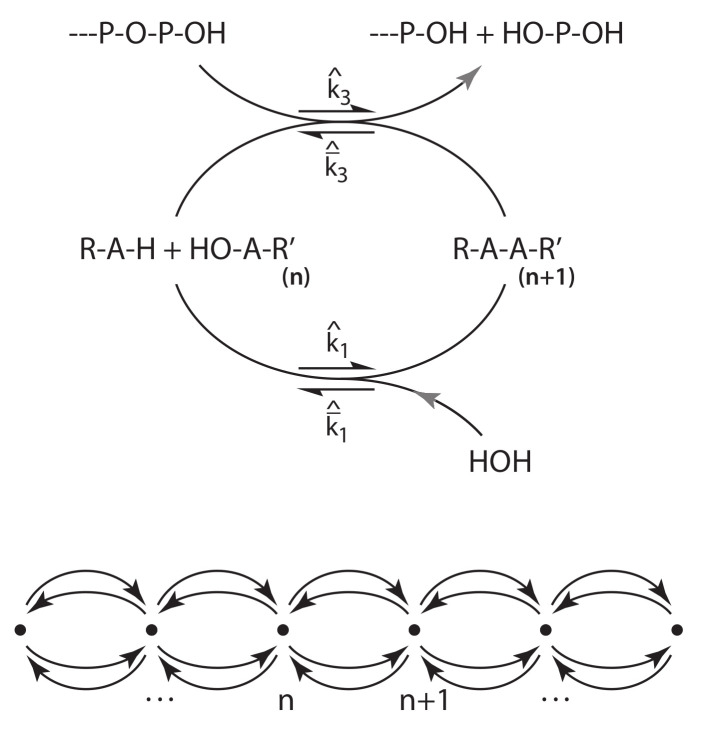
Illustration of the reaction schema (68). Species $P^{*}$ are modeled as anhydrous phosphates with any context beyond the ligating *P*. Monomers *A* could be hydroxyacids, amino acids, carbohydrates, or any other molecules with complementary H and OH groups subject to dehydrating condensation. *R* and $R^{\prime }$ are any ligands at sites other than the focal bond. Harpoons ⇌ remind that both reactions are bidirectional; grey arrows indicate net flux for driven polymerization. State space (shown below) is indexed by the number of hydrolizable *A*–*A* bonds n. Three independent cycles exist between any two adjacent states, in the cycle decomposition of Section 5.2 for non-equilibrium relaxation.

Equilibrium constants for the three reactions (66)–(68) are denoted ${\hat{K}}_{i}={\hat{k}}_{i}/{\hat{\bar{k}}}_{i}$. In actual chemistry, detailed balance implies the relation ${\hat{K}}_{3}={\hat{K}}_{1}{\hat{K}}_{2}$. To simplify notation, we will choose activity units to set ${\bar{k}}_{3}/{\bar{k}}_{1}=1$. Reaction 2 contributes only to dissipation internal to the environment (the third summand in Equation (11)), and we omit it, so the scale of ${\bar{k}}_{2}$ never enters. The system of coupled reactions (66) and (68), and the resulting state space for n, are diagrammed in Figure 4.

Poisson stationary distribution over polymer lengths:

The stationary distribution ${\bar{\rho }}^{s}$ for the number n of anhydrous *A*–*A* bonds under the joint action of reactions (66) and (68) is Poisson with parameter $K_{\mathrm{eff}}$, given by

(69) $$\begin{array}{cc} \frac{{\bar{\rho }}_{n+1}^{s}\left(n+1\right)}{{\bar{\rho }}_{n}^{s}} & \equiv K_{\mathrm{eff}}=\frac{{\hat{K}}_{1}+{\hat{K}}_{3}a_{P^{*}}}{a_{H_{2}O}+a_{P}}=\underline{n}. \end{array}$$

As shown in Equation (A7), the typical length of polymers thus formed is $\langle l\rangle =1+\underline{n}$.

The varieties of chemical disequilibrium:

We are interested in families of environments that share the same value of $K_{\mathrm{eff}}$ and so are indistinguishable from within the system. Chemical-potential coordinates within such a family are

(70) $$\begin{array}{cccc} \log\left(\frac{K_{\mathrm{eff}}a_{H_{2}O}}{{\hat{K}}_{1}}\right) & \equiv \mu_{H}\; & \log\left(\frac{{\hat{K}}_{2}a_{P^{*}}a_{H_{2}O}}{a_{P}}\right)\; & \equiv \mu_{P}. \end{array}$$

$\mu_{H}$ is the chemical potential for hydrolysis in the stationary polymer distribution. $\mu_{P}$ is the chemical potential of the phosphate system for hydrolysis.

Within surfaces of constant $K_{\mathrm{eff}}$ we consider variation of phosphate and water activities along contours of fixed $a_{P}/a_{H_{2}O}$, and vary this ratio later to define routes to irreversibility. ($a_{P^{*}}$ is a nonlinear function of the value of $a_{P}$ along this contour.) The thermodynamic equilibrium corresponds to $\mu_{P}=\mu_{H}=0$, or

(71) $$\begin{array}{cccc} {\hat{K}}_{2}\underline{a_{P^{*}}}\; & =\frac{a_{P}}{a_{H_{2}O}}\; & \frac{\underline{a_{H_{2}O}}}{{\hat{K}}_{1}}\; & =\frac{1}{K_{\mathrm{eff}}}. \end{array}$$

At equilibrium, the sub-system stationary distribution ${\bar{\rho }}^{s}$ is also the marginal ${\underline{\rho }}^{s}$ of the whole-system equilibrium; to emphasize that it is fixed as $\mu_{H}$ and $\mu_{P}$ are varied across a family of environments, we reference distributions to ${\underline{\rho }}^{s}$.

The activities governing reaction fluxes then depend on the coordinates (70) as

(72) $$\begin{array}{cccccc} a_{H_{2}O}\; & =\frac{{\hat{K}}_{1}}{K_{\mathrm{eff}}}e^{\mu_{H}}\; & a_{P}\; & =\frac{{\hat{K}}_{1}}{K_{\mathrm{eff}}}\frac{e^{\mu_{H}}\left(e^{\mu_{H}}-1\right)}{\left(e^{\mu_{P}}-e^{\mu_{H}}\right)}\; & a_{P^{*}}\; & =\frac{1}{{\hat{K}}_{2}}\frac{e^{\mu_{P}}\left(e^{\mu_{H}}-1\right)}{\left(e^{\mu_{P}}-e^{\mu_{H}}\right)}. \end{array}$$

Fixed $a_{P}/a_{H_{2}O}$ contours near and far from equilibrium satisfy

(73) $$\begin{array}{cc} \frac{\mu_{P}-\mu_{H}}{\mu_{H}}\; & \underset{\mu_{P}\to 0}{\to }\frac{a_{H_{2}O}}{a_{P}}\\ \mu_{P}-\mu_{H}\; & \underset{\mu_{P}\to \infty }{\to }\log\left(\frac{a_{H_{2}O}}{a_{P}}\right). \end{array}$$

Cycle decomposition of steady-state currents:

If environmental marginals $\rho^{e|s}$ are chemostats at the indicated chemical potentials, then currents in the stationary system distribution at a state n are proportional to ${\underline{\rho }}_{n}^{s}$ by factors that do not depend on n, and can be decomposed into three “specific currents” around cycles, which are functions of the chemostat activities

(74) $$\begin{array}{cc} {\hat{ȷ}}_{1} & =\frac{{\hat{K}}_{1}}{2}\left(e^{\mu_{H}}+1\right)\\ {\hat{ȷ}}_{3} & =\frac{{\hat{K}}_{1}}{2}\left(\frac{e^{\mu_{H}}-1}{e^{\mu_{P}}-e^{\mu_{H}}}\right)\left(e^{\mu_{P}}+e^{\mu_{H}}\right)\\ {\hat{ȷ}}_{\delta } & ={\hat{K}}_{1}\left(e^{\mu_{H}}-1\right). \end{array}$$

${\hat{ȷ}}_{1}$ is the average of forward and reverse currents in reaction (66), and ${\hat{ȷ}}_{3}$ the average of forward and reverse currents in reaction (68), divided by ${\underline{\rho }}_{n}^{s}$. ${\hat{ȷ}}_{\delta }$ is the difference of forward and reverse currents, which must be equal and opposite in reactions (66) and (68) in stationary state, also divided by ${\underline{\rho }}_{n}^{s}$; it is the only current in Schnakenberg’s *fundamental graph* [76] for this CRN. ${\hat{ȷ}}_{\delta }$ is so named because we have called it a “δ-flow” in [73,74]. The number of possible independent net flows in the stationary state of a CRN equals Feinberg’s [72] topological characteristic termed deficiency and denoted δ. The CRN of this example has δ=1. The fundamental graph omits currents ${\hat{ȷ}}_{1}$ and ${\hat{ȷ}}_{3}$ because they do not lead to dissipation in the stationary state, but they do for more general states.

#### 6.1.2. The Housekeeping Entropy Rate

The housekeeping entropy rate (64), for an arbitrary system distribution $\rho^{s}$, evaluates in terms of the specific currents (74) and the density ${\underline{\rho }}^{s}$, to

(75) S˙HK≡∑n∑n′≠nσnn′HKwnn′sρn′s=∑nρρ_n+1sȷ^1+ȷ^δ2μH−ȷ^3−ȷ^δ2μP−μH∑n+ρρ_nsȷ^3+ȷ^δ2μP−μH−ȷ^1−ȷ^δ2μHρ_ns∼ȷ^δμP+∫dnρ_ns∂∂nρρ_nsȷ^1μH+ȷ^3μH−μP.

The second expression is a continuum approximation to first order in derivatives, with $-\log\rho_{n}^{s}$ understood as usual to be approximated by the appropriate large-deviation function $S_{\mathrm{eff}}$.

To check that the exact (discrete) form of Equation (75) is positive semidefinite for arbitrary $\rho^{s}$, define measures

(76) $$\begin{array}{cccc} p & \equiv \frac{{\hat{ȷ}}_{1}-{\hat{ȷ}}_{\delta }/2}{{\hat{ȷ}}_{1}+{\hat{ȷ}}_{3}}\; & 1-p & \equiv \frac{{\hat{ȷ}}_{3}+{\hat{ȷ}}_{\delta }/2}{{\hat{ȷ}}_{1}+{\hat{ȷ}}_{3}}\\ q & \equiv \frac{{\hat{ȷ}}_{1}+{\hat{ȷ}}_{\delta }/2}{{\hat{ȷ}}_{1}+{\hat{ȷ}}_{3}}\; & 1-q & \equiv \frac{{\hat{ȷ}}_{3}-{\hat{ȷ}}_{\delta }/2}{{\hat{ȷ}}_{1}+{\hat{ȷ}}_{3}}. \end{array}$$

From the formulae (74), it follows that

(77) $$\begin{array}{cccc} \frac{p}{q} & =e^{-\mu_{H}} & \frac{1-p}{1-q} & =e^{\mu_{P}-\mu_{H}}, \end{array}$$

and thus Equation (75) can be written

(78) $$\begin{array}{cc} {\dot{S}}^{\mathrm{HK}} & =\sum_{n}{\underline{\rho }}_{n}^{s}\left({\hat{ȷ}}_{1}+{\hat{ȷ}}_{3}\right)\left[{\left(\frac{\rho }{\underline{\rho }}\right)}_{n+1}^{s}\;\;\;D\;\left(q\|p\right)+{\left(\frac{\rho }{\underline{\rho }}\right)}_{n}^{s}D\;\left(p\|q\right)\right], \end{array}$$

with $D\;\left(q\|p\right)$ a Kullback–Leibler divergence as elsewhere.

Housekeeping entropy rate as an embedding vector:

Equation (78) can be put in the form

(79) $$\begin{array}{cc} {\dot{S}}^{\mathrm{HK}} & =\sum_{n}\rho_{n}^{s}{\dot{\sigma }}_{n}^{\mathrm{HK}}. \end{array}$$

${\dot{\sigma }}^{\mathrm{HK}}\equiv \left[{\dot{\sigma }}_{n}^{\mathrm{HK}}\right]$ is a vector with positive-semidefinite components, which by Equation (77) equals zero only at $\mu_{P}=\mu_{H}=0$.

As noted, ${\bar{\rho }}^{s}$ is an extremal vector for the intrinsic relative entropy $-D\;\left(\rho^{s}\|{\bar{\rho }}^{s}\right)$ in the simplex of distributions $\rho^{s}$. By Equation (75), at this extremal point of *s*, $\sum_{n}{\bar{\rho }}_{n}^{s}{\dot{\sigma }}_{n}^{\mathrm{HK}}={\hat{ȷ}}_{\delta }\mu_{P}$.

#### 6.1.3. Legendre Duality for Housekeeping Entropy Rate

The chemical potential $\mu_{P}\to \infty$ if the activity $a_{P}\to 0$ at fixed $a_{P^{*}}$ and $a_{H_{2}O}$. In this limit, schema (68) becomes an irreversible reaction. Phenomenologically, all “thermodynamic” characteristics of the system remain regular, only the energetic accounting breaks down because it is referenced to the equilibrium state variable $\log a_{P}$, in a system that nowhere couples to an equilibrium environment.

The divergence of ${\dot{S}}^{\mathrm{HK}}$ in this limit is like the divergence of any extensive thermodynamic potential in the limit that one of the extensive state variables diverges, except that ${\dot{S}}^{\mathrm{HK}}$ is an inherently non-equilibrium potential, and $\mu_{P}$ only behaves like an extensive variable with respect to dissipation. (Note that ${\dot{S}}^{\mathrm{HK}}$ has no status with respect to equilibrium; while entropies are extensive, ${\dot{S}}^{\mathrm{HK}}$ depends inherently on a rate.)

From the view that thermodynamics is about statistical organization and not fundamentally about energy, it is natural to Legendre transform ${\dot{S}}^{\mathrm{HK}}$ to expose the dynamically intensive current that is dual to $\mu_{P}$. For dualization, we work within the tangent space to the family of constant $K_{\mathrm{eff}}$, but now vary $\mu_{P}$ independently of $\mu_{H}$, rather than varying within the non-linear contours (71) at fixed $a_{P}/a_{H_{2}O}$.

The gradient of ${\dot{S}}^{\mathrm{HK}}$ with respect to $\mu_{P}$ at fixed $\mu_{H}$ is

(80) ∂S˙HK∂μP=K^1∑nρ_nseμH−1eμP−eμHeμPρρ_ns−eμHρρ_n+1seμPeμHeμP+eμHK^1Keff+eμPeμHeμP+eμHρρ_n+1s−ρρ_n+1sμH−μP∼ȷ^δ−∫dnρ_ns∂∂nρρ_nsȷ^3+∂ȷ^3∂μPμP−μH.

By Equation (74), ${\hat{ȷ}}_{3}\left(\mu_{P}-\mu_{H}\right)$ is convex in $\mu_{P}$, so its derivative, the term in square brackets in the final line of Equation (80), is invertible to a value for $\mu_{P}$. The Legendre dual potential to ${\dot{S}}^{\mathrm{HK}}$ on $\mu_{P}$, which we denote ${\dot{F}}_{P}$, is then given by

(81) $$\begin{array}{cc} {\dot{F}}_{P}\;\left(\frac{\partial {\dot{S}}^{\mathrm{HK}}}{\partial \mu_{P}},\mu_{H}\right) & \equiv \mu_{P}\frac{\partial {\dot{S}}^{\mathrm{HK}}}{\partial \mu_{P}}-{\dot{S}}^{\mathrm{HK}}\\ \; & \approx -\int dn\;{\underline{\rho }}_{n}^{s}\frac{\partial }{\partial n}{\left(\frac{\rho }{\underline{\rho }}\right)}_{n}^{s}\left[\frac{\partial {\hat{ȷ}}_{3}}{\partial \mu_{P}}\mu_{P}\left(\mu_{P}-\mu_{H}\right)+\left({\hat{ȷ}}_{3}+{\hat{ȷ}}_{1}\right)\mu_{H}\right]. \end{array}$$

Independent variation of control parameters in the irreversible limit:

We may now consider the effects of varying $\mu_{H}$, which remains finite, across the one-parameter family of contours of different $a_{P}/a_{H_{2}O}$ as $a_{P}\to 0$. $\left({\hat{ȷ}}_{3}+{\hat{ȷ}}_{1}\right)$ approaches a constant independent of $\mu_{P}$, and the only term in Equation (81) including multiples of diverging $\mu_{P}$ evaluates to

(82) $$\begin{array}{ccc} & -\frac{\partial {\hat{ȷ}}_{3}}{\partial \mu_{P}}\mu_{P}\left(\mu_{P}-\mu_{H}\right)\to & \\ \; & \to {\hat{K}}_{1}\left(\frac{a_{P}+a_{H_{2}O}}{a_{H_{2}O}}\right)\mu_{H}\left[1-\frac{1}{3!}{\left(\frac{a_{H_{2}O}}{a_{P}}\right)}^{2}\mu_{H}^{2}\right]\; & :\;\mu_{H}\to 0\\ \; & \to 2{\hat{ȷ}}_{3}e^{-\left({\hat{K}}_{1}/K_{\mathrm{eff}}a_{P}\right)\mu_{H}}\mu_{P}\left(\mu_{P}-\mu_{H}\right)\; & :\;\mu_{H}≳1. \end{array}$$

It approaches ${\hat{K}}_{1}\mu_{H}$ within a range around $\mu_{H}=0$ that becomes vanishingly small as $a_{P}\to 0$, and converges exponentially to zero outside that range.

Thus, the susceptibility that is the Legendre dual to $\mu_{P}$, $\partial {\dot{S}}^{\mathrm{HK}}/\partial \mu_{P}\to {\hat{ȷ}}_{\delta }$, a function only of $\mu_{H}$, almost everywhere. The potential ${\dot{F}}_{P}$ retains only the $\mu_{P}$-independent terms in Equation (81). If, for example, we choose $\rho^{s}$, a macrostate with mean $\bar{n}$, then $\int dn\;{\underline{\rho }}_{n}^{s}\partial {\left(\rho /\underline{\rho }\right)}_{n}^{s}/\partial n=\left(\bar{n}-\underline{n}\right)$ and ${\dot{F}}_{P}\to -\left(\bar{n}-\underline{n}\right)\left({\hat{ȷ}}_{3}+{\hat{ȷ}}_{1}\right)\mu_{H}$.

In the irreversible limit the problem is seen to separate. ${\bar{\rho }}^{s}$ dictates the intrinsic thermodynamics of the polymer system, while along a contour that fixes $K_{\mathrm{eff}}$, the dual potential ${\dot{F}}_{P}$ describes the non-trivial dependence of entropy change in the environment on $\rho^{s}$. ${\dot{S}}^{\mathrm{HK}}$ diverges harmlessly as an extensively-scaling potential independent of $\rho^{s}$ with a regular susceptibility ${\hat{ȷ}}_{\delta }$.

### 6.2. Metastability and ${\bar{\rho }}^{s}$ as the Reference Measure for Relative Entropy

The second example uses the same buffered driving environment as the first, but separates phosphate-driven ligation into a distinct, self-catalyzed channel while leaving the reaction (66) with water uncatalyzed. The model for system catalysis is a variant on the cubic Schlögl model of Figure 2. It is chosen to highlight the difference between the entropy rate (8) typically assigned as “environmental” and the housekeeping entropy rates (10). A quantity ${\dot{S}}^{\mathrm{env}}$ is a function only of the realized distributions $\rho^{s}$ and $\rho^{e|s}$. Nowhere does it reflect the joint participation of uncatalyzed and catalyzed reactions within the same system.

The difference ${\dot{S}}^{\mathrm{HK}}-{\dot{S}}^{\mathrm{env}}$ may have either sign. We illustrate the case where phosphate activities are chosen to put the system in its bistable regime, and $\mu_{P}$ is chosen to make the unstable root $n_{2}$ of the rate Equation (47) a fixed point. For equivalent rate constants a Poisson distribution $\rho^{s}$ at mean $n_{2}$ would be the driven stationary distribution in the previous example, and the ${\dot{S}}^{\mathrm{env}}$ is the same in the two models. However, ${\dot{S}}^{\mathrm{HK}}-{\dot{S}}^{\mathrm{env}}<0$ because the loss of large-deviation accessibility in the system results not only from its Shannon entropy change, but from the measure of that entropy relative to the true, bistable stationary distribution ${\bar{\rho }}^{s}$.

The autocatalytic model replaces the schema (68) with

(83) $$\begin{array}{cc} 2A^{*}+A+P^{*} & \overset{{\hat{k}}_{4}}{\underset{{\hat{\bar{k}}}_{4}}{⇌}}3A^{*}+P. \end{array}$$

Catalysis does not change energetics, so the rate constants must satisfy ${\hat{K}}_{4}={\hat{K}}_{1}{\hat{K}}_{2}$.

In the mass-action rate law (47), the roots $n_{1}<n_{2}<n_{3}$ and $n_{2}$ is unstable. We set a characteristic scale for transitions between water and phosphate control by choosing relative rate constants ${\bar{k}}_{4}/{\bar{k}}_{1}=\;1/n_{2}^{2}$.

The Schlögl model is a birth–death process, so ${\bar{\rho }}^{s}$ is exactly solvable. In place of $K_{\mathrm{eff}}$ from Equation (69) adjacent indices are related by a position-dependent effective equilibrium constant

(84) $$\begin{array}{cc} \frac{{\bar{\rho }}_{n+1}^{s}\left(n+1\right)}{{\bar{\rho }}_{n}^{s}} & \equiv K\;\left(n\right)=\frac{{\hat{K}}_{1}+{\hat{K}}_{4}a_{P^{*}}\;n\left(n-1\right)/n_{2}^{2}}{a_{H_{2}O}+a_{P}\;n\left(n-1\right)/n_{2}^{2}}. \end{array}$$

Note that $K\;\left(n_{2}\right)\to K_{\mathrm{eff}}$ to integer rounding error, at all activity levels.

In terms of the same roots, choose water activity to set the lower limit of Equation (84) at n≤1 to

(85) $$\begin{array}{cc} \frac{{\hat{K}}_{1}}{a_{H_{2}O}}\; & =\frac{1}{\sum_{i}1/n_{i}}\equiv \underline{n}. \end{array}$$

We will vary the activities in the phosphorus system so that ${\bar{\rho }}^{s}$ moves from a lower stable mode near equilibrium, through a bistable phase, to an upper stable mode driven by phosphorylation farther from equilibrium.

The cubic Schlögl model has an appealing interpretation as a phenomenological model. Note from Appendix A that for a Poisson distribution with parameter *K* corresponding to a root $n_{i}$, the typical polymer length $\langle l\rangle =1+K$ by Equation (A7). If $K∼n_{1}$ characterizes polymers too short to fold, and $K∼n_{3}$ characterizes polymers in which folding with some catalytic consequence occurs with non-vanishing frequency $\propto {\langle l\rangle }^{2}$ suggesting self-interactions within a string, then the catalyzed channel for ligation first arises in proportion to the occurrence of folded polymers.

Equilibrium is again defined as in Equation (71), and there $K\;\left(n\right)\overset{\mathrm{eq}}{=}\underline{n}\;;\;\forall n$. The marginal distribution for *s* in the whole-system equilibrium, ${\underline{\rho }}^{s}$, is then Poisson with parameter $\underline{n}$. Recall that, as a solution in detailed balance, it does not and cannot reflect the fact that *s* is a CRN capable of bistability. To match rate constants to the roots $\left\{n_{1},n_{2},n_{3}\right\}$ of the Schlögl model, we define a reference equilibrium for the phosphorus system with activity levels at

(86) $$\begin{array}{cc} {\hat{K}}_{2}\underline{a_{P^{*}}}=\frac{\underline{a_{P}}}{a_{H_{2}O}}\; & =1. \end{array}$$

We now consider a family of driving environments in which the chemical potential $\mu_{P}$ is partitioned between the activities $a_{P^{*}}$ and $a_{P}$ in the proportions

(87) $$\begin{array}{cc} \log\left(\frac{a_{P^{*}}}{\underline{a_{P^{*}}}}\right) & =\frac{\log\left[\left(\sum_{i}n_{i}\right)n_{2}^{2}/\left(\prod_{i}n_{i}\right)\right]}{\log\left[\left(\sum_{i}n_{i}\right)\left(\sum_{i}1/n_{i}\right)\right]}\mu_{P}\\ \log\left(\frac{a_{P}}{\underline{a_{P}}}\right) & =-\frac{\log\left[\left(\prod_{i}n_{i}\right)\left(\sum_{i}1/n_{i}\right)/n_{2}^{2}\right]}{\log\left[\left(\sum_{i}n_{i}\right)\left(\sum_{i}1/n_{i}\right)\right]}\mu_{P}. \end{array}$$

The two potentials (87) are coordinates in an exponential family for the phosphorus system. Where $\mu_{P}=\log\left[\left(\sum_{i}n_{i}\right)\left(\sum_{i}1/n_{i}\right)\right]$, the three roots of the mass action law pass through the values $\left\{n_{1},n_{2},n_{3}\right\}$ from Figure 2.

The difference in relative dissipations between the driven steady state ${\bar{\rho }}^{s}$ and ${\underline{\rho }}^{s}$, at adjacent n indices, is given by the ratio of effective rate constants

(88) $$\begin{array}{cc} \log\left(\frac{{\bar{\rho }}_{n+1}^{s}/{\underline{\rho }}_{n+1}^{s}}{{\bar{\rho }}_{n}^{s}/{\underline{\rho }}_{n}^{s}}\right) & =\frac{K\;\left(n\right)}{\underline{n}}\\ \; & =\frac{1+\left(a_{P^{*}}/\underline{a_{P^{*}}}\right)n\left(n-1\right)/n_{2}^{2}}{1+\left(a_{P}/\underline{a_{P}}\right)n\left(n-1\right)/n_{2}^{2}}\\ \; & =\frac{1+e^{\mu_{P}}\left(a_{P}/\underline{a_{P}}\right)n\left(n-1\right)/n_{2}^{2}}{1+\left(a_{P}/\underline{a_{P}}\right)n\left(n-1\right)/n_{2}^{2}}. \end{array}$$

**Figure 5:**
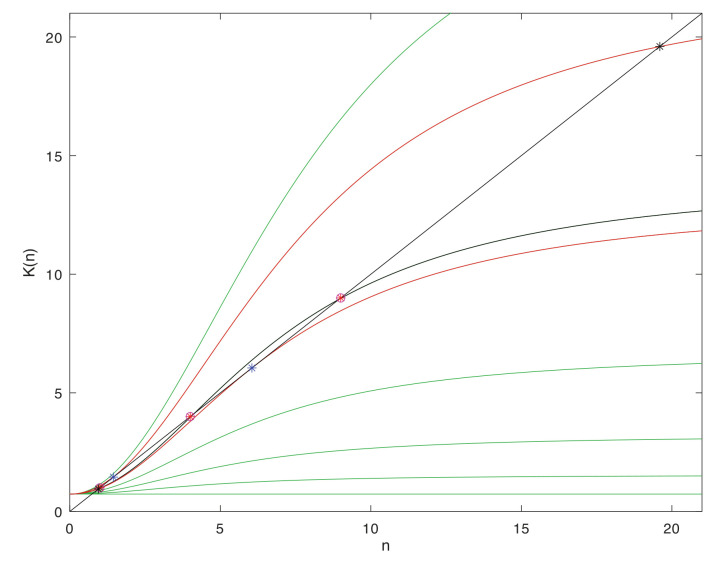
$K\;\left(n\right)$ on a sequence of increasing $\mu_{P}$ values with proportions (87). Curve at $\mu_{P}=\log\left[\left(\sum_{i}n_{i}\right)\left(\sum_{i}1/n_{i}\right)\right]$ is black. Boundaries of bistable range are red. Fixed points $K\;\left(n\right)=n$ are shown with markers.

Equation (88) is a sigmoidal function of $\log\left(n/n_{2}\right)$ in the range $K\;\left(n\right)/\underline{n}\in \left[1,e^{\mu_{P}}\right)$, graphed versus $\mu_{P}$ in Figure 5. The hydrolysis potential $\mu_{H}$ from Equation (70 for the linear system is replaced in the autocatalytic case by a position-dependent chemical potential

(89) $$\begin{array}{cc} \mu \;\left(n\right) & \equiv \log\left(\frac{a_{H_{2}O}{\bar{\rho }}_{n+1}^{s}\left(n+1\right)}{{\hat{K}}_{1}{\bar{\rho }}_{n}^{s}}\right)=\log\left(\frac{K\;\left(n\right)}{\underline{n}}\right). \end{array}$$

In the cycle decomposition, the specific currents exiting and entering a site n, now functions of n, depend on activities as

(90) $$\begin{array}{cc} {\hat{ȷ}}_{1} & =\frac{1}{2}\left(K\;\left(n\right)a_{H_{2}O}+{\hat{K}}_{1}\right)\\ {\hat{ȷ}}_{3} & =\frac{1}{2}\frac{n\left(n-1\right)}{n_{2}^{2}}\left({\hat{K}}_{4}a_{P^{*}}+K\;\left(n\right)a_{P}\right)\\ {\hat{ȷ}}_{\delta } & =K\;\left(n\right)a_{H_{2}O}-{\hat{K}}_{1}\\ \; & =n\left(n-1\right)\left({\hat{K}}_{4}a_{P^{*}}-K\;\left(n\right)a_{P}\right). \end{array}$$

In the chemical-potential coordinates, these become

(91) $$\begin{array}{cc} {\hat{ȷ}}_{1} & =\frac{{\hat{K}}_{1}}{2}\left(e^{\mu \left(n\right)}+1\right)\\ {\hat{ȷ}}_{3} & =\frac{{\hat{K}}_{1}}{2}\frac{n\left(n-1\right)}{n_{2}^{2}}\left(e^{\mu_{P}}+e^{\mu \left(n\right)}\right)\frac{a_{P}}{\underline{a_{P}}}\\ {\hat{ȷ}}_{\delta } & ={\hat{K}}_{1}\left(e^{\mu \left(n\right)}-1\right)\\ \; & ={\hat{K}}_{1}\frac{n\left(n-1\right)}{n_{2}^{2}}\left(e^{\mu_{P}}-e^{\mu \left(n\right)}\right)\frac{a_{P}}{\underline{a_{P}}}. \end{array}$$

Positive-semidefiniteness of ${\dot{S}}^{\mathrm{HK}}$ again follows by using the ratios (76) to produce the form (78), where now instead of Equation (77), we have

(92) $$\begin{array}{cccc} \frac{p}{q} & =e^{-\mu \left(n\right)}\; & \frac{1-p}{1-q} & =e^{\mu_{P}-\mu \left(n\right)}. \end{array}$$

For this example, we separate out a term ${\dot{S}}^{\mathrm{env}}$, which is the sum over transitions of Equation (8), from ${\dot{S}}^{\mathrm{HK}}$, to give

(93) S˙HK=∑nK^1nn−1n22aP*aP*_ρns−aPaP_ρn+1sn+1n_μPρρ¯ns∑n+ȷ^1+ȷ^3ρn+1sn+1Kn−ρnsμn=∑nK^1nn−1n22ρ_nsaP*aP*_ρρ_ns−aPaP_ρρ_n+1sμPρρ¯ns∑n+ȷ^1+ȷ^3ρ¯nsρρ¯n+1s−ρρ¯nsμn=S˙env+∑nȷ^1+ȷ^3ρ¯nsρρ¯n+1s−ρρ¯nsμn.

${\dot{S}}^{\mathrm{env}}$ does not depend on ${\bar{\rho }}^{s}$, and differs from the sum of terms multiplying $\mu_{P}$ in Equation (75) only by the n-dependence now multiplying ${\hat{K}}_{1}$ in Equation (93).

**Figure 6:**
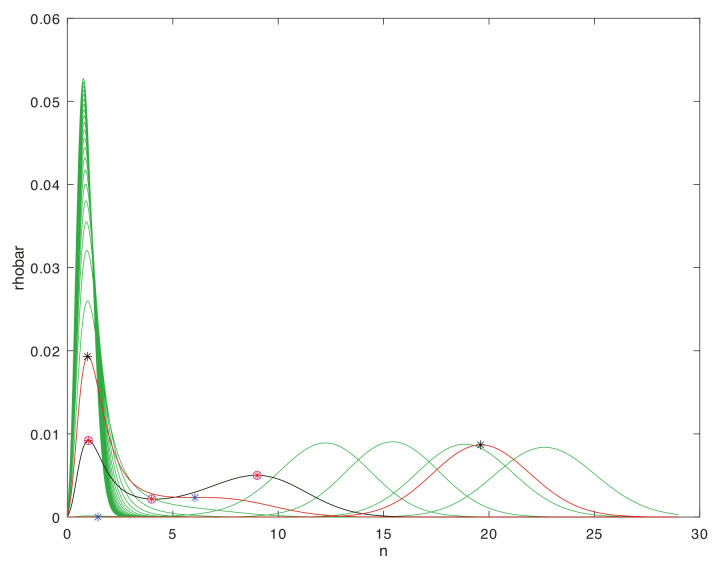
Densities ${\bar{\rho }}^{s}$ for a scale factor $4\times \left\{1,4,9\right\}$ for roots.

**Figure 7:**
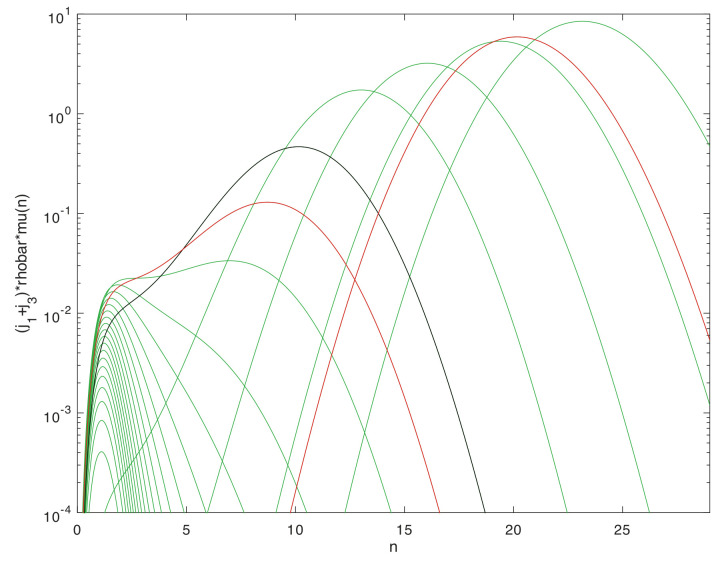
The combination $\left({\hat{ȷ}}_{1}+{\hat{ȷ}}_{3}\right){\bar{\rho }}_{n}^{s}\mu \;\left(n\right)$.

All dependence of ${\dot{S}}^{\mathrm{HK}}$ on ${\bar{\rho }}^{s}$ comes from the second term involving $\mu \;\left(n\right)$. The shape of ${\bar{\rho }}_{n}^{s}$ is shown as a function of $\mu_{P}$ in Figure 6, and the lumped coefficient $\left({\hat{ȷ}}_{1}+{\hat{ȷ}}_{3}\right){\bar{\rho }}_{n}^{s}\;\mu \;\left(n\right)$ multiplying the difference term in $\left(\rho^{s}/{\bar{\rho }}^{s}\right)$ in Equation (93) is shown in Figure 7.

As an example, consider the value $\mu_{P}=\log\left[\left(\sum_{i}n_{i}\right)\left(\sum_{i}1/n_{i}\right)\right]$ that makes $\left\{n_{1},n_{2},n_{3}\right\}$ the three fixed points, and evaluate the embedding entropies for a Poisson distribution $\rho^{s}$ with mean $n_{2}$, the unstable fixed point. The “environmental” component

(94) $$\begin{array}{cc} {\dot{S}}^{\mathrm{env}} & ={\hat{K}}_{1}\sum_{n}\rho_{n}^{s}\frac{n\left(n-1\right)}{n_{2}^{2}}\left(e^{\mu_{P}}-\frac{n_{2}}{\underline{n}}\right)\frac{a_{P}}{\underline{a_{P}}}\mu_{P}\\ \; & ={\hat{K}}_{1}\left(e^{\mu_{P}}-e^{\mu \left(n_{2}\right)}\right)\frac{a_{P}}{\underline{a_{P}}}\mu_{P}\\ \; & ={\hat{ȷ}}_{\delta }\;\left(n_{2}\right)\mu_{P}. \end{array}$$

is the same as the value ${\hat{ȷ}}_{\delta }\mu_{P}$ following from Equation (75) for the non-catalytic CRN, if we set $\mu_{H}=\log\left(n_{2}/\underline{n}\right)$ in Equation (70), where $K_{\mathrm{eff}}\to n_{2}$ and this $\rho^{s}$ is a driven steady state. It is also the value of ${\dot{S}}^{\mathrm{HK}}$ in Equation (79) at the extremal distribution $\rho^{s}={\bar{\rho }}^{s}$ for the linear model.

For the same $\rho^{s}$, recognizing that $\left(\mu \;\left(n\right)-\mu_{H}\right)=\log\left(K\;\left(n\right)/n_{2}\right)$, the gradient relative to the bistable reference distribution ${\bar{\rho }}^{s}$ in Equation (93) expands to ${\bar{\rho }}_{n}^{s}\left[{\left(\rho /\bar{\rho }\right)}_{n+1}^{s}-{\left(\rho /\bar{\rho }\right)}_{n}^{s}\right]\approx -\rho_{n}^{s}\left[\mu \;\left(n\right)-\mu_{H}\right]$. The difference of environment from housekeeping entropy changes then evaluates to

(95) $$\begin{array}{cc} {\dot{S}}^{\mathrm{HK}}-{\dot{S}}^{\mathrm{env}}\; & \approx -\sum_{n}\left({\hat{ȷ}}_{1}+{\hat{ȷ}}_{3}\right)\rho_{n}^{s}\mu \;\left(n\right)\left[\mu \;\left(n\right)-\mu_{H}\right]. \end{array}$$

The combination $\left({\hat{ȷ}}_{1}+{\hat{ȷ}}_{3}\right)\rho_{n}^{s}\mu \;\left(n\right)$ is increasing through $n_{2}$ (See Figure 7), and $\left[\mu \;\left(n\right)-\mu_{H}\right]$ is antisymmetric, so Equation (95) is negative.

Hence, not all of the entropy change by ${\dot{S}}^{\mathrm{env}}$ reflects a loss of large-deviation accessibility attributable to the environment. A quantity equal to ${\dot{S}}^{\mathrm{env}}-{\dot{S}}^{\mathrm{HK}}$ is assigned in the natural decomposition (11) to the system, because a Poisson distribution around $n_{2}$ is unstable and has excess net probability to relax toward the bistable distribution ${\bar{\rho }}^{s}$.

## 7. Discussion

### 7.1. The Problem of Macroworlds as an Alternative Central Contribution

A suggestion that thermodynamics should not be mainly about the relation between work and heat flows would have been non-sequitur at the turn of the 20th century. Not only were applications centered around interactions with a thermal system through mechanical boundaries [59]; the nature of irreversibility and dissipation as scientific problems was framed in their relation to mechanical principles of time-reversibility [119] and conservation of energy [26,120].

Innovations in scaling methods, however, in context of the unification of statistical and quantum theories in the second half of the 20th century, have exposed *understanding the existence of macroworlds* as one of the central problems in science. Section 3 explained how large-deviations scaling formalizes a concept of macrostate in terms of selected distributions over microstates. When methods derived for phase transitions and critical phenomena in condensed matter [36] were merged with the renormalization-group approach [35] to vacuum quantum field theory [38], it became clear that renormalization-group flow, a closely related operation of dimensional reduction to large-deviations scaling—through the correspondence of Keldysh to 2-field statistical field methods, the algebras of the two can often be interconverted [80,121]—provides a concept of *effective fields* describing the robust properties of macroworlds through phenomenological Lagrangians [37]. Effective fields are the counterparts under renormalization to classical states under large-deviations scaling.

In effective field theories nested through phase transitions (as in the models of this paper), microstates are assimilated back to macrostates. The result has been a theory of the vacuum and the hierarchy of matter in which all earlier, partly phenomenological concepts of elementary and composite objects and interactions has been subsumed within a semantics of distributions. While these physics examples continue to be organized by symmetries and conservation laws, similar scaling methods developed independently for reliable coding [45,66] offer non-mechanical counterparts (see [122], Ch. 7).

The alternative characterization offered, then, is that *thermodynamics is not fundamentally the theory of the movements of heat, but rather the theory of the emergence of macroworlds from microworlds.*

### 7.2. The End of Entropy Flow, the Natural Partition, and Hartley Information

In the absence of an energy-mediated constraint (adiabatic accessibility [32]) on the states jointly realizable by a system and its environment, the notion of “entropy flow” between subsystems may never arise. (Even in classical thermodynamics, it is only well-defined for adiabatic transformations, in which both subsystems remain in classical states constrained by energy at every moment.) The default description of entropy change ceases to be the metaphor of a fluid and becomes the literal one: it is the loss of large-deviation accessibility of classical states due to relaxation of the whole-system distribution. The concept of a tangent surface delimiting the amount of entropy gain in one component that can be influenced by the state of another, instead of the adiabatic transformation, is filled by the the vector of Hartley informations $-\log{\bar{\rho }}^{s}$ of the stationary state for the $T^{s}$ at that moment. The natural system-environment partition (11) encodes the conditional independence of components of the loss of states accessible by fluctuations.

Hartley information appears as a random variable along trajectories in the construction of the generating functional for housekeeping heat [5,52]. In standard treatments, its interpretation as an entropy is taken to depend on its association with a dissipated heat. (The exact statement, referring to Equation (123) in [5], is: “In the absence of a first law for the master equation dynamics, which would require further physical input not available at this general stage, this identification is by analogy only.”) Here, $-\log{\bar{\rho }}^{s}$ gains its interpretation as an entropy through its role as a carrier of information (an interpretation independent of dynamics advocated in [58]), about the interaction of processes within a system on its own limiting large-deviation function and on its capacity to limit large-deviations in the environment.

### 7.3. Making Trajectories First-Class Citizens

The persistence over 90 years of Onsager’s program [11,12] of making equilibrium Gibbs entropies the foundation of non-equilibrium thermodynamics has built an asymmetry into this concept even within physics, at the same time as growth in statistical methods for path ensembles has reduced the reason for an asymmetry to exist, by defining the same tools for paths as for states. The equilibrium Gibbs entropy is defined from the large-deviation function on ensembles of states. The stochastic effective action (25) [67] is the corresponding functional for trajectories. However, the role that energy conservation plays in stochastic thermodynamics, as a constraint on which states can be jointly occupied by components within a system and within its environment, will not generally have a counterpart for the jointly realizable trajectories involving these components. (Consider, as examples, non-Markovian noise sources and error-correcting codes exploiting time correlations to optimize against those environments [123].)

Borrowing a term from computer science, the privileged roles of equilibrium entropy and energy conservation in current non-equilibrium thermodynamics makes spaces of states “first-class citizens” [124], and admits thermodynamic interpretations for ensembles only in so far as those derive from equilibrium heat. Jaynes anticipated [125,126] a self-contained thermodynamic interpretation for path ensembles, though still only in a limited form referenced to the *calibers* of cross-sections of states. If there is to be a thermodynamics in which trajectories become first-class citizens on par with states, it will need a foundation in more general primitives such as large-deviation accessibility and separation of scales, as in the domain-agnostic framework presented here.

### 7.4. Rule-Based Systems and Life

This paper’s formula for a self-contained and substrate-agnostic thermodynamics is meant to support concept discovery in two domains that should clearly have such a thermodynamics, and for which energy conservation should play a limited role or no role at all for many interesting questions. (A well-known example of a constraint that is proved by a fluctuation theorem, but not binding because a more proximal constraint exists, is that of total entropy production versus the “excess heat” in a driven system. [127] The former bounds the non-equilibrium entropy change within a distribution, but is uninformative because it diverges on long timescales. The tight bound, under some conditions, is the finite excess heat.)

The first domain is rule-based modeling [110] which recognizes the finitary-to-infinitary mappings exhibited here between CRN generators and state spaces as a widely generalizable organizing principle. The algebra and combinatorics of non-commutative rule systems is a very active area of study [77,128,129,130,131] which connects to chemistry, systems biology, theories of algorithms, process calculus, and much more. A thermodynamics of rule-based systems that expands the conceptual scope of science will not be a reduction to their consequences for heat generation.

The second domain is the thermodynamic nature of life [122], encompassing rule-based order in its chemical substrate, the multi-level control systems and error correction required to maintain complex dynamical phases, and the nested population dynamics and historical contingency of evolution. There will be order in such systems that derives, sometimes cryptically, from microscopic time-reversibility. It is inconceivable that there will not be much more order, of novel kinds, that originates from the abundance of architectures—most of them irreversible—standing between the microscopic substrate and robust macrophenomena.

## 8. Concluding Remarks

The presentation above is meant to encapsulate a shift in emphasis that has been building, from questions about work and heat that were germane in mechanics and classical thermodynamics, to a concern with distinguishing sources of irreversibility in a large-deviation sense. That shift was enabled by the discovery of fluctuation theorems that identify the natural modularization of a system into a focal subsystem and its environmental context. The use here of the Hartley information defining the housekeeping entropy rate as a connection, rather than as a term in a potential function, is meant to provide a natural information-based interpretation in place of analogies of thermodynamic potentials to mechanical energy surfaces.

The derivation of all roles of entropy by suitable restrictions or conditionings of a single overall large-deviation function is meant to obviate formulations such as “violation of the 2nd law”, which turn on an overly coarse semantics and are unhelpful in separating different categories of probabilistic effects. Doing so has required, first and foremost, a systematic approach to the concept and construction of macrostates, and to the treatment of the associated entropy state functions. The decomposition in Equation (34) of $D\;\left(\rho^{\left(\bar{n}\right)}\|\underline{\rho }\right)$ in terms of the Lyapunov entropy state function $S_{\mathrm{eff}}$ and a remainder $D\;\left(1_{\underline{n}\left(\bar{n}\right)}\|\underline{\rho }\right)$ that must be relaxed by instantons, retains an un-violated 2nd law for relative entropy, and also the Lyapunov and large-deviation roles of the classical state-function entropy, in the proper relation.

The dualization by Baish [101] of observable and response fields in Doi–Peliti theory stands in obvious analogy to the construction of the adjoint transition matrix in the fluctuation theorem for housekeeping heat [52,53]. The standardization of the CRN generator (35) makes the analogy explicit in the descaling (39) of T and the descaling (43) of A. Moreover, it shows the analogy as an expression of the general relation of microstates to macrostates, and relates the preservation or loss of information in the classical limit to CRN complexity classes.

The distinction between relaxation and escape trajectories in terms of formal momenta is widely developed in momentum-space WKB theory [78], as Hamilton–Jacobi theory [40], and in relation to information geometry [132]. The convexity proof in Equation (49) relating the Lyapunov to the large-deviation role of the classical-state entropy, and to the structure of the L=0 manifold, is novel.

Cycle decompositions of steady-state entropy rates now exist in many forms since Hill [76,107,109]. Here, a slightly more fine-grained cycle decomposition (keeping detailed-balanced currents) unifies all proofs of monotonicity of entropies for non-stationary distributions, relating the Lyapunov and large-deviation changes in macrostate entropy on complex-balanced CRNs in Equation (53), and its counterpart (61) in the state space for all CRNs. It also provides an interesting view of the (known) equivalence of the adjoints constructed in the generating function (64) for housekeeping heat in the environment, and an equivalent function for the relative entropy (61) in the system.

The Legendre duality of ${\dot{S}}^{\mathrm{HK}}$ to the potential ${\dot{F}}_{P}$ in Equation (81) defines extensivity and intensivity with respect to a different scaling than that for entropy, but a natural one if time-reversal and energy conservation are not the only basis for taking macroscopic limits.

## Figures and Tables

**Table 1:** Structures from micro to macro within and between levels. H-J refers to the Hamilton–Jacobi dynamical system that arises in the large-deviation approximation. Note that the distinction between micro**state** and macro**state** is a distinction of kind, which occurs within a level, whereas micro**scale**, meso**scale**, and macro**scale** are designations of relative levels with respect to large-deviation timescale separations. Terms in the same row are representations of the same quantity in different levels, related through coarse-graining.

Macroscale	Mesoscale	Microscale
	micro**state**	H-J fixed point
	thermalization of the microscale	H-J relaxation trajectories
	elementary transitions between microstates	H-J first-passages
	arbitrary distribution on microstates	coarse-grained distribution on fixed points
	macro**state** (distribution) ↔ H-J variables	
micro**state**	H-J fixed point	
…	…	

**Table 2:** Notation conventions adopted for three classes of distributions arising in large-deviation systems with multi-scale relaxation structure.

Notation	Definition	Comment
$\underline{\rho }$, ${\underline{\rho }}^{s}$, ${\underline{\rho }}^{e|s}$	whole-system, *s*-marginal, $\left(e|s\right)$-conditional distributions in the global stationary state	
${\bar{\rho }}^{s}$	marginal system steady-state distribution	function of instantaneous environment conditional distribution $\rho^{e|s}$
$\rho^{\left(\bar{n}\right)}$	macrostate tilted to saddle-point value $\bar{n}$	defined relative to global stationary distributions $\underline{\rho }$; may be defined for whole-system, *s*, or e∣s

**Table 3:** Hierarchical categories of stationary distributions of chemical reaction networks (CRNs).

Case	Complexity Class
$Y\hat{A}1=0$	general case
$\hat{A}1=0$	*complex-balanced*
${\hat{A}}^{T}=\hat{A}$	*detailed-balanced*

## Appendix A. On Distributions of Strings in a Mixture with Random Ligation and Hydrolysis

In a model concerned with linear (as opposed to branched) polymers, each monomer must have the capacity for double ligation. Suppose here that the monomers make directional links, as amino acids and nucleotides in standard $5^{\prime }-3^{\prime }$ anhydrides do.

Further, suppose that one ligation site of the pair on each monomer, which we term the donor, is activated (whether by an ambient process such as deprotonation or by an out-of-equilibrium leaving group) to initiate a ligation. Then, in an ensemble of polymers of variable length l≥1 (with l=1 corresponding to free monomers) the activity of donor sites is the total count of all polymers.

Denote by $\pi_{l}$ a polymer of length *l*, and let $n_{l}$ be the expected number of polymers of length *l*. Then, for any two lengths $l_{1}\neq l_{2}$, there are two channels interconverting polymers at those lengths with a polymer of length $l_{1}+l_{2}$ through ligation (in either order) or hydrolysis (at either asymmetric position), as

(A1) $$\begin{array}{cc} \pi_{l_{1}}+\pi_{l_{2}} & \overset{2k}{\underset{2\bar{k}}{⇌}}\pi_{l_{1}+l_{2}}. \end{array}$$

The corresponding steady-state rate-balance condition, with $K\equiv k/\bar{k}$, is

(A2) $$\begin{array}{cc} Kn_{l_{1}}n_{l_{2}} & =n_{l_{1}+l_{2}}. \end{array}$$

We will consider the range of large *l*, and not give separate consideration to the singular case $l_{1}=l_{2}$ where the hydrolysis rate is halved due to symmetry, or other special cases such as ligation to a ring.

The relations (A2) are satisfied for all pairs if the expected numbers have the form

(A3) $$\begin{array}{cc} n_{l}\; & =K^{\alpha l-1} \end{array}$$

for any α. Real α>0 are normalized for K<1 and α<0 are normalized for K>1.

The total polymer count and average length in the distribution (A3) is

(A4) $$\begin{array}{cc} \sum_{l\geq 1}n_{l} & =\frac{K^{\alpha -1}}{1-K^{\alpha }}\\ \langle l\rangle \; & =\frac{1}{1-K^{\alpha }} \end{array}$$

The number of anhydrous bonds in the ensemble, available to be cleaved, is

(A5) $$\begin{array}{cc} \sum_{l\geq 1}n_{l}\left(l-1\right)=\left(\sum_{l\geq 1}n_{l}\right)\langle l-1\rangle & =\frac{1}{K}{\left(\frac{K^{\alpha }}{1-K^{\alpha }}\right)}^{2} \end{array}$$

If this total bond number (A5) is set equal to *K*, then α is given by

(A6) $$\begin{array}{cc} \alpha \log K & =-\log\left(1+1/K\right). \end{array}$$

The activity of donor sites (free string ends) and expected length then become

(A7) $$\begin{array}{cc} \sum_{l\geq 1}n_{l}\; & =1\\ \langle l\rangle \; & =1+K \end{array}$$

If *K* is set equal to $K_{\mathrm{eff}}$ from Equation (69), then Equation (A7) recovers the input assumption of unit activity of species *A* and total ligated donor sites equal to $K_{\mathrm{eff}}$. Note that while in the Poisson process, n indexes total bonds, by Equation (A7), its expectation also characterizes expected string length.
